# Supplementary figures and images for: Reliability of multi-site UK Biobank MRI brain phenotypes for the assessment of neuropsychiatric complications of SARS-CoV-2 infection: The COVID-CNS travelling heads study
Source: PLoS One. 2022 Sep 29;17(9):e0273704. doi: 10.1371/journal.pone.0273704 (PMC9522299; doi:10.1371/journal.pone.0273704)

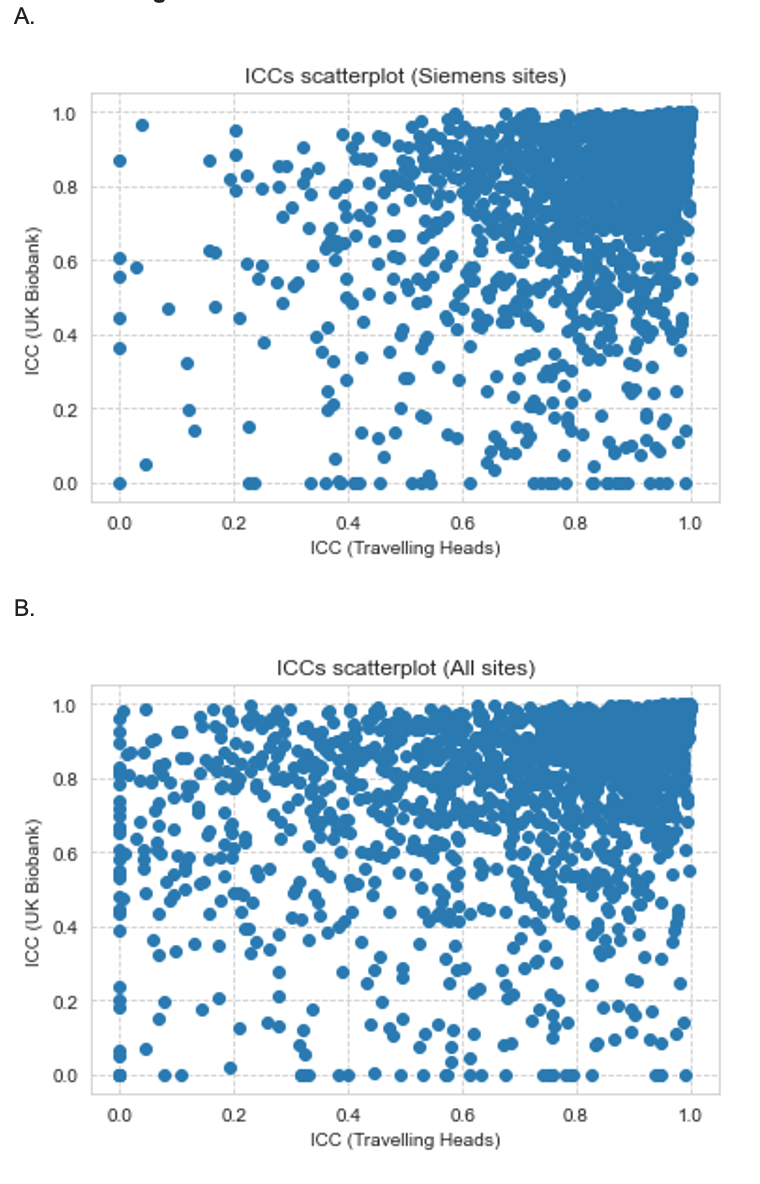

Supplement: S1 Fig — Scatterplots of Intra-class Correlation Coefficients (ICCs) for IDPs derived from UK Biobank repeat-scan data and Travelling Heads dataset A. Siemens sites (r = 0.43). B. All sites (r = 0.39). Each point corresponds to ICC for an individual IDP. (TIF) [file pone.0273704.s001.tif]

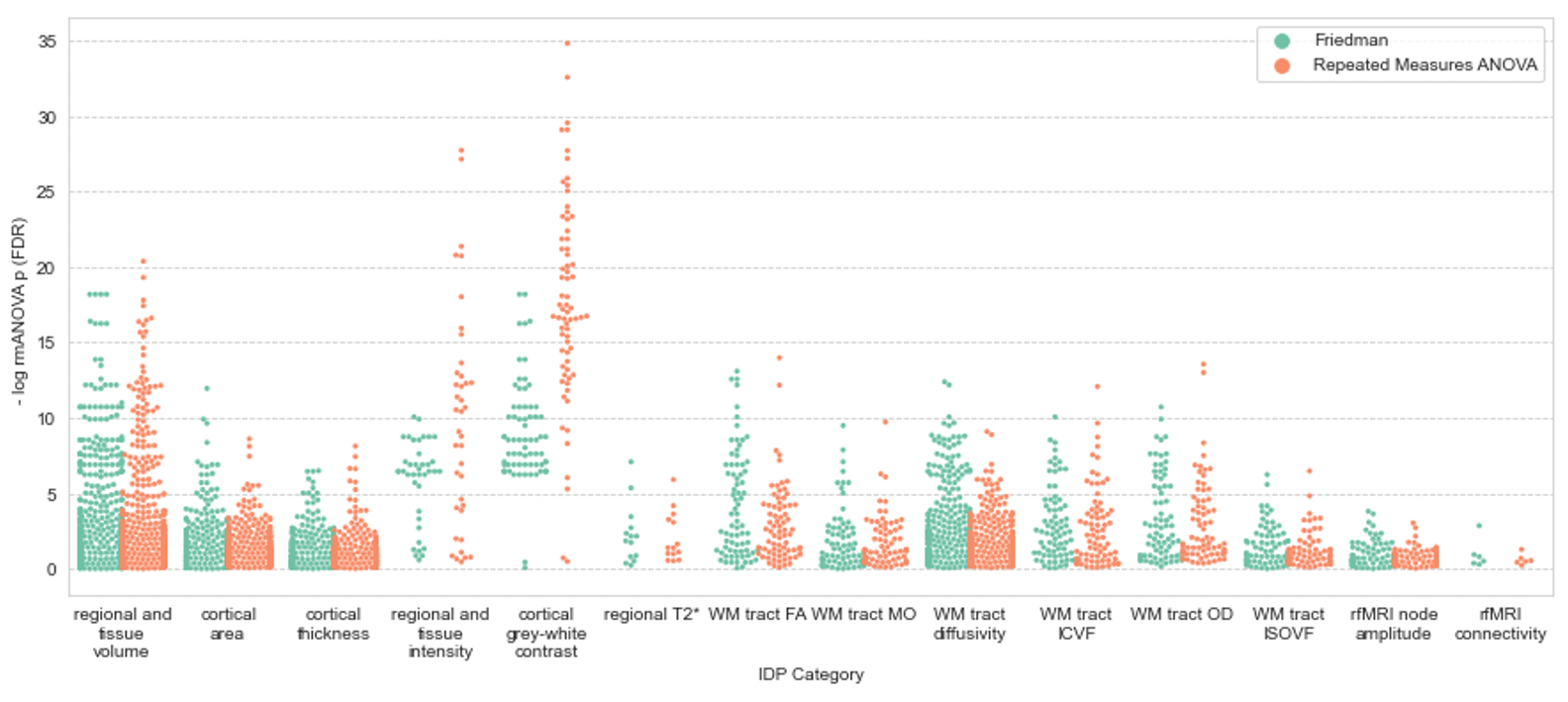

Supplement: S2 Fig — Comparison of FDR-corrected P-values for site effects derived using repeated measures ANOVA (orange) and non-parametric Friedman (green) tests. (TIF) [file pone.0273704.s002.tif]
