# Supplementary material for: Reliability of multi-site UK Biobank MRI brain phenotypes for the assessment of neuropsychiatric complications of SARS-CoV-2 infection: The COVID-CNS travelling heads study
Source: PLoS One. 2022 Sep 29;17(9):e0273704. doi: 10.1371/journal.pone.0273704 (PMC9522299; doi:10.1371/journal.pone.0273704)
Supplement: S1 Table — (DOCX) [file pone.0273704.s003.docx]

**S1 Table : Participant Information**

| Participant | Sex | Age | Height | Weight |
| --- | --- | --- | --- | --- |
| 0 | F | 21.0 | 1.67 | 57.0 |
| 1 | F | 24.0 | 1.7 | 70.0 |
| 2 | F | 21.0 | 1.58 | 59.0 |
| 3 | F | 20.0 | 1.6 | 55.0 |
| 4 | M | 20.0 | 1.75 | 64.0 |
| 5 | F | 38.0 | 1.76 | 60.0 |
| 6 | F | 24.0 | 1.7 | 72.0 |
| 7 | F | 20.0 | 1.55 | 90.0 |
