# Supplementary material for: Reliability of multi-site UK Biobank MRI brain phenotypes for the assessment of neuropsychiatric complications of SARS-CoV-2 infection: The COVID-CNS travelling heads study
Source: PLoS One. 2022 Sep 29;17(9):e0273704. doi: 10.1371/journal.pone.0273704 (PMC9522299; doi:10.1371/journal.pone.0273704)
Supplement: S1 File — (DOCX) [file pone.0273704.s004.docx]

# Covid-19 Clinical Neuroscience Study (COVID-CNS) Consortium Membership and Affiliation, 17 Dec 2021

John P Aggleton, School of Psychology, Cardiff University, Cardiff, UK.

Christopher M Allen, Mental Health and Clinical Neurosciences Academic Unit, School of Medicine, University of Nottingham, Nottingham, UK; Department of Neurology, Nottingham University Hospitals NHS Trust, Nottingham, UK.

Jay Amin, Faculty of Medicine, University of Southampton, Southampton, UK; Memory Assessment and Research Centre, Southern Health NHS Foundation Trust, Southampton, UK.

Paul Armitage, Department of Infection, Immunity and Cardiovascular Disease, Faculty of Medicine, University of Sheffield, Sheffield, UK.

Cherie Armour, School of Psychology, Queens University Belfast, Belfast, UK.

Dorothy Auer, Sir Peter Mansfield Imaging Centre, University of Nottingham Biomedical Research Centre, Nottingham, UK; NIHR Nottingham Biomedical Research Centre, Nottingham, UK.

Mark R Baker, Translational and Clinical Research Institute, Newcastle University, Newcastle upon Tyne, UK; Department of Clinical Neurophysiology, Royal Victoria Infirmary, Newcastle upon Tyne, UK.

Rahul Batra, Institute of Psychiatry, Psychology and Neuroscience, King’s College London, London, UK.

Suzanne Barrett, Northern Health and Social Care Trust, Antrim, UK.

Laura Benjamin, Stroke Research Centre, UCL Queen Square Institute of Neurology, University College London, London, UK; Brain Infections Group, University of Liverpool, Liverpool, UK.

Alex Berry, Division of Psychiatry, University College London (UCL), Bloomsbury, UK.

Richard A I Bethlehem, Brain Mapping Unit, Department of Psychiatry, University of Cambridge, Cambridge, UK; Autism Research Centre, Department of Psychiatry, University of Cambridge, Cambridge, UK.

John Bradley, Department of Medicine, University of Cambridge, Cambridge, UK; Addenbrooke's Hospital and NIHR Cambridge Biomedical Research Centre, Cambridge, UK.

David P Breen, Centre for Clinical Brain Sciences, University of Edinburgh, Edinburgh, UK; Department of Clinical Neurosciences, Royal Infirmary of Edinburgh, Edinburgh, UK; Anne Rowling Regenerative Neurology Clinic, University of Edinburgh, Edinburgh, UK.

Gerome Breen, Institute of Psychiatry, Psychology and Neuroscience, King's College London, London, UK; UK National Institute for Health Research (NIHR) Biomedical Research Centre, South London and Maudsley Hospital, London, UK.

Judith Breuer, Infection, Immunity and Inflammation, Great Ormond Street Institute of Child Health, University College London, London, UK.

Matthew R Broome, Institute for Mental Health, School of Psychology, University of Birmingham, Birmingham, UK; Early Intervention in Psychosis Service, Birmingham Women's and Children's NHS Foundation Trust, Birmingham, UK.

Edward Bullmore, Department of Psychiatry and Wolfson Brain Imaging Centre, Department of Clinical Neurosciences, University of Cambridge, Cambridge, UK; R&D, Cambridgeshire & Peterborough NHS Foundation Trust, Cambridge, UK.

Matt Butler, Institute of Psychiatry, Psychology and Neuroscience, King’s College London, London, UK.

Georgina Carr, Neurological alliance, Watford, UK.

Alan Carson, Centre of Clinical Brain Sciences, University of Edinburgh, UK; Robert Fergusson Unit, Royal Edinburgh Hospital, UK.

Hannah Castell, Department of Clinical Infection, Microbiology and Immunology, Institute of Infection, Veterinary and Ecological Sciences, University of Liverpool, Liverpool, UK.

Jonathan Cavanagh, Centre for Immunobiology, University of Glasgow, Glasgow, UK.

Patrick Chinnery, Department of Clinical Neurosciences, School of Clinical Medicine, University of Cambridge, Cambridge, UK.

Alasdair Coles, Department of Clinical Neurosciences, University of Cambridge, Cambridge, UK.

Ceryce Collie, Department of Clinical Infection, Microbiology and Immunology, Institute of Infection, Veterinary and Ecological Sciences, University of Liverpool, Liverpool, UK.

David Cousins, Institute of Neuroscience, Newcastle University, Newcastle upon Tyne, UK; Northumberland Tyne and Wear National Health Service Foundation Trust, Newcastle upon Tyne, UK.

David Christmas, Department of Psychiatry, University of Cambridge, Cambridge, UK; Cambridgeshire and Peterborough NHS Foundation Trust, Cambridge, UK.

Alistair Darby, Institute of Infection, Veterinary and Ecological Sciences, University of Liverpool, Liverpool, UK.

Anthony S. David, Institute of Mental Health, University College London, London, UK; Department of Neuropsychiatry, National Hospital for Neurology and Neurosurgery, London, UK.

Nicholas Davies, Department of Infectious Disease Epidemiology, London School of Hygiene & Tropical Medicine, London, UK.

Sylviane Defres, Department of Clinical Infection, Medical Microbiology and Immunology, University of Liverpool, Liverpool, UK; Tropical and Infectious Diseases Unit, Liverpool University Hospitals Foundation Trust, Liverpool, UK.

Katherine C Dodd, Division of Neuroscience and Experimental Psychology, University of Manchester, Manchester, UK; Manchester Centre for Clinical Neuroscience, Salford Royal NHS Foundation Trust, Salford, UK.

Eugene Duff, Centre for Functional MRI of the Brain, Wellcome Centre for Integrative Neuroimaging, University of Oxford, Oxford, UK; Department of Paediatrics, University of Oxford, Oxford, UK; UK Dementia Research Institute, Department of Brain Sciences, Imperial College London, London, UK.

Cordelia Dunai, Clinical Infection, Microbiology & Immunology, Institute of Infection, Veterinary and Ecological Sciences, University of Liverpool, Liverpool, UK.

Ava Easton, Department of Clinical Infection, Microbiology and Immunology, Institute of Infection, Veterinary and Ecological Sciences, University of Liverpool, Liverpool, UK; Encephalitis Society, Malton, UK.

John Evans, CUBRIC, School of Psychology, Cardiff University, Cardiff, UK.

Bethany Facer, Institute of Systems, Molecular and Integrative Biology, University of Liverpool, Liverpool, UK.

Richard Francis, Stroke Association, Northampton, UK.

Ian Galea, Clinical Neurosciences, Clinical and Experimental Sciences, Faculty of Medicine, University of Southampton, Southampton, UK; Wessex Neurological Centre, University Hospital Southampton National Health Service Foundation Trust, Southampton, UK.

Afagh Garjani, Mental Health and Clinical Neurosciences Academic Unit, School of Medicine, University of Nottingham, Nottingham, UK; Department of Clinical Neurology, Nottingham University Hospitals NHS Trust, Nottingham, UK.

Lily George, Institute of Psychiatry, Psychology and Neuroscience, King’s College London, London, UK.

Kiran Glen, Social, Genetic and Developmental Psychiatry Centre, Institute of Psychiatry, Psychology and Neuroscience, King’s College London, London, UK.

Michael Griffiths, Clinical Infection, Microbiology & Immunology, Institute of Infection, Veterinary and Ecological Sciences, University of Liverpool, Liverpool, UK.

Victoria Grimbly, Institute of Infection, Veterinary and Ecological Sciences, University of Liverpool, Liverpool, UK.

Alexander Grundmann, CENS, University of Southampton; Southampton, UK; Department of Neurology, Wessex Neurological Centre, Southampton, UK.

Shahd H M Hamid, Neurology Department, The Walton Centre NHS Foundation Trust, Liverpool, UK; Faculty of Health and Life Sciences, University of Liverpool, Liverpool, UK.

Adam Hampshire, Faculty of Medicine, Department of Brain Sciences, Imperial College London, London, UK.

Ewan Harrison, Wellcome Sanger Institute, Hinxton, UK; Department of Medicine, University of Cambridge, Cambridge, UK; Department of Public Health and Primary Care, University of Cambridge, Cambridge, UK.

Neil A Harrison, Department of Psychiatry and Clinical Neurosciences, Cardiff University, Cardiff, UK; Psychiatry, Cardiff and Vale Health Board, Cardiff, UK.

Paul J Harrison, Department of Psychiatry, University of Oxford, Oxford, UK; Oxford Health NHS Foundation Trust, Oxford, UK.

Nicholas Hart, Lane Fox Clinical Respiratory Physiology Research Centre, St Thomas' Hospital, Guy's and St Thomas' Foundation Trust, London, London, UK.

Monika Hartmann, Institute of Psychiatry, Psychology and Neuroscience, King’s College London, London, UK.

Julian A Hiscox, Institute of Infection, Veterinary and Ecological Sciences, University of Liverpool, Liverpool, UK.

Eva Maria Hodel, Institute of Infection, Veterinary and Ecological Sciences, University of Liverpool, Liverpool, UK.

Nigel Hoggard, Institute for in silico Medicine (INSIGNEO), University of Sheffield, Sheffield, UK; Academic Unit of Radiology, Royal Hallamshire Hospital, University of Sheffield, Sheffield, UK

Matthew Hotopf, Department of Psychological Medicine, Institute of Psychiatry, Psychology and Neuroscience, King's College London, London, UK; Biomedical Research Centre, South London and Maudsley NHS Foundation Trust, London, UK.

Topher Hübel, Social, Genetic & Developmental Psychiatry Centre, Institute of Psychiatry, Psychology & Neuroscience, King’s College London, London, UK

Stella Hughes, Neurology, Belfast Health and Social Care Trust, Belfast, UK. Masud Husain, Nuffield Department of Clinical Neurosciences, University of Oxford, Oxford, UK; Oxford University Hospitals NHS Foundation Trust, Oxford, UK.

Sarosh Irani, Nuffield Department of Clinical Neurosciences, University of Oxford, Oxford, UK; Department of Neurology, John Radcliffe Hospital, Oxford University Hospitals NHS Foundation Trust, Oxford, UK.

Thomas Jackson, Institute of Inflammation and Ageing, University of Birmingham, Birmingham, UK.

Hans Rolf Jäger, UCL Queen Square Institute of Neurology, University College London, London, UK.

Thomas M Jenkins, Sheffield Institute for Translational Neuroscience, University of Sheffield, Sheffield, UK; Department of Neurology, Sheffield Teaching Hospitals NHS Foundation Trust, Sheffield, UK.

Peter Jezzard, Wellcome Centre for Integrative Neuroimaging, FMRIB Division, Nuffield Department of Clinical Neurosciences, University of Oxford, Oxford, UK.

Gursharan Kalsi, Institute of Psychiatry, Psychology and Neuroscience, King's College London, London, UK; National Institute for Health Research Biomedical Research Centre, South London and Maudsley Hospital, London, UK.

Tasmyn Kapfumvuti, Encephalitis Society, Malton, UK.

Simon Keller, Institute of Systems, Molecular and Integrative Biology, University of Liverpool, Liverpool, UK; The Walton Centre NHS Foundation Trust, Liverpool, UK.

Sander Kyaw, Nottingham Healthcare NHS Foundation Trust, Nottingham, UK.

Charles Leek, Institute of Population Health, University of Liverpool, Liverpool, UK. Gabriella Lewis, South London and Maudsley NHS Foundation Trust, London, UK.

James B Lilleker, Centre for Musculoskeletal Research, University of Manchester, Manchester, UK; Manchester Centre for Clinical Neurosciences, Salford Royal NHS Foundation Trust, Salford, UK.

Claire MacIver, Neuroscience and Mental Health Research Institute, Cardiff University, Cardiff, UK.

Mika Malouf, Institute of Psychiatry, Psychology and Neuroscience, King’s College London, London, UK.

Naomi Martin, Institute of Psychiatry, Psychology and Neuroscience, King’s College London, London, UK.

Gavin V McDonnell, Department of Neurology, Belfast City Hospital, Northern Ireland, UK.

Ryan McIlwaine, Queen’s University Belfast, Belfast, UK.

Andrew M McIntosh, Division of Psychiatry, University of Edinburgh; NHS Lothian, Royal Edinburgh Hospital

David K Menon, Division of Anaesthesia, Department of Medicine, University of Cambridge, Cambridge, UK; Neurosciences/Trauma Critical Care Unit, Addenbrooke's Hospital, Cambridge, UK; Wolfson Brain Imaging Centre, University of Cambridge, Cambridge, UK.

Benedict D Michael, Department of Clinical Infection Microbiology and Immunology, Institute of Infection, Veterinary, and Ecological Sciences, University of Liverpool, Liverpool, UK; The National Institute for Health Research Health Protection Research Unit for Emerging and Zoonotic Infections, University of Liverpool, Liverpool, UK; Department of Neurology, The Walton Centre NHS Foundation Trust, Liverpool, UK.

Karla Miller, Centre for Functional MRI of the Brain, Wellcome Centre for Integrative Neuroimaging, University of Oxford, Oxford, UK.

Lea Milligan, MQ Mental Health Research, London, UK.

Dina Monssen, Social, Genetic and Developmental Psychiatry Centre, Institute of Psychiatry, Psychology and Neuroscience, King’s College London, London, UK.

Ciaran Mulholland, Centre for Evidence and Social Innovation, Queen’s University Belfast, Belfast, UK.

Akshay Nair, Huntington’s Disease Centre, Queen Square Institute of Neurology, University College London, London, UK.

Maaz Nayyer, Sheffield Institute for Translational Neuroscience, University of Sheffield, Sheffield, UK.

Edward Needham, Department of Clinical Neurosciences, University of Cambridge, Cambridge, UK; Department of Neurology, Cambridge University Hospital, Cambridge, UK. Timothy Nicholson, Institute of Psychiatry, Psychology and Neuroscience, King's College London, London, UK.

Natalie Nicholas, Liverpool University Hospital NHS Foundation Trust, Liverpool, UK.

Neil Nixon, Division of Psychiatry and Applied Psychology, School of Medicine, University of Nottingham, Nottingham, UK; Adult Mental Health Directorate, Nottinghamshire Healthcare Trust, Nottingham, UK.

Obioma Orazulume, The National Hospital for Neurology and Neurosurgery, Queens Square, London, UK.

Marlies Ostermann, Department of Intensive Care, Guy’s & St Thomas’ NHS Foundation Hospital, London, UK

Stella-Maria Paddick, Clinical and translational medicine, Newcastle University, Newcastle upon Tyne, UK; Old Age Psychiatry, Gateshead Health NHS Foundation Trust, Gateshead, UK.

Tom Pollak, Department of Psychosis Studies, Institute of Psychiatry, Psychology and Neuroscience, King's College London, London, UK; South London and Maudsley NHS Foundation Trust, University College London, London, UK.

Alish Palmos, Social, Genetic and Developmental Psychiatry Centre, King’s College London, London, UK.

Arvind Patel, MRC-University of Glasgow Centre for Virus Research, University of Glasgow, Glasgow, UK.

Sharon J Peacock, Department of Medicine, University of Cambridge, Cambridge, UK. Wellcome Sanger Institute, Hinxton, Cambridge, UK.

Sophie Pendered, Department of Clinical Infection, Microbiology and Immunology, Institute of Infection, Veterinary and Ecological Sciences, University of Liverpool, Liverpool, UK.

Sarah Pett, MRC CTU at UCL, Institute of Clinical Trials and Methodology, University College London, London, UK. Angela Roberts, Department of Physiology, Development and Neuroscience, Behavioural and Clinical Neuroscience Institute, University of Cambridge, Cambridge, UK.

Henry C Rogers, Social, Genetic and Developmental Psychiatry Centre, Institute of Psychiatry, Psychology and Neuroscience, King’s College London, London, UK; National Institute for Health Research Biomedical Research Centre, South London and Maudsley Hospital, London, UK.

Silvia Rota, Institute of Psychiatry, Psychology & Neuroscience, King’s College London, London, UK; Parkinson’s Foundation Centre of Excellence, King’s College London, London, UK.

Merna Samuel, Infectious Diseases, Royal Liverpool University Hospital, Liverpool, UK.

Stephen Sawcer, Department of Clinical Neurosciences, University of Cambridge, University of Cambridge, Cambridge, UK; Cambridge University Hospital NHS Foundation Trust, Cambridge, UK.

Adam W Seed, Department of Medicine for Older People & Stroke, University of Liverpool, UK.

Pamela J Shaw, Sheffield Institute for Translational Neuroscience, University of Sheffield, Sheffield, UK; Sheffield NIHR Biomedical Research Centre, Sheffield Teaching Hospitals NHS Foundation Trust, Sheffield, UK.

Bhagteshwar Singh, National Institute for Health Research Health Protection Research Unit in Emerging and Zoonotic Infections, Institute of Infection, Veterinary and Ecological Sciences, University of Liverpool, Liverpool, UK; Tropical and Infectious Diseases Unit, Royal Liverpool and Broadgreen University Hospitals NHS Trust, Liverpool, UK; Christian Medical College, Vellore, India.

Rekha Siripurapu, Department of Neuroradiology, Manchester Centre for Clinical Neurosciences, Salford NHS Foundation Trust, Salford, UK.

Craig J Smith, Division of Cardiovascular Sciences; Faculty of Biology, Medicine and Health; Geoffrey Jefferson Brain Research Centre, University of Manchester, Manchester, UK; Hyperacute Stroke Unit, Manchester Centre for Clinical Neurosciences, Salford, UK.

Stephen Smith, Wellcome Centre for Integrative Neuroimaging (WIN FMRIB), University of Oxford, Oxford, UK.

Tom Solomon, National Institute for Health Research (NIHR) Health Protection Research Unit in Emerging and Zoonotic Infections, Institute of Infection Veterinary and Ecological Science, University of Liverpool, Liverpool, UK; Department of Neurology, The Walton Centre NHS Foundation Trust, Liverpool, UK.

Leonie Taams, Inflammation Biology, School of Immunology & Microbial Sciences, King’s College London, London, UK; Centre for Inflammation Biology & Cancer Immunology, King’s College London, London, UK.

Esteban Tato-Barcia, National Institute for Health Research Biomedical Research Centre, South London and Maudsley Hospital, London, UK.

John-Paul Taylor, Translational and Clinical Research Institute, Newcastle University, Newcastle upon Tyne, UK; Cumbria Northumberland Tyne and Wear NHS Foundation Trust, Newcastle upon Tyne, UK.

Kukatharmini Tharmaratnam, Institute of Population Health, University of Liverpool, Liverpool, UK.

Rhys H Thomas, Translational and Clinical Research, Newcastle University, Newcastle upon Tyne, UK; Neurology Department, Royal Victoria Infirmary, Newcastle upon Tyne, UK.

Emma Thomson, MRC ‐ University of Glasgow Centre for Virus Research, University of Glasgow, Glasgow, UK.

Jonathan Underwood, Division of Infection and Immunity, Cardiff University, Cardiff, UK; Department of Infectious Diseases, Cardiff and Vale University Health Board, Cardiff, UK.

Rachel Upthegrove, Institute for Mental Health, University of Birmingham, Birmingham, UK.

Tom Vale, Nuffield Department of Clinical Neurosciences, University of Oxford, Oxford, UK.

Daniel J van Wamelen, Institute of Psychiatry Psychology and Neuroscience, King's College London, London, UK; Parkinson Foundation Center of Excellence, King's College Hospital, London, UK; Department of Neurology, Donders Institute for Neuroscience, Radboud University Medical Centre, Nijmegen, Netherlands.

Angela Vincent, Nuffield Department of Clinical Neurosciences, University of Oxford, Oxford, UK.

Guy B Williams, Wolfson Brain Imaging Centre, University of Cambridge, Cambridge, UK.

Steve Williams, Department of Neuroimaging, Institute of Psychiatry, Psychology and Neuroscience, King's College London, UK.

Glynn Webb, Institute of Infection, Veterinary and Ecological Sciences, University of Liverpool, Liverpool, UK; Liverpool University Hospital NHS Foundation Trust, Liverpool, UK.

Sui Wong, Department of Neuro-Ophthalmology, Moorfields Eye Hospital, Guys and St Thomas' NHSFT, London, UK.

Greta Wood, Clinical Infection, Microbiology & Immunology, Institute of Infection, Veterinary and Ecological Sciences, University of Liverpool, Liverpool, UK.

Nicholas Wood, Department of Clinical and Movement Neurosciences, UCL Queen Square Institute of Neurology, University College London, London, UK; UCL Movement Disorders Centre, University College London, London, UK.

Michael S Zandi, Queen Square Institute of Neurology, University College London, London, UK; Neuroimmunology, National Hospital for Neurology and Neurosurgery, Queen Square, UCLH, London, UK.

Fernando Zelaya, Department of Neuroimaging, Institute of Psychiatry, Psychology and Neuroscience, King’s College London, London, UK.

Johan K Zvrskovec, Social Genetic & Developmental Psychiatry, Institute of Psychiatry, Psychology and Neuroscience, King’s College London, London, UK.
