# Supplementary material for: Reliability of multi-site UK Biobank MRI brain phenotypes for the assessment of neuropsychiatric complications of SARS-CoV-2 infection: The COVID-CNS travelling heads study
Source: PLoS One. 2022 Sep 29;17(9):e0273704. doi: 10.1371/journal.pone.0273704 (PMC9522299; doi:10.1371/journal.pone.0273704)
Supplement: S2 File — (DOCX) [file pone.0273704.s005.docx]

**List of IDPs**

Further information on IDPs can be found at: <https://biobank.ctsu.ox.ac.uk/showcase/search.cgi>

IDP name, IDP category, IDP category name

IDP_T1_SIENAX_peripheral_grey_normalised_volume, 1, regional and tissue volume

IDP_T1_SIENAX_peripheral_grey_unnormalised_volume, 1, regional and tissue volume

IDP_T1_SIENAX_CSF_normalised_volume, 1, regional and tissue volume

IDP_T1_SIENAX_CSF_unnormalised_volume, 1, regional and tissue volume

IDP_T1_SIENAX_grey_normalised_volume, 1, regional and tissue volume

IDP_T1_SIENAX_grey_unnormalised_volume, 1, regional and tissue volume

IDP_T1_SIENAX_white_normalised_volume, 1, regional and tissue volume

IDP_T1_SIENAX_white_unnormalised_volume, 1, regional and tissue volume

IDP_T1_SIENAX_brain-normalised_volume, 1, regional and tissue volume

IDP_T1_SIENAX_brain-unnormalised_volume, 1, regional and tissue volume

IDP_T1_FIRST_left_thalamus_volume, 1, regional and tissue volume

IDP_T1_FIRST_right_thalamus_volume, 1, regional and tissue volume

IDP_T1_FIRST_left_caudate_volume, 1, regional and tissue volume

IDP_T1_FIRST_right_caudate_volume, 1, regional and tissue volume

IDP_T1_FIRST_left_putamen_volume, 1, regional and tissue volume

IDP_T1_FIRST_right_putamen_volume, 1, regional and tissue volume

IDP_T1_FIRST_left_pallidum_volume, 1, regional and tissue volume

IDP_T1_FIRST_right_pallidum_volume, 1, regional and tissue volume

IDP_T1_FIRST_left_hippocampus_volume, 1, regional and tissue volume

IDP_T1_FIRST_right_hippocampus_volume, 1, regional and tissue volume

IDP_T1_FIRST_left_amygdala_volume, 1, regional and tissue volume

IDP_T1_FIRST_right_amygdala_volume, 1, regional and tissue volume

IDP_T1_FIRST_left_accumbens_volume, 1, regional and tissue volume

IDP_T1_FIRST_right_accumbens_volume, 1, regional and tissue volume

IDP_T1_FIRST_brain_stem+4th_ventricle_volume, 1, regional and tissue volume

IDP_T1_FAST_ROIs_L_frontal_pole, 1, regional and tissue volume

IDP_T1_FAST_ROIs_R_frontal_pole, 1, regional and tissue volume

IDP_T1_FAST_ROIs_L_insular_cortex, 1, regional and tissue volume

IDP_T1_FAST_ROIs_R_insular_cortex, 1, regional and tissue volume

IDP_T1_FAST_ROIs_L_sup_front_gyrus, 1, regional and tissue volume

IDP_T1_FAST_ROIs_R_sup_front_gyrus, 1, regional and tissue volume

IDP_T1_FAST_ROIs_L_mid_front_gyrus, 1, regional and tissue volume

IDP_T1_FAST_ROIs_R_mid_front_gyrus, 1, regional and tissue volume

IDP_T1_FAST_ROIs_L_inf_front_gyrus_parstri, 1, regional and tissue volume

IDP_T1_FAST_ROIs_R_inf_front_gyrus_parstri, 1, regional and tissue volume

IDP_T1_FAST_ROIs_L_inf_front_gyrus_parsop, 1, regional and tissue volume

IDP_T1_FAST_ROIs_R_inf_front_gyrus_parsop, 1, regional and tissue volume

IDP_T1_FAST_ROIs_L_precentral_gyrus, 1, regional and tissue volume

IDP_T1_FAST_ROIs_R_precentral_gyrus, 1, regional and tissue volume

IDP_T1_FAST_ROIs_L_temporal_pole, 1, regional and tissue volume

IDP_T1_FAST_ROIs_R_temporal_pole, 1, regional and tissue volume

IDP_T1_FAST_ROIs_L_sup_temp_gyrus_ant, 1, regional and tissue volume

IDP_T1_FAST_ROIs_R_sup_temp_gyrus_ant, 1, regional and tissue volume

IDP_T1_FAST_ROIs_L_sup_temp_gyrus_post, 1, regional and tissue volume

IDP_T1_FAST_ROIs_R_sup_temp_gyrus_post, 1, regional and tissue volume

IDP_T1_FAST_ROIs_L_mid_temp_gyrus_ant, 1, regional and tissue volume

IDP_T1_FAST_ROIs_R_mid_temp_gyrus_ant, 1, regional and tissue volume

IDP_T1_FAST_ROIs_L_mid_temp_gyrus_post, 1, regional and tissue volume

IDP_T1_FAST_ROIs_R_mid_temp_gyrus_post, 1, regional and tissue volume

IDP_T1_FAST_ROIs_L_mid_temp_gyrus_tempocc, 1, regional and tissue volume

IDP_T1_FAST_ROIs_R_mid_temp_gyrus_tempocc, 1, regional and tissue volume

IDP_T1_FAST_ROIs_L_inf_temp_gyrus_ant, 1, regional and tissue volume

IDP_T1_FAST_ROIs_R_inf_temp_gyrus_ant, 1, regional and tissue volume

IDP_T1_FAST_ROIs_L_inf_temp_gyrus_post, 1, regional and tissue volume

IDP_T1_FAST_ROIs_R_inf_temp_gyrus_post, 1, regional and tissue volume

IDP_T1_FAST_ROIs_L_inf_temp_gyrus_tempocc, 1, regional and tissue volume

IDP_T1_FAST_ROIs_R_inf_temp_gyrus_tempocc, 1, regional and tissue volume

IDP_T1_FAST_ROIs_L_postcent_gyrus, 1, regional and tissue volume

IDP_T1_FAST_ROIs_R_postcent_gyrus, 1, regional and tissue volume

IDP_T1_FAST_ROIs_L_sup_parietal_lobule, 1, regional and tissue volume

IDP_T1_FAST_ROIs_R_sup_parietal_lobule, 1, regional and tissue volume

IDP_T1_FAST_ROIs_L_supramarg_gyrus_ant, 1, regional and tissue volume

IDP_T1_FAST_ROIs_R_supramarg_gyrus_ant, 1, regional and tissue volume

IDP_T1_FAST_ROIs_L_supramarg_gyrus_post, 1, regional and tissue volume

IDP_T1_FAST_ROIs_R_supramarg_gyrus_post, 1, regional and tissue volume

IDP_T1_FAST_ROIs_L_angular_gyrus, 1, regional and tissue volume

IDP_T1_FAST_ROIs_R_angular_gyrus, 1, regional and tissue volume

IDP_T1_FAST_ROIs_L_latocc_cortex_sup, 1, regional and tissue volume

IDP_T1_FAST_ROIs_R_latocc_cortex_sup, 1, regional and tissue volume

IDP_T1_FAST_ROIs_L_latocc_cortex_inf, 1, regional and tissue volume

IDP_T1_FAST_ROIs_R_latocc_cortex_inf, 1, regional and tissue volume

IDP_T1_FAST_ROIs_L_intracalc_cortex, 1, regional and tissue volume

IDP_T1_FAST_ROIs_R_intracalc_cortex, 1, regional and tissue volume

IDP_T1_FAST_ROIs_L_front_med_cortex, 1, regional and tissue volume

IDP_T1_FAST_ROIs_R_front_med_cortex, 1, regional and tissue volume

IDP_T1_FAST_ROIs_L_juxtapos_lobule_cortex, 1, regional and tissue volume

IDP_T1_FAST_ROIs_R_juxtapos_lobule_cortex, 1, regional and tissue volume

IDP_T1_FAST_ROIs_L_subcallosal_cortex, 1, regional and tissue volume

IDP_T1_FAST_ROIs_R_subcallosal_cortex, 1, regional and tissue volume

IDP_T1_FAST_ROIs_L_paracing_gyrus, 1, regional and tissue volume

IDP_T1_FAST_ROIs_R_paracing_gyrus, 1, regional and tissue volume

IDP_T1_FAST_ROIs_L_cing_gyrus_ant, 1, regional and tissue volume

IDP_T1_FAST_ROIs_R_cing_gyrus_ant, 1, regional and tissue volume

IDP_T1_FAST_ROIs_L_cing_gyrus_post, 1, regional and tissue volume

IDP_T1_FAST_ROIs_R_cing_gyrus_post, 1, regional and tissue volume

IDP_T1_FAST_ROIs_L_precun_cortex, 1, regional and tissue volume

IDP_T1_FAST_ROIs_R_precun_cortex, 1, regional and tissue volume

IDP_T1_FAST_ROIs_L_cuneal_cortex, 1, regional and tissue volume

IDP_T1_FAST_ROIs_R_cuneal_cortex, 1, regional and tissue volume

IDP_T1_FAST_ROIs_L_front_orb_cortex, 1, regional and tissue volume

IDP_T1_FAST_ROIs_R_front_orb_cortex, 1, regional and tissue volume

IDP_T1_FAST_ROIs_L_parahipp_gyrus_ant, 1, regional and tissue volume

IDP_T1_FAST_ROIs_R_parahipp_gyrus_ant, 1, regional and tissue volume

IDP_T1_FAST_ROIs_L_parahipp_gyrus_post, 1, regional and tissue volume

IDP_T1_FAST_ROIs_R_parahipp_gyrus_post, 1, regional and tissue volume

IDP_T1_FAST_ROIs_L_lingual_gyrus, 1, regional and tissue volume

IDP_T1_FAST_ROIs_R_lingual_gyrus, 1, regional and tissue volume

IDP_T1_FAST_ROIs_L_temp_fusif_cortex_ant, 1, regional and tissue volume

IDP_T1_FAST_ROIs_R_temp_fusif_cortex_ant, 1, regional and tissue volume

IDP_T1_FAST_ROIs_L_temp_fusif_cortex_post, 1, regional and tissue volume

IDP_T1_FAST_ROIs_R_temp_fusif_cortex_post, 1, regional and tissue volume

IDP_T1_FAST_ROIs_L_temp_occ_fusif_cortex, 1, regional and tissue volume

IDP_T1_FAST_ROIs_R_temp_occ_fusif_cortex, 1, regional and tissue volume

IDP_T1_FAST_ROIs_L_occ_fusif_gyrus, 1, regional and tissue volume

IDP_T1_FAST_ROIs_R_occ_fusif_gyrus, 1, regional and tissue volume

IDP_T1_FAST_ROIs_L_front_operc_cortex, 1, regional and tissue volume

IDP_T1_FAST_ROIs_R_front_operc_cortex, 1, regional and tissue volume

IDP_T1_FAST_ROIs_L_cent_operc_cortex, 1, regional and tissue volume

IDP_T1_FAST_ROIs_R_cent_operc_cortex, 1, regional and tissue volume

IDP_T1_FAST_ROIs_L_parietal_operc_cortex, 1, regional and tissue volume

IDP_T1_FAST_ROIs_R_parietal_operc_cortex, 1, regional and tissue volume

IDP_T1_FAST_ROIs_L_planum_polare, 1, regional and tissue volume

IDP_T1_FAST_ROIs_R_planum_polare, 1, regional and tissue volume

IDP_T1_FAST_ROIs_L_heschl_gyrus, 1, regional and tissue volume

IDP_T1_FAST_ROIs_R_heschl_gyrus, 1, regional and tissue volume

IDP_T1_FAST_ROIs_L_planum_temporale, 1, regional and tissue volume

IDP_T1_FAST_ROIs_R_planum_temporale, 1, regional and tissue volume

IDP_T1_FAST_ROIs_L_supracalc_cortex, 1, regional and tissue volume

IDP_T1_FAST_ROIs_R_supracalc_cortex, 1, regional and tissue volume

IDP_T1_FAST_ROIs_L_occ_pole, 1, regional and tissue volume

IDP_T1_FAST_ROIs_R_occ_pole, 1, regional and tissue volume

IDP_T1_FAST_ROIs_L_thalamus, 1, regional and tissue volume

IDP_T1_FAST_ROIs_R_thalamus, 1, regional and tissue volume

IDP_T1_FAST_ROIs_L_caudate, 1, regional and tissue volume

IDP_T1_FAST_ROIs_R_caudate, 1, regional and tissue volume

IDP_T1_FAST_ROIs_L_putamen, 1, regional and tissue volume

IDP_T1_FAST_ROIs_R_putamen, 1, regional and tissue volume

IDP_T1_FAST_ROIs_L_pallidum, 1, regional and tissue volume

IDP_T1_FAST_ROIs_R_pallidum, 1, regional and tissue volume

IDP_T1_FAST_ROIs_L_hippocampus, 1, regional and tissue volume

IDP_T1_FAST_ROIs_R_hippocampus, 1, regional and tissue volume

IDP_T1_FAST_ROIs_L_amygdala, 1, regional and tissue volume

IDP_T1_FAST_ROIs_R_amygdala, 1, regional and tissue volume

IDP_T1_FAST_ROIs_L_ventral_striatum, 1, regional and tissue volume

IDP_T1_FAST_ROIs_R_ventral_striatum, 1, regional and tissue volume

IDP_T1_FAST_ROIs_brain_stem, 1, regional and tissue volume

IDP_T1_FAST_ROIs_L_cerebellum_I-IV, 1, regional and tissue volume

IDP_T1_FAST_ROIs_R_cerebellum_I-IV, 1, regional and tissue volume

IDP_T1_FAST_ROIs_L_cerebellum_V, 1, regional and tissue volume

IDP_T1_FAST_ROIs_R_cerebellum_V, 1, regional and tissue volume

IDP_T1_FAST_ROIs_L_cerebellum_VI, 1, regional and tissue volume

IDP_T1_FAST_ROIs_V_cerebellum_VI, 1, regional and tissue volume

IDP_T1_FAST_ROIs_R_cerebellum_VI, 1, regional and tissue volume

IDP_T1_FAST_ROIs_L_cerebellum_crus_I, 1, regional and tissue volume

IDP_T1_FAST_ROIs_V_cerebellum_crus_I, 1, regional and tissue volume

IDP_T1_FAST_ROIs_R_cerebellum_crus_I, 1, regional and tissue volume

IDP_T1_FAST_ROIs_L_cerebellum_crus_II, 1, regional and tissue volume

IDP_T1_FAST_ROIs_V_cerebellum_crus_II, 1, regional and tissue volume

IDP_T1_FAST_ROIs_R_cerebellum_crus_II, 1, regional and tissue volume

IDP_T1_FAST_ROIs_L_cerebellum_VIIb, 1, regional and tissue volume

IDP_T1_FAST_ROIs_V_cerebellum_VIIb, 1, regional and tissue volume

IDP_T1_FAST_ROIs_R_cerebellum_VIIb, 1, regional and tissue volume

IDP_T1_FAST_ROIs_L_cerebellum_VIIIa, 1, regional and tissue volume

IDP_T1_FAST_ROIs_V_cerebellum_VIIIa, 1, regional and tissue volume

IDP_T1_FAST_ROIs_R_cerebellum_VIIIa, 1, regional and tissue volume

IDP_T1_FAST_ROIs_L_cerebellum_VIIIb, 1, regional and tissue volume

IDP_T1_FAST_ROIs_V_cerebellum_VIIIb, 1, regional and tissue volume

IDP_T1_FAST_ROIs_R_cerebellum_VIIIb, 1, regional and tissue volume

IDP_T1_FAST_ROIs_L_cerebellum_IX, 1, regional and tissue volume

IDP_T1_FAST_ROIs_V_cerebellum_IX, 1, regional and tissue volume

IDP_T1_FAST_ROIs_R_cerebellum_IX, 1, regional and tissue volume

IDP_T1_FAST_ROIs_L_cerebellum_X, 1, regional and tissue volume

IDP_T1_FAST_ROIs_V_cerebellum_X, 1, regional and tissue volume

IDP_T1_FAST_ROIs_R_cerebellum_X, 1, regional and tissue volume

IDP_T1_VOL_OLFBULB_left, 1, regional and tissue volume

IDP_T1_VOL_OLFBULB_right, 1, regional and tissue volume

aseg_global_volume_BrainSeg, 1, regional and tissue volume

aseg_global_volume_BrainSegNotVent, 1, regional and tissue volume

aseg_global_volume_BrainSegNotVentSurf, 1, regional and tissue volume

aseg_global_volume_SubCortGray, 1, regional and tissue volume

aseg_global_volume_TotalGray, 1, regional and tissue volume

aseg_global_volume_SupraTentorial, 1, regional and tissue volume

aseg_global_volume_SupraTentorialNotVent, 1, regional and tissue volume

aseg_global_volume_EstimatedTotalIntraCranial, 1, regional and tissue volume

aseg_global_volume_VentricleChoroid, 1, regional and tissue volume

aseg_global_volume_3rd-Ventricle, 1, regional and tissue volume

aseg_global_volume_4th-Ventricle, 1, regional and tissue volume

aseg_global_volume_5th-Ventricle, 1, regional and tissue volume

aseg_global_volume_Brain-Stem, 1, regional and tissue volume

aseg_global_volume_CSF, 1, regional and tissue volume

aseg_global_volume_WM-hypointensities, 1, regional and tissue volume

aseg_global_volume_non-WM-hypointensities, 1, regional and tissue volume

aseg_global_volume_Optic-Chiasm, 1, regional and tissue volume

aseg_global_volume_CC-Posterior, 1, regional and tissue volume

aseg_global_volume_CC-Mid-Posterior, 1, regional and tissue volume

aseg_global_volume_CC-Central, 1, regional and tissue volume

aseg_global_volume_CC-Mid-Anterior, 1, regional and tissue volume

aseg_global_volume_CC-Anterior, 1, regional and tissue volume

aseg_global_volume-ratio_BrainSegVol-to-eTIV, 1, regional and tissue volume

aseg_global_volume-ratio_MaskVol-to-eTIV, 1, regional and tissue volume

aseg_lh_volume_Cortex, 1, regional and tissue volume

aseg_lh_volume_CerebralWhiteMatter, 1, regional and tissue volume

aseg_lh_volume_Lateral-Ventricle, 1, regional and tissue volume

aseg_lh_volume_Inf-Lat-Vent, 1, regional and tissue volume

aseg_lh_volume_Cerebellum-White-Matter, 1, regional and tissue volume

aseg_lh_volume_Cerebellum-Cortex, 1, regional and tissue volume

aseg_lh_volume_Thalamus-Proper, 1, regional and tissue volume

aseg_lh_volume_Caudate, 1, regional and tissue volume

aseg_lh_volume_Putamen, 1, regional and tissue volume

aseg_lh_volume_Pallidum, 1, regional and tissue volume

aseg_lh_volume_Hippocampus, 1, regional and tissue volume

aseg_lh_volume_Amygdala, 1, regional and tissue volume

aseg_lh_volume_Accumbens-area, 1, regional and tissue volume

aseg_lh_volume_VentralDC, 1, regional and tissue volume

aseg_lh_volume_vessel, 1, regional and tissue volume

aseg_lh_volume_choroid-plexus, 1, regional and tissue volume

aseg_lh_number_HolesBeforeFixing, 1, regional and tissue volume

aseg_rh_volume_Cortex, 1, regional and tissue volume

aseg_rh_volume_CerebralWhiteMatter, 1, regional and tissue volume

aseg_rh_volume_Lateral-Ventricle, 1, regional and tissue volume

aseg_rh_volume_Inf-Lat-Vent, 1, regional and tissue volume

aseg_rh_volume_Cerebellum-White-Matter, 1, regional and tissue volume

aseg_rh_volume_Cerebellum-Cortex, 1, regional and tissue volume

aseg_rh_volume_Thalamus-Proper, 1, regional and tissue volume

aseg_rh_volume_Caudate, 1, regional and tissue volume

aseg_rh_volume_Putamen, 1, regional and tissue volume

aseg_rh_volume_Pallidum, 1, regional and tissue volume

aseg_rh_volume_Hippocampus, 1, regional and tissue volume

aseg_rh_volume_Amygdala, 1, regional and tissue volume

aseg_rh_volume_Accumbens-area, 1, regional and tissue volume

aseg_rh_volume_VentralDC, 1, regional and tissue volume

aseg_rh_volume_vessel, 1, regional and tissue volume

aseg_rh_volume_choroid-plexus, 1, regional and tissue volume

aseg_rh_number_HolesBeforeFixing, 1, regional and tissue volume

AmygNuclei_lh_volume_Lateral-nucleus, 1, regional and tissue volume

AmygNuclei_lh_volume_Basal-nucleus, 1, regional and tissue volume

AmygNuclei_lh_volume_Accessory-Basal-nucleus, 1, regional and tissue volume

AmygNuclei_lh_volume_Anterior-amygdaloid-area-AAA, 1, regional and tissue volume

AmygNuclei_lh_volume_Central-nucleus, 1, regional and tissue volume

AmygNuclei_lh_volume_Medial-nucleus, 1, regional and tissue volume

AmygNuclei_lh_volume_Cortical-nucleus, 1, regional and tissue volume

AmygNuclei_lh_volume_Corticoamygdaloid-transitio, 1, regional and tissue volume

AmygNuclei_lh_volume_Paralaminar-nucleus, 1, regional and tissue volume

AmygNuclei_lh_volume_Whole-amygdala, 1, regional and tissue volume

AmygNuclei_rh_volume_Lateral-nucleus, 1, regional and tissue volume

AmygNuclei_rh_volume_Basal-nucleus, 1, regional and tissue volume

AmygNuclei_rh_volume_Accessory-Basal-nucleus, 1, regional and tissue volume

AmygNuclei_rh_volume_Anterior-amygdaloid-area-AAA, 1, regional and tissue volume

AmygNuclei_rh_volume_Central-nucleus, 1, regional and tissue volume

AmygNuclei_rh_volume_Medial-nucleus, 1, regional and tissue volume

AmygNuclei_rh_volume_Cortical-nucleus, 1, regional and tissue volume

AmygNuclei_rh_volume_Corticoamygdaloid-transitio, 1, regional and tissue volume

AmygNuclei_rh_volume_Paralaminar-nucleus, 1, regional and tissue volume

AmygNuclei_rh_volume_Whole-amygdala, 1, regional and tissue volume

HippSubfield_lh_volume_Hippocampal-tail, 1, regional and tissue volume

HippSubfield_lh_volume_subiculum-body, 1, regional and tissue volume

HippSubfield_lh_volume_CA1-body, 1, regional and tissue volume

HippSubfield_lh_volume_subiculum-head, 1, regional and tissue volume

HippSubfield_lh_volume_hippocampal-fissure, 1, regional and tissue volume

HippSubfield_lh_volume_presubiculum-head, 1, regional and tissue volume

HippSubfield_lh_volume_CA1-head, 1, regional and tissue volume

HippSubfield_lh_volume_presubiculum-body, 1, regional and tissue volume

HippSubfield_lh_volume_parasubiculum, 1, regional and tissue volume

HippSubfield_lh_volume_molecular-layer-HP-head, 1, regional and tissue volume

HippSubfield_lh_volume_molecular-layer-HP-body, 1, regional and tissue volume

HippSubfield_lh_volume_GC-ML-DG-head, 1, regional and tissue volume

HippSubfield_lh_volume_CA3-body, 1, regional and tissue volume

HippSubfield_lh_volume_GC-ML-DG-body, 1, regional and tissue volume

HippSubfield_lh_volume_CA4-head, 1, regional and tissue volume

HippSubfield_lh_volume_CA4-body, 1, regional and tissue volume

HippSubfield_lh_volume_fimbria, 1, regional and tissue volume

HippSubfield_lh_volume_CA3-head, 1, regional and tissue volume

HippSubfield_lh_volume_HATA, 1, regional and tissue volume

HippSubfield_lh_volume_Whole-hippocampal-body, 1, regional and tissue volume

HippSubfield_lh_volume_Whole-hippocampal-head, 1, regional and tissue volume

HippSubfield_lh_volume_Whole-hippocampus, 1, regional and tissue volume

HippSubfield_rh_volume_Hippocampal-tail, 1, regional and tissue volume

HippSubfield_rh_volume_subiculum-body, 1, regional and tissue volume

HippSubfield_rh_volume_CA1-body, 1, regional and tissue volume

HippSubfield_rh_volume_subiculum-head, 1, regional and tissue volume

HippSubfield_rh_volume_hippocampal-fissure, 1, regional and tissue volume

HippSubfield_rh_volume_presubiculum-head, 1, regional and tissue volume

HippSubfield_rh_volume_CA1-head, 1, regional and tissue volume

HippSubfield_rh_volume_presubiculum-body, 1, regional and tissue volume

HippSubfield_rh_volume_parasubiculum, 1, regional and tissue volume

HippSubfield_rh_volume_molecular-layer-HP-head, 1, regional and tissue volume

HippSubfield_rh_volume_molecular-layer-HP-body, 1, regional and tissue volume

HippSubfield_rh_volume_GC-ML-DG-head, 1, regional and tissue volume

HippSubfield_rh_volume_CA3-body, 1, regional and tissue volume

HippSubfield_rh_volume_GC-ML-DG-body, 1, regional and tissue volume

HippSubfield_rh_volume_CA4-head, 1, regional and tissue volume

HippSubfield_rh_volume_CA4-body, 1, regional and tissue volume

HippSubfield_rh_volume_fimbria, 1, regional and tissue volume

HippSubfield_rh_volume_CA3-head, 1, regional and tissue volume

HippSubfield_rh_volume_HATA, 1, regional and tissue volume

HippSubfield_rh_volume_Whole-hippocampal-body, 1, regional and tissue volume

HippSubfield_rh_volume_Whole-hippocampal-head, 1, regional and tissue volume

HippSubfield_rh_volume_Whole-hippocampus, 1, regional and tissue volume

ThalamNuclei_lh_volume_MGN, 1, regional and tissue volume

ThalamNuclei_lh_volume_LGN, 1, regional and tissue volume

ThalamNuclei_lh_volume_PuI, 1, regional and tissue volume

ThalamNuclei_lh_volume_PuM, 1, regional and tissue volume

ThalamNuclei_lh_volume_L-Sg, 1, regional and tissue volume

ThalamNuclei_lh_volume_VPL, 1, regional and tissue volume

ThalamNuclei_lh_volume_CM, 1, regional and tissue volume

ThalamNuclei_lh_volume_VLa, 1, regional and tissue volume

ThalamNuclei_lh_volume_PuA, 1, regional and tissue volume

ThalamNuclei_lh_volume_MDm, 1, regional and tissue volume

ThalamNuclei_lh_volume_Pf, 1, regional and tissue volume

ThalamNuclei_lh_volume_VAmc, 1, regional and tissue volume

ThalamNuclei_lh_volume_MDl, 1, regional and tissue volume

ThalamNuclei_lh_volume_CeM, 1, regional and tissue volume

ThalamNuclei_lh_volume_VA, 1, regional and tissue volume

ThalamNuclei_lh_volume_MV(Re), 1, regional and tissue volume

ThalamNuclei_lh_volume_VM, 1, regional and tissue volume

ThalamNuclei_lh_volume_CL, 1, regional and tissue volume

ThalamNuclei_lh_volume_PuL, 1, regional and tissue volume

ThalamNuclei_lh_volume_Pt, 1, regional and tissue volume

ThalamNuclei_lh_volume_AV, 1, regional and tissue volume

ThalamNuclei_lh_volume_Pc, 1, regional and tissue volume

ThalamNuclei_lh_volume_VLp, 1, regional and tissue volume

ThalamNuclei_lh_volume_LP, 1, regional and tissue volume

ThalamNuclei_rh_volume_LGN, 1, regional and tissue volume

ThalamNuclei_rh_volume_MGN, 1, regional and tissue volume

ThalamNuclei_rh_volume_PuI, 1, regional and tissue volume

ThalamNuclei_rh_volume_PuM, 1, regional and tissue volume

ThalamNuclei_rh_volume_L-Sg, 1, regional and tissue volume

ThalamNuclei_rh_volume_VPL, 1, regional and tissue volume

ThalamNuclei_rh_volume_CM, 1, regional and tissue volume

ThalamNuclei_rh_volume_VLa, 1, regional and tissue volume

ThalamNuclei_rh_volume_PuA, 1, regional and tissue volume

ThalamNuclei_rh_volume_MDm, 1, regional and tissue volume

ThalamNuclei_rh_volume_Pf, 1, regional and tissue volume

ThalamNuclei_rh_volume_VAmc, 1, regional and tissue volume

ThalamNuclei_rh_volume_MDl, 1, regional and tissue volume

ThalamNuclei_rh_volume_VA, 1, regional and tissue volume

ThalamNuclei_rh_volume_MV(Re), 1, regional and tissue volume

ThalamNuclei_rh_volume_CeM, 1, regional and tissue volume

ThalamNuclei_rh_volume_VM, 1, regional and tissue volume

ThalamNuclei_rh_volume_PuL, 1, regional and tissue volume

ThalamNuclei_rh_volume_CL, 1, regional and tissue volume

ThalamNuclei_rh_volume_VLp, 1, regional and tissue volume

ThalamNuclei_rh_volume_Pc, 1, regional and tissue volume

ThalamNuclei_rh_volume_Pt, 1, regional and tissue volume

ThalamNuclei_rh_volume_AV, 1, regional and tissue volume

ThalamNuclei_rh_volume_LP, 1, regional and tissue volume

ThalamNuclei_lh_volume_LD, 1, regional and tissue volume

ThalamNuclei_rh_volume_LD, 1, regional and tissue volume

ThalamNuclei_lh_volume_Whole-thalamus, 1, regional and tissue volume

ThalamNuclei_rh_volume_Whole-thalamus, 1, regional and tissue volume

Brainstem_global_volume_Medulla, 1, regional and tissue volume

Brainstem_global_volume_Pons, 1, regional and tissue volume

Brainstem_global_volume_SCP, 1, regional and tissue volume

Brainstem_global_volume_Midbrain, 1, regional and tissue volume

Brainstem_global_volume_Whole-brainstem, 1, regional and tissue volume

aparc-Desikan_lh_volume_bankssts, 1, regional and tissue volume

aparc-Desikan_lh_volume_caudalanteriorcingulate, 1, regional and tissue volume

aparc-Desikan_lh_volume_caudalmiddlefrontal, 1, regional and tissue volume

aparc-Desikan_lh_volume_cuneus, 1, regional and tissue volume

aparc-Desikan_lh_volume_entorhinal, 1, regional and tissue volume

aparc-Desikan_lh_volume_fusiform, 1, regional and tissue volume

aparc-Desikan_lh_volume_inferiorparietal, 1, regional and tissue volume

aparc-Desikan_lh_volume_inferiortemporal, 1, regional and tissue volume

aparc-Desikan_lh_volume_isthmuscingulate, 1, regional and tissue volume

aparc-Desikan_lh_volume_lateraloccipital, 1, regional and tissue volume

aparc-Desikan_lh_volume_lateralorbitofrontal, 1, regional and tissue volume

aparc-Desikan_lh_volume_lingual, 1, regional and tissue volume

aparc-Desikan_lh_volume_medialorbitofrontal, 1, regional and tissue volume

aparc-Desikan_lh_volume_middletemporal, 1, regional and tissue volume

aparc-Desikan_lh_volume_parahippocampal, 1, regional and tissue volume

aparc-Desikan_lh_volume_paracentral, 1, regional and tissue volume

aparc-Desikan_lh_volume_parsopercularis, 1, regional and tissue volume

aparc-Desikan_lh_volume_parsorbitalis, 1, regional and tissue volume

aparc-Desikan_lh_volume_parstriangularis, 1, regional and tissue volume

aparc-Desikan_lh_volume_pericalcarine, 1, regional and tissue volume

aparc-Desikan_lh_volume_postcentral, 1, regional and tissue volume

aparc-Desikan_lh_volume_posteriorcingulate, 1, regional and tissue volume

aparc-Desikan_lh_volume_precentral, 1, regional and tissue volume

aparc-Desikan_lh_volume_precuneus, 1, regional and tissue volume

aparc-Desikan_lh_volume_rostralanteriorcingulate, 1, regional and tissue volume

aparc-Desikan_lh_volume_rostralmiddlefrontal, 1, regional and tissue volume

aparc-Desikan_lh_volume_superiorfrontal, 1, regional and tissue volume

aparc-Desikan_lh_volume_superiorparietal, 1, regional and tissue volume

aparc-Desikan_lh_volume_superiortemporal, 1, regional and tissue volume

aparc-Desikan_lh_volume_supramarginal, 1, regional and tissue volume

aparc-Desikan_lh_volume_frontalpole, 1, regional and tissue volume

aparc-Desikan_lh_volume_transversetemporal, 1, regional and tissue volume

aparc-Desikan_lh_volume_insula, 1, regional and tissue volume

aparc-Desikan_rh_volume_bankssts, 1, regional and tissue volume

aparc-Desikan_rh_volume_caudalanteriorcingulate, 1, regional and tissue volume

aparc-Desikan_rh_volume_caudalmiddlefrontal, 1, regional and tissue volume

aparc-Desikan_rh_volume_cuneus, 1, regional and tissue volume

aparc-Desikan_rh_volume_entorhinal, 1, regional and tissue volume

aparc-Desikan_rh_volume_fusiform, 1, regional and tissue volume

aparc-Desikan_rh_volume_inferiorparietal, 1, regional and tissue volume

aparc-Desikan_rh_volume_inferiortemporal, 1, regional and tissue volume

aparc-Desikan_rh_volume_isthmuscingulate, 1, regional and tissue volume

aparc-Desikan_rh_volume_lateraloccipital, 1, regional and tissue volume

aparc-Desikan_rh_volume_lateralorbitofrontal, 1, regional and tissue volume

aparc-Desikan_rh_volume_lingual, 1, regional and tissue volume

aparc-Desikan_rh_volume_medialorbitofrontal, 1, regional and tissue volume

aparc-Desikan_rh_volume_middletemporal, 1, regional and tissue volume

aparc-Desikan_rh_volume_parahippocampal, 1, regional and tissue volume

aparc-Desikan_rh_volume_paracentral, 1, regional and tissue volume

aparc-Desikan_rh_volume_parsopercularis, 1, regional and tissue volume

aparc-Desikan_rh_volume_parsorbitalis, 1, regional and tissue volume

aparc-Desikan_rh_volume_parstriangularis, 1, regional and tissue volume

aparc-Desikan_rh_volume_pericalcarine, 1, regional and tissue volume

aparc-Desikan_rh_volume_postcentral, 1, regional and tissue volume

aparc-Desikan_rh_volume_posteriorcingulate, 1, regional and tissue volume

aparc-Desikan_rh_volume_precentral, 1, regional and tissue volume

aparc-Desikan_rh_volume_precuneus, 1, regional and tissue volume

aparc-Desikan_rh_volume_rostralanteriorcingulate, 1, regional and tissue volume

aparc-Desikan_rh_volume_rostralmiddlefrontal, 1, regional and tissue volume

aparc-Desikan_rh_volume_superiorfrontal, 1, regional and tissue volume

aparc-Desikan_rh_volume_superiorparietal, 1, regional and tissue volume

aparc-Desikan_rh_volume_superiortemporal, 1, regional and tissue volume

aparc-Desikan_rh_volume_supramarginal, 1, regional and tissue volume

aparc-Desikan_rh_volume_frontalpole, 1, regional and tissue volume

aparc-Desikan_rh_volume_transversetemporal, 1, regional and tissue volume

aparc-Desikan_rh_volume_insula, 1, regional and tissue volume

BA-exvivo_lh_volume_BA1, 1, regional and tissue volume

BA-exvivo_lh_volume_BA2, 1, regional and tissue volume

BA-exvivo_lh_volume_BA3a, 1, regional and tissue volume

BA-exvivo_lh_volume_BA3b, 1, regional and tissue volume

BA-exvivo_lh_volume_BA4a, 1, regional and tissue volume

BA-exvivo_lh_volume_BA4p, 1, regional and tissue volume

BA-exvivo_lh_volume_BA6, 1, regional and tissue volume

BA-exvivo_lh_volume_BA44, 1, regional and tissue volume

BA-exvivo_lh_volume_BA45, 1, regional and tissue volume

BA-exvivo_lh_volume_V1, 1, regional and tissue volume

BA-exvivo_lh_volume_V2, 1, regional and tissue volume

BA-exvivo_lh_volume_MT, 1, regional and tissue volume

BA-exvivo_lh_volume_perirhinal, 1, regional and tissue volume

BA-exvivo_lh_volume_entorhinal, 1, regional and tissue volume

BA-exvivo_rh_volume_BA1, 1, regional and tissue volume

BA-exvivo_rh_volume_BA2, 1, regional and tissue volume

BA-exvivo_rh_volume_BA3a, 1, regional and tissue volume

BA-exvivo_rh_volume_BA3b, 1, regional and tissue volume

BA-exvivo_rh_volume_BA4a, 1, regional and tissue volume

BA-exvivo_rh_volume_BA4p, 1, regional and tissue volume

BA-exvivo_rh_volume_BA6, 1, regional and tissue volume

BA-exvivo_rh_volume_BA44, 1, regional and tissue volume

BA-exvivo_rh_volume_BA45, 1, regional and tissue volume

BA-exvivo_rh_volume_V1, 1, regional and tissue volume

BA-exvivo_rh_volume_V2, 1, regional and tissue volume

BA-exvivo_rh_volume_MT, 1, regional and tissue volume

BA-exvivo_rh_volume_perirhinal, 1, regional and tissue volume

BA-exvivo_rh_volume_entorhinal, 1, regional and tissue volume

aparc-DKTatlas_lh_volume_caudalanteriorcingulate, 1, regional and tissue volume

aparc-DKTatlas_lh_volume_caudalmiddlefrontal, 1, regional and tissue volume

aparc-DKTatlas_lh_volume_cuneus, 1, regional and tissue volume

aparc-DKTatlas_lh_volume_entorhinal, 1, regional and tissue volume

aparc-DKTatlas_lh_volume_fusiform, 1, regional and tissue volume

aparc-DKTatlas_lh_volume_inferiorparietal, 1, regional and tissue volume

aparc-DKTatlas_lh_volume_inferiortemporal, 1, regional and tissue volume

aparc-DKTatlas_lh_volume_isthmuscingulate, 1, regional and tissue volume

aparc-DKTatlas_lh_volume_lateraloccipital, 1, regional and tissue volume

aparc-DKTatlas_lh_volume_lateralorbitofrontal, 1, regional and tissue volume

aparc-DKTatlas_lh_volume_lingual, 1, regional and tissue volume

aparc-DKTatlas_lh_volume_medialorbitofrontal, 1, regional and tissue volume

aparc-DKTatlas_lh_volume_middletemporal, 1, regional and tissue volume

aparc-DKTatlas_lh_volume_parahippocampal, 1, regional and tissue volume

aparc-DKTatlas_lh_volume_paracentral, 1, regional and tissue volume

aparc-DKTatlas_lh_volume_parsopercularis, 1, regional and tissue volume

aparc-DKTatlas_lh_volume_parsorbitalis, 1, regional and tissue volume

aparc-DKTatlas_lh_volume_parstriangularis, 1, regional and tissue volume

aparc-DKTatlas_lh_volume_pericalcarine, 1, regional and tissue volume

aparc-DKTatlas_lh_volume_postcentral, 1, regional and tissue volume

aparc-DKTatlas_lh_volume_posteriorcingulate, 1, regional and tissue volume

aparc-DKTatlas_lh_volume_precentral, 1, regional and tissue volume

aparc-DKTatlas_lh_volume_precuneus, 1, regional and tissue volume

aparc-DKTatlas_lh_volume_rostralanteriorcingulate, 1, regional and tissue volume

aparc-DKTatlas_lh_volume_rostralmiddlefrontal, 1, regional and tissue volume

aparc-DKTatlas_lh_volume_superiorfrontal, 1, regional and tissue volume

aparc-DKTatlas_lh_volume_superiorparietal, 1, regional and tissue volume

aparc-DKTatlas_lh_volume_superiortemporal, 1, regional and tissue volume

aparc-DKTatlas_lh_volume_supramarginal, 1, regional and tissue volume

aparc-DKTatlas_lh_volume_transversetemporal, 1, regional and tissue volume

aparc-DKTatlas_lh_volume_insula, 1, regional and tissue volume

aparc-DKTatlas_rh_volume_caudalanteriorcingulate, 1, regional and tissue volume

aparc-DKTatlas_rh_volume_caudalmiddlefrontal, 1, regional and tissue volume

aparc-DKTatlas_rh_volume_cuneus, 1, regional and tissue volume

aparc-DKTatlas_rh_volume_entorhinal, 1, regional and tissue volume

aparc-DKTatlas_rh_volume_fusiform, 1, regional and tissue volume

aparc-DKTatlas_rh_volume_inferiorparietal, 1, regional and tissue volume

aparc-DKTatlas_rh_volume_inferiortemporal, 1, regional and tissue volume

aparc-DKTatlas_rh_volume_isthmuscingulate, 1, regional and tissue volume

aparc-DKTatlas_rh_volume_lateraloccipital, 1, regional and tissue volume

aparc-DKTatlas_rh_volume_lateralorbitofrontal, 1, regional and tissue volume

aparc-DKTatlas_rh_volume_lingual, 1, regional and tissue volume

aparc-DKTatlas_rh_volume_medialorbitofrontal, 1, regional and tissue volume

aparc-DKTatlas_rh_volume_middletemporal, 1, regional and tissue volume

aparc-DKTatlas_rh_volume_parahippocampal, 1, regional and tissue volume

aparc-DKTatlas_rh_volume_paracentral, 1, regional and tissue volume

aparc-DKTatlas_rh_volume_parsopercularis, 1, regional and tissue volume

aparc-DKTatlas_rh_volume_parsorbitalis, 1, regional and tissue volume

aparc-DKTatlas_rh_volume_parstriangularis, 1, regional and tissue volume

aparc-DKTatlas_rh_volume_pericalcarine, 1, regional and tissue volume

aparc-DKTatlas_rh_volume_postcentral, 1, regional and tissue volume

aparc-DKTatlas_rh_volume_posteriorcingulate, 1, regional and tissue volume

aparc-DKTatlas_rh_volume_precentral, 1, regional and tissue volume

aparc-DKTatlas_rh_volume_precuneus, 1, regional and tissue volume

aparc-DKTatlas_rh_volume_rostralanteriorcingulate, 1, regional and tissue volume

aparc-DKTatlas_rh_volume_rostralmiddlefrontal, 1, regional and tissue volume

aparc-DKTatlas_rh_volume_superiorfrontal, 1, regional and tissue volume

aparc-DKTatlas_rh_volume_superiorparietal, 1, regional and tissue volume

aparc-DKTatlas_rh_volume_superiortemporal, 1, regional and tissue volume

aparc-DKTatlas_rh_volume_supramarginal, 1, regional and tissue volume

aparc-DKTatlas_rh_volume_transversetemporal, 1, regional and tissue volume

aparc-DKTatlas_rh_volume_insula, 1, regional and tissue volume

aparc-a2009s_lh_volume_G+S-frontomargin, 1, regional and tissue volume

aparc-a2009s_lh_volume_G+S-occipital-inf, 1, regional and tissue volume

aparc-a2009s_lh_volume_G+S-paracentral, 1, regional and tissue volume

aparc-a2009s_lh_volume_G+S-subcentral, 1, regional and tissue volume

aparc-a2009s_lh_volume_G+S-transv-frontopol, 1, regional and tissue volume

aparc-a2009s_lh_volume_G+S-cingul-Ant, 1, regional and tissue volume

aparc-a2009s_lh_volume_G+S-cingul-Mid-Ant, 1, regional and tissue volume

aparc-a2009s_lh_volume_G+S-cingul-Mid-Post, 1, regional and tissue volume

aparc-a2009s_lh_volume_G-cingul-Post-dorsal, 1, regional and tissue volume

aparc-a2009s_lh_volume_G-cingul-Post-ventral, 1, regional and tissue volume

aparc-a2009s_lh_volume_G-cuneus, 1, regional and tissue volume

aparc-a2009s_lh_volume_G-front-inf-Opercular, 1, regional and tissue volume

aparc-a2009s_lh_volume_G-front-inf-Orbital, 1, regional and tissue volume

aparc-a2009s_lh_volume_G-front-inf-Triangul, 1, regional and tissue volume

aparc-a2009s_lh_volume_G-front-middle, 1, regional and tissue volume

aparc-a2009s_lh_volume_G-front-sup, 1, regional and tissue volume

aparc-a2009s_lh_volume_G-Ins-lg+S-cent-ins, 1, regional and tissue volume

aparc-a2009s_lh_volume_G-insular-short, 1, regional and tissue volume

aparc-a2009s_lh_volume_G-occipital-middle, 1, regional and tissue volume

aparc-a2009s_lh_volume_G-occipital-sup, 1, regional and tissue volume

aparc-a2009s_lh_volume_G-oc-temp-lat-fusifor, 1, regional and tissue volume

aparc-a2009s_lh_volume_G-oc-temp-med-Lingual, 1, regional and tissue volume

aparc-a2009s_lh_volume_G-oc-temp-med-Parahip, 1, regional and tissue volume

aparc-a2009s_lh_volume_G-orbital, 1, regional and tissue volume

aparc-a2009s_lh_volume_G-pariet-inf-Angular, 1, regional and tissue volume

aparc-a2009s_lh_volume_G-pariet-inf-Supramar, 1, regional and tissue volume

aparc-a2009s_lh_volume_G-parietal-sup, 1, regional and tissue volume

aparc-a2009s_lh_volume_G-postcentral, 1, regional and tissue volume

aparc-a2009s_lh_volume_G-precentral, 1, regional and tissue volume

aparc-a2009s_lh_volume_G-precuneus, 1, regional and tissue volume

aparc-a2009s_lh_volume_G-rectus, 1, regional and tissue volume

aparc-a2009s_lh_volume_G-subcallosal, 1, regional and tissue volume

aparc-a2009s_lh_volume_G-temp-sup-G-T-transv, 1, regional and tissue volume

aparc-a2009s_lh_volume_G-temp-sup-Lateral, 1, regional and tissue volume

aparc-a2009s_lh_volume_G-temp-sup-Plan-polar, 1, regional and tissue volume

aparc-a2009s_lh_volume_G-temp-sup-Plan-tempo, 1, regional and tissue volume

aparc-a2009s_lh_volume_G-temporal-inf, 1, regional and tissue volume

aparc-a2009s_lh_volume_G-temporal-middle, 1, regional and tissue volume

aparc-a2009s_lh_volume_Lat-Fis-ant-Horizont, 1, regional and tissue volume

aparc-a2009s_lh_volume_Lat-Fis-ant-Vertical, 1, regional and tissue volume

aparc-a2009s_lh_volume_Lat-Fis-post, 1, regional and tissue volume

aparc-a2009s_lh_volume_Pole-occipital, 1, regional and tissue volume

aparc-a2009s_lh_volume_Pole-temporal, 1, regional and tissue volume

aparc-a2009s_lh_volume_S-calcarine, 1, regional and tissue volume

aparc-a2009s_lh_volume_S-central, 1, regional and tissue volume

aparc-a2009s_lh_volume_S-cingul-Marginalis, 1, regional and tissue volume

aparc-a2009s_lh_volume_S-circular-insula-ant, 1, regional and tissue volume

aparc-a2009s_lh_volume_S-circular-insula-inf, 1, regional and tissue volume

aparc-a2009s_lh_volume_S-circular-insula-sup, 1, regional and tissue volume

aparc-a2009s_lh_volume_S-collat-transv-ant, 1, regional and tissue volume

aparc-a2009s_lh_volume_S-collat-transv-post, 1, regional and tissue volume

aparc-a2009s_lh_volume_S-front-inf, 1, regional and tissue volume

aparc-a2009s_lh_volume_S-front-middle, 1, regional and tissue volume

aparc-a2009s_lh_volume_S-front-sup, 1, regional and tissue volume

aparc-a2009s_lh_volume_S-interm-prim-Jensen, 1, regional and tissue volume

aparc-a2009s_lh_volume_S-intrapariet+P-trans, 1, regional and tissue volume

aparc-a2009s_lh_volume_S-oc-middle+Lunatus, 1, regional and tissue volume

aparc-a2009s_lh_volume_S-oc-sup+transversal, 1, regional and tissue volume

aparc-a2009s_lh_volume_S-occipital-ant, 1, regional and tissue volume

aparc-a2009s_lh_volume_S-oc-temp-lat, 1, regional and tissue volume

aparc-a2009s_lh_volume_S-oc-temp-med+Lingual, 1, regional and tissue volume

aparc-a2009s_lh_volume_S-orbital-lateral, 1, regional and tissue volume

aparc-a2009s_lh_volume_S-orbital-med-olfact, 1, regional and tissue volume

aparc-a2009s_lh_volume_S-orbital-H-Shaped, 1, regional and tissue volume

aparc-a2009s_lh_volume_S-parieto-occipital, 1, regional and tissue volume

aparc-a2009s_lh_volume_S-pericallosal, 1, regional and tissue volume

aparc-a2009s_lh_volume_S-postcentral, 1, regional and tissue volume

aparc-a2009s_lh_volume_S-precentral-inf-part, 1, regional and tissue volume

aparc-a2009s_lh_volume_S-precentral-sup-part, 1, regional and tissue volume

aparc-a2009s_lh_volume_S-suborbital, 1, regional and tissue volume

aparc-a2009s_lh_volume_S-subparietal, 1, regional and tissue volume

aparc-a2009s_lh_volume_S-temporal-inf, 1, regional and tissue volume

aparc-a2009s_lh_volume_S-temporal-sup, 1, regional and tissue volume

aparc-a2009s_lh_volume_S-temporal-transverse, 1, regional and tissue volume

aparc-a2009s_rh_volume_G+S-frontomargin, 1, regional and tissue volume

aparc-a2009s_rh_volume_G+S-occipital-inf, 1, regional and tissue volume

aparc-a2009s_rh_volume_G+S-paracentral, 1, regional and tissue volume

aparc-a2009s_rh_volume_G+S-subcentral, 1, regional and tissue volume

aparc-a2009s_rh_volume_G+S-transv-frontopol, 1, regional and tissue volume

aparc-a2009s_rh_volume_G+S-cingul-Ant, 1, regional and tissue volume

aparc-a2009s_rh_volume_G+S-cingul-Mid-Ant, 1, regional and tissue volume

aparc-a2009s_rh_volume_G+S-cingul-Mid-Post, 1, regional and tissue volume

aparc-a2009s_rh_volume_G-cingul-Post-dorsal, 1, regional and tissue volume

aparc-a2009s_rh_volume_G-cingul-Post-ventral, 1, regional and tissue volume

aparc-a2009s_rh_volume_G-cuneus, 1, regional and tissue volume

aparc-a2009s_rh_volume_G-front-inf-Opercular, 1, regional and tissue volume

aparc-a2009s_rh_volume_G-front-inf-Orbital, 1, regional and tissue volume

aparc-a2009s_rh_volume_G-front-inf-Triangul, 1, regional and tissue volume

aparc-a2009s_rh_volume_G-front-middle, 1, regional and tissue volume

aparc-a2009s_rh_volume_G-front-sup, 1, regional and tissue volume

aparc-a2009s_rh_volume_G-Ins-lg+S-cent-ins, 1, regional and tissue volume

aparc-a2009s_rh_volume_G-insular-short, 1, regional and tissue volume

aparc-a2009s_rh_volume_G-occipital-middle, 1, regional and tissue volume

aparc-a2009s_rh_volume_G-occipital-sup, 1, regional and tissue volume

aparc-a2009s_rh_volume_G-oc-temp-lat-fusifor, 1, regional and tissue volume

aparc-a2009s_rh_volume_G-oc-temp-med-Lingual, 1, regional and tissue volume

aparc-a2009s_rh_volume_G-oc-temp-med-Parahip, 1, regional and tissue volume

aparc-a2009s_rh_volume_G-orbital, 1, regional and tissue volume

aparc-a2009s_rh_volume_G-pariet-inf-Angular, 1, regional and tissue volume

aparc-a2009s_rh_volume_G-pariet-inf-Supramar, 1, regional and tissue volume

aparc-a2009s_rh_volume_G-parietal-sup, 1, regional and tissue volume

aparc-a2009s_rh_volume_G-postcentral, 1, regional and tissue volume

aparc-a2009s_rh_volume_G-precentral, 1, regional and tissue volume

aparc-a2009s_rh_volume_G-precuneus, 1, regional and tissue volume

aparc-a2009s_rh_volume_G-rectus, 1, regional and tissue volume

aparc-a2009s_rh_volume_G-subcallosal, 1, regional and tissue volume

aparc-a2009s_rh_volume_G-temp-sup-G-T-transv, 1, regional and tissue volume

aparc-a2009s_rh_volume_G-temp-sup-Lateral, 1, regional and tissue volume

aparc-a2009s_rh_volume_G-temp-sup-Plan-polar, 1, regional and tissue volume

aparc-a2009s_rh_volume_G-temp-sup-Plan-tempo, 1, regional and tissue volume

aparc-a2009s_rh_volume_G-temporal-inf, 1, regional and tissue volume

aparc-a2009s_rh_volume_G-temporal-middle, 1, regional and tissue volume

aparc-a2009s_rh_volume_Lat-Fis-ant-Horizont, 1, regional and tissue volume

aparc-a2009s_rh_volume_Lat-Fis-ant-Vertical, 1, regional and tissue volume

aparc-a2009s_rh_volume_Lat-Fis-post, 1, regional and tissue volume

aparc-a2009s_rh_volume_Pole-occipital, 1, regional and tissue volume

aparc-a2009s_rh_volume_Pole-temporal, 1, regional and tissue volume

aparc-a2009s_rh_volume_S-calcarine, 1, regional and tissue volume

aparc-a2009s_rh_volume_S-central, 1, regional and tissue volume

aparc-a2009s_rh_volume_S-cingul-Marginalis, 1, regional and tissue volume

aparc-a2009s_rh_volume_S-circular-insula-ant, 1, regional and tissue volume

aparc-a2009s_rh_volume_S-circular-insula-inf, 1, regional and tissue volume

aparc-a2009s_rh_volume_S-circular-insula-sup, 1, regional and tissue volume

aparc-a2009s_rh_volume_S-collat-transv-ant, 1, regional and tissue volume

aparc-a2009s_rh_volume_S-collat-transv-post, 1, regional and tissue volume

aparc-a2009s_rh_volume_S-front-inf, 1, regional and tissue volume

aparc-a2009s_rh_volume_S-front-middle, 1, regional and tissue volume

aparc-a2009s_rh_volume_S-front-sup, 1, regional and tissue volume

aparc-a2009s_rh_volume_S-interm-prim-Jensen, 1, regional and tissue volume

aparc-a2009s_rh_volume_S-intrapariet+P-trans, 1, regional and tissue volume

aparc-a2009s_rh_volume_S-oc-middle+Lunatus, 1, regional and tissue volume

aparc-a2009s_rh_volume_S-oc-sup+transversal, 1, regional and tissue volume

aparc-a2009s_rh_volume_S-occipital-ant, 1, regional and tissue volume

aparc-a2009s_rh_volume_S-oc-temp-lat, 1, regional and tissue volume

aparc-a2009s_rh_volume_S-oc-temp-med+Lingual, 1, regional and tissue volume

aparc-a2009s_rh_volume_S-orbital-lateral, 1, regional and tissue volume

aparc-a2009s_rh_volume_S-orbital-med-olfact, 1, regional and tissue volume

aparc-a2009s_rh_volume_S-orbital-H-Shaped, 1, regional and tissue volume

aparc-a2009s_rh_volume_S-parieto-occipital, 1, regional and tissue volume

aparc-a2009s_rh_volume_S-pericallosal, 1, regional and tissue volume

aparc-a2009s_rh_volume_S-postcentral, 1, regional and tissue volume

aparc-a2009s_rh_volume_S-precentral-inf-part, 1, regional and tissue volume

aparc-a2009s_rh_volume_S-precentral-sup-part, 1, regional and tissue volume

aparc-a2009s_rh_volume_S-suborbital, 1, regional and tissue volume

aparc-a2009s_rh_volume_S-subparietal, 1, regional and tissue volume

aparc-a2009s_rh_volume_S-temporal-inf, 1, regional and tissue volume

aparc-a2009s_rh_volume_S-temporal-sup, 1, regional and tissue volume

aparc-a2009s_rh_volume_S-temporal-transverse, 1, regional and tissue volume

aparc-Desikan_lh_area_TotalSurface, 2, cortical area

aparc-Desikan_lh_area_bankssts, 2, cortical area

aparc-Desikan_lh_area_caudalanteriorcingulate, 2, cortical area

aparc-Desikan_lh_area_caudalmiddlefrontal, 2, cortical area

aparc-Desikan_lh_area_cuneus, 2, cortical area

aparc-Desikan_lh_area_entorhinal, 2, cortical area

aparc-Desikan_lh_area_fusiform, 2, cortical area

aparc-Desikan_lh_area_inferiorparietal, 2, cortical area

aparc-Desikan_lh_area_inferiortemporal, 2, cortical area

aparc-Desikan_lh_area_isthmuscingulate, 2, cortical area

aparc-Desikan_lh_area_lateraloccipital, 2, cortical area

aparc-Desikan_lh_area_lateralorbitofrontal, 2, cortical area

aparc-Desikan_lh_area_lingual, 2, cortical area

aparc-Desikan_lh_area_medialorbitofrontal, 2, cortical area

aparc-Desikan_lh_area_middletemporal, 2, cortical area

aparc-Desikan_lh_area_parahippocampal, 2, cortical area

aparc-Desikan_lh_area_paracentral, 2, cortical area

aparc-Desikan_lh_area_parsopercularis, 2, cortical area

aparc-Desikan_lh_area_parsorbitalis, 2, cortical area

aparc-Desikan_lh_area_parstriangularis, 2, cortical area

aparc-Desikan_lh_area_pericalcarine, 2, cortical area

aparc-Desikan_lh_area_postcentral, 2, cortical area

aparc-Desikan_lh_area_posteriorcingulate, 2, cortical area

aparc-Desikan_lh_area_precentral, 2, cortical area

aparc-Desikan_lh_area_precuneus, 2, cortical area

aparc-Desikan_lh_area_rostralanteriorcingulate, 2, cortical area

aparc-Desikan_lh_area_rostralmiddlefrontal, 2, cortical area

aparc-Desikan_lh_area_superiorfrontal, 2, cortical area

aparc-Desikan_lh_area_superiorparietal, 2, cortical area

aparc-Desikan_lh_area_superiortemporal, 2, cortical area

aparc-Desikan_lh_area_supramarginal, 2, cortical area

aparc-Desikan_lh_area_frontalpole, 2, cortical area

aparc-Desikan_lh_area_transversetemporal, 2, cortical area

aparc-Desikan_lh_area_insula, 2, cortical area

aparc-Desikan_rh_area_TotalSurface, 2, cortical area

aparc-Desikan_rh_area_bankssts, 2, cortical area

aparc-Desikan_rh_area_caudalanteriorcingulate, 2, cortical area

aparc-Desikan_rh_area_caudalmiddlefrontal, 2, cortical area

aparc-Desikan_rh_area_cuneus, 2, cortical area

aparc-Desikan_rh_area_entorhinal, 2, cortical area

aparc-Desikan_rh_area_fusiform, 2, cortical area

aparc-Desikan_rh_area_inferiorparietal, 2, cortical area

aparc-Desikan_rh_area_inferiortemporal, 2, cortical area

aparc-Desikan_rh_area_isthmuscingulate, 2, cortical area

aparc-Desikan_rh_area_lateraloccipital, 2, cortical area

aparc-Desikan_rh_area_lateralorbitofrontal, 2, cortical area

aparc-Desikan_rh_area_lingual, 2, cortical area

aparc-Desikan_rh_area_medialorbitofrontal, 2, cortical area

aparc-Desikan_rh_area_middletemporal, 2, cortical area

aparc-Desikan_rh_area_parahippocampal, 2, cortical area

aparc-Desikan_rh_area_paracentral, 2, cortical area

aparc-Desikan_rh_area_parsopercularis, 2, cortical area

aparc-Desikan_rh_area_parsorbitalis, 2, cortical area

aparc-Desikan_rh_area_parstriangularis, 2, cortical area

aparc-Desikan_rh_area_pericalcarine, 2, cortical area

aparc-Desikan_rh_area_postcentral, 2, cortical area

aparc-Desikan_rh_area_posteriorcingulate, 2, cortical area

aparc-Desikan_rh_area_precentral, 2, cortical area

aparc-Desikan_rh_area_precuneus, 2, cortical area

aparc-Desikan_rh_area_rostralanteriorcingulate, 2, cortical area

aparc-Desikan_rh_area_rostralmiddlefrontal, 2, cortical area

aparc-Desikan_rh_area_superiorfrontal, 2, cortical area

aparc-Desikan_rh_area_superiorparietal, 2, cortical area

aparc-Desikan_rh_area_superiortemporal, 2, cortical area

aparc-Desikan_rh_area_supramarginal, 2, cortical area

aparc-Desikan_rh_area_frontalpole, 2, cortical area

aparc-Desikan_rh_area_transversetemporal, 2, cortical area

aparc-Desikan_rh_area_insula, 2, cortical area

aparc-pial_lh_area_TotalSurface, 2, cortical area

aparc-pial_lh_area_bankssts, 2, cortical area

aparc-pial_lh_area_caudalanteriorcingulate, 2, cortical area

aparc-pial_lh_area_caudalmiddlefrontal, 2, cortical area

aparc-pial_lh_area_cuneus, 2, cortical area

aparc-pial_lh_area_entorhinal, 2, cortical area

aparc-pial_lh_area_fusiform, 2, cortical area

aparc-pial_lh_area_inferiorparietal, 2, cortical area

aparc-pial_lh_area_inferiortemporal, 2, cortical area

aparc-pial_lh_area_isthmuscingulate, 2, cortical area

aparc-pial_lh_area_lateraloccipital, 2, cortical area

aparc-pial_lh_area_lateralorbitofrontal, 2, cortical area

aparc-pial_lh_area_lingual, 2, cortical area

aparc-pial_lh_area_medialorbitofrontal, 2, cortical area

aparc-pial_lh_area_middletemporal, 2, cortical area

aparc-pial_lh_area_parahippocampal, 2, cortical area

aparc-pial_lh_area_paracentral, 2, cortical area

aparc-pial_lh_area_parsopercularis, 2, cortical area

aparc-pial_lh_area_parsorbitalis, 2, cortical area

aparc-pial_lh_area_parstriangularis, 2, cortical area

aparc-pial_lh_area_pericalcarine, 2, cortical area

aparc-pial_lh_area_postcentral, 2, cortical area

aparc-pial_lh_area_posteriorcingulate, 2, cortical area

aparc-pial_lh_area_precentral, 2, cortical area

aparc-pial_lh_area_precuneus, 2, cortical area

aparc-pial_lh_area_rostralanteriorcingulate, 2, cortical area

aparc-pial_lh_area_rostralmiddlefrontal, 2, cortical area

aparc-pial_lh_area_superiorfrontal, 2, cortical area

aparc-pial_lh_area_superiorparietal, 2, cortical area

aparc-pial_lh_area_superiortemporal, 2, cortical area

aparc-pial_lh_area_supramarginal, 2, cortical area

aparc-pial_lh_area_frontalpole, 2, cortical area

aparc-pial_lh_area_transversetemporal, 2, cortical area

aparc-pial_rh_area_TotalSurface, 2, cortical area

aparc-pial_rh_area_bankssts, 2, cortical area

aparc-pial_rh_area_caudalanteriorcingulate, 2, cortical area

aparc-pial_rh_area_caudalmiddlefrontal, 2, cortical area

aparc-pial_rh_area_cuneus, 2, cortical area

aparc-pial_rh_area_entorhinal, 2, cortical area

aparc-pial_rh_area_fusiform, 2, cortical area

aparc-pial_rh_area_inferiorparietal, 2, cortical area

aparc-pial_rh_area_inferiortemporal, 2, cortical area

aparc-pial_rh_area_isthmuscingulate, 2, cortical area

aparc-pial_rh_area_lateraloccipital, 2, cortical area

aparc-pial_rh_area_lateralorbitofrontal, 2, cortical area

aparc-pial_rh_area_lingual, 2, cortical area

aparc-pial_rh_area_medialorbitofrontal, 2, cortical area

aparc-pial_rh_area_middletemporal, 2, cortical area

aparc-pial_rh_area_parahippocampal, 2, cortical area

aparc-pial_rh_area_paracentral, 2, cortical area

aparc-pial_rh_area_parsopercularis, 2, cortical area

aparc-pial_rh_area_parsorbitalis, 2, cortical area

aparc-pial_rh_area_parstriangularis, 2, cortical area

aparc-pial_rh_area_pericalcarine, 2, cortical area

aparc-pial_rh_area_postcentral, 2, cortical area

aparc-pial_rh_area_posteriorcingulate, 2, cortical area

aparc-pial_rh_area_precentral, 2, cortical area

aparc-pial_rh_area_precuneus, 2, cortical area

aparc-pial_rh_area_rostralanteriorcingulate, 2, cortical area

aparc-pial_rh_area_rostralmiddlefrontal, 2, cortical area

aparc-pial_rh_area_superiorfrontal, 2, cortical area

aparc-pial_rh_area_superiorparietal, 2, cortical area

aparc-pial_rh_area_superiortemporal, 2, cortical area

aparc-pial_rh_area_supramarginal, 2, cortical area

aparc-pial_rh_area_frontalpole, 2, cortical area

aparc-pial_rh_area_transversetemporal, 2, cortical area

BA-exvivo_lh_area_BA1, 2, cortical area

BA-exvivo_lh_area_BA2, 2, cortical area

BA-exvivo_lh_area_BA3a, 2, cortical area

BA-exvivo_lh_area_BA3b, 2, cortical area

BA-exvivo_lh_area_BA4a, 2, cortical area

BA-exvivo_lh_area_BA4p, 2, cortical area

BA-exvivo_lh_area_BA6, 2, cortical area

BA-exvivo_lh_area_BA44, 2, cortical area

BA-exvivo_lh_area_BA45, 2, cortical area

BA-exvivo_lh_area_V1, 2, cortical area

BA-exvivo_lh_area_V2, 2, cortical area

BA-exvivo_lh_area_MT, 2, cortical area

BA-exvivo_lh_area_perirhinal, 2, cortical area

BA-exvivo_lh_area_entorhinal, 2, cortical area

BA-exvivo_rh_area_BA1, 2, cortical area

BA-exvivo_rh_area_BA2, 2, cortical area

BA-exvivo_rh_area_BA3a, 2, cortical area

BA-exvivo_rh_area_BA3b, 2, cortical area

BA-exvivo_rh_area_BA4a, 2, cortical area

BA-exvivo_rh_area_BA4p, 2, cortical area

BA-exvivo_rh_area_BA6, 2, cortical area

BA-exvivo_rh_area_BA44, 2, cortical area

BA-exvivo_rh_area_BA45, 2, cortical area

BA-exvivo_rh_area_V1, 2, cortical area

BA-exvivo_rh_area_V2, 2, cortical area

BA-exvivo_rh_area_MT, 2, cortical area

BA-exvivo_rh_area_perirhinal, 2, cortical area

BA-exvivo_rh_area_entorhinal, 2, cortical area

aparc-DKTatlas_lh_area_caudalanteriorcingulate, 2, cortical area

aparc-DKTatlas_lh_area_caudalmiddlefrontal, 2, cortical area

aparc-DKTatlas_lh_area_cuneus, 2, cortical area

aparc-DKTatlas_lh_area_entorhinal, 2, cortical area

aparc-DKTatlas_lh_area_fusiform, 2, cortical area

aparc-DKTatlas_lh_area_inferiorparietal, 2, cortical area

aparc-DKTatlas_lh_area_inferiortemporal, 2, cortical area

aparc-DKTatlas_lh_area_isthmuscingulate, 2, cortical area

aparc-DKTatlas_lh_area_lateraloccipital, 2, cortical area

aparc-DKTatlas_lh_area_lateralorbitofrontal, 2, cortical area

aparc-DKTatlas_lh_area_lingual, 2, cortical area

aparc-DKTatlas_lh_area_medialorbitofrontal, 2, cortical area

aparc-DKTatlas_lh_area_middletemporal, 2, cortical area

aparc-DKTatlas_lh_area_parahippocampal, 2, cortical area

aparc-DKTatlas_lh_area_paracentral, 2, cortical area

aparc-DKTatlas_lh_area_parsopercularis, 2, cortical area

aparc-DKTatlas_lh_area_parsorbitalis, 2, cortical area

aparc-DKTatlas_lh_area_parstriangularis, 2, cortical area

aparc-DKTatlas_lh_area_pericalcarine, 2, cortical area

aparc-DKTatlas_lh_area_postcentral, 2, cortical area

aparc-DKTatlas_lh_area_posteriorcingulate, 2, cortical area

aparc-DKTatlas_lh_area_precentral, 2, cortical area

aparc-DKTatlas_lh_area_precuneus, 2, cortical area

aparc-DKTatlas_lh_area_rostralanteriorcingulate, 2, cortical area

aparc-DKTatlas_lh_area_rostralmiddlefrontal, 2, cortical area

aparc-DKTatlas_lh_area_superiorfrontal, 2, cortical area

aparc-DKTatlas_lh_area_superiorparietal, 2, cortical area

aparc-DKTatlas_lh_area_superiortemporal, 2, cortical area

aparc-DKTatlas_lh_area_supramarginal, 2, cortical area

aparc-DKTatlas_lh_area_transversetemporal, 2, cortical area

aparc-DKTatlas_lh_area_insula, 2, cortical area

aparc-DKTatlas_rh_area_caudalanteriorcingulate, 2, cortical area

aparc-DKTatlas_rh_area_caudalmiddlefrontal, 2, cortical area

aparc-DKTatlas_rh_area_cuneus, 2, cortical area

aparc-DKTatlas_rh_area_entorhinal, 2, cortical area

aparc-DKTatlas_rh_area_fusiform, 2, cortical area

aparc-DKTatlas_rh_area_inferiorparietal, 2, cortical area

aparc-DKTatlas_rh_area_inferiortemporal, 2, cortical area

aparc-DKTatlas_rh_area_isthmuscingulate, 2, cortical area

aparc-DKTatlas_rh_area_lateraloccipital, 2, cortical area

aparc-DKTatlas_rh_area_lateralorbitofrontal, 2, cortical area

aparc-DKTatlas_rh_area_lingual, 2, cortical area

aparc-DKTatlas_rh_area_medialorbitofrontal, 2, cortical area

aparc-DKTatlas_rh_area_middletemporal, 2, cortical area

aparc-DKTatlas_rh_area_parahippocampal, 2, cortical area

aparc-DKTatlas_rh_area_paracentral, 2, cortical area

aparc-DKTatlas_rh_area_parsopercularis, 2, cortical area

aparc-DKTatlas_rh_area_parsorbitalis, 2, cortical area

aparc-DKTatlas_rh_area_parstriangularis, 2, cortical area

aparc-DKTatlas_rh_area_pericalcarine, 2, cortical area

aparc-DKTatlas_rh_area_postcentral, 2, cortical area

aparc-DKTatlas_rh_area_posteriorcingulate, 2, cortical area

aparc-DKTatlas_rh_area_precentral, 2, cortical area

aparc-DKTatlas_rh_area_precuneus, 2, cortical area

aparc-DKTatlas_rh_area_rostralanteriorcingulate, 2, cortical area

aparc-DKTatlas_rh_area_rostralmiddlefrontal, 2, cortical area

aparc-DKTatlas_rh_area_superiorfrontal, 2, cortical area

aparc-DKTatlas_rh_area_superiorparietal, 2, cortical area

aparc-DKTatlas_rh_area_superiortemporal, 2, cortical area

aparc-DKTatlas_rh_area_supramarginal, 2, cortical area

aparc-DKTatlas_rh_area_transversetemporal, 2, cortical area

aparc-DKTatlas_rh_area_insula, 2, cortical area

aparc-a2009s_lh_area_G+S-frontomargin, 2, cortical area

aparc-a2009s_lh_area_G+S-occipital-inf, 2, cortical area

aparc-a2009s_lh_area_G+S-paracentral, 2, cortical area

aparc-a2009s_lh_area_G+S-subcentral, 2, cortical area

aparc-a2009s_lh_area_G+S-transv-frontopol, 2, cortical area

aparc-a2009s_lh_area_G+S-cingul-Ant, 2, cortical area

aparc-a2009s_lh_area_G+S-cingul-Mid-Ant, 2, cortical area

aparc-a2009s_lh_area_G+S-cingul-Mid-Post, 2, cortical area

aparc-a2009s_lh_area_G-cingul-Post-dorsal, 2, cortical area

aparc-a2009s_lh_area_G-cingul-Post-ventral, 2, cortical area

aparc-a2009s_lh_area_G-cuneus, 2, cortical area

aparc-a2009s_lh_area_G-front-inf-Opercular, 2, cortical area

aparc-a2009s_lh_area_G-front-inf-Orbital, 2, cortical area

aparc-a2009s_lh_area_G-front-inf-Triangul, 2, cortical area

aparc-a2009s_lh_area_G-front-middle, 2, cortical area

aparc-a2009s_lh_area_G-front-sup, 2, cortical area

aparc-a2009s_lh_area_G-Ins-lg+S-cent-ins, 2, cortical area

aparc-a2009s_lh_area_G-insular-short, 2, cortical area

aparc-a2009s_lh_area_G-occipital-middle, 2, cortical area

aparc-a2009s_lh_area_G-occipital-sup, 2, cortical area

aparc-a2009s_lh_area_G-oc-temp-lat-fusifor, 2, cortical area

aparc-a2009s_lh_area_G-oc-temp-med-Lingual, 2, cortical area

aparc-a2009s_lh_area_G-oc-temp-med-Parahip, 2, cortical area

aparc-a2009s_lh_area_G-orbital, 2, cortical area

aparc-a2009s_lh_area_G-pariet-inf-Angular, 2, cortical area

aparc-a2009s_lh_area_G-pariet-inf-Supramar, 2, cortical area

aparc-a2009s_lh_area_G-parietal-sup, 2, cortical area

aparc-a2009s_lh_area_G-postcentral, 2, cortical area

aparc-a2009s_lh_area_G-precentral, 2, cortical area

aparc-a2009s_lh_area_G-precuneus, 2, cortical area

aparc-a2009s_lh_area_G-rectus, 2, cortical area

aparc-a2009s_lh_area_G-subcallosal, 2, cortical area

aparc-a2009s_lh_area_G-temp-sup-G-T-transv, 2, cortical area

aparc-a2009s_lh_area_G-temp-sup-Lateral, 2, cortical area

aparc-a2009s_lh_area_G-temp-sup-Plan-polar, 2, cortical area

aparc-a2009s_lh_area_G-temp-sup-Plan-tempo, 2, cortical area

aparc-a2009s_lh_area_G-temporal-inf, 2, cortical area

aparc-a2009s_lh_area_G-temporal-middle, 2, cortical area

aparc-a2009s_lh_area_Lat-Fis-ant-Horizont, 2, cortical area

aparc-a2009s_lh_area_Lat-Fis-ant-Vertical, 2, cortical area

aparc-a2009s_lh_area_Lat-Fis-post, 2, cortical area

aparc-a2009s_lh_area_Pole-occipital, 2, cortical area

aparc-a2009s_lh_area_Pole-temporal, 2, cortical area

aparc-a2009s_lh_area_S-calcarine, 2, cortical area

aparc-a2009s_lh_area_S-central, 2, cortical area

aparc-a2009s_lh_area_S-cingul-Marginalis, 2, cortical area

aparc-a2009s_lh_area_S-circular-insula-ant, 2, cortical area

aparc-a2009s_lh_area_S-circular-insula-inf, 2, cortical area

aparc-a2009s_lh_area_S-circular-insula-sup, 2, cortical area

aparc-a2009s_lh_area_S-collat-transv-ant, 2, cortical area

aparc-a2009s_lh_area_S-collat-transv-post, 2, cortical area

aparc-a2009s_lh_area_S-front-inf, 2, cortical area

aparc-a2009s_lh_area_S-front-middle, 2, cortical area

aparc-a2009s_lh_area_S-front-sup, 2, cortical area

aparc-a2009s_lh_area_S-interm-prim-Jensen, 2, cortical area

aparc-a2009s_lh_area_S-intrapariet+P-trans, 2, cortical area

aparc-a2009s_lh_area_S-oc-middle+Lunatus, 2, cortical area

aparc-a2009s_lh_area_S-oc-sup+transversal, 2, cortical area

aparc-a2009s_lh_area_S-occipital-ant, 2, cortical area

aparc-a2009s_lh_area_S-oc-temp-lat, 2, cortical area

aparc-a2009s_lh_area_S-oc-temp-med+Lingual, 2, cortical area

aparc-a2009s_lh_area_S-orbital-lateral, 2, cortical area

aparc-a2009s_lh_area_S-orbital-med-olfact, 2, cortical area

aparc-a2009s_lh_area_S-orbital-H-Shaped, 2, cortical area

aparc-a2009s_lh_area_S-parieto-occipital, 2, cortical area

aparc-a2009s_lh_area_S-pericallosal, 2, cortical area

aparc-a2009s_lh_area_S-postcentral, 2, cortical area

aparc-a2009s_lh_area_S-precentral-inf-part, 2, cortical area

aparc-a2009s_lh_area_S-precentral-sup-part, 2, cortical area

aparc-a2009s_lh_area_S-suborbital, 2, cortical area

aparc-a2009s_lh_area_S-subparietal, 2, cortical area

aparc-a2009s_lh_area_S-temporal-inf, 2, cortical area

aparc-a2009s_lh_area_S-temporal-sup, 2, cortical area

aparc-a2009s_lh_area_S-temporal-transverse, 2, cortical area

aparc-a2009s_rh_area_G+S-frontomargin, 2, cortical area

aparc-a2009s_rh_area_G+S-occipital-inf, 2, cortical area

aparc-a2009s_rh_area_G+S-paracentral, 2, cortical area

aparc-a2009s_rh_area_G+S-subcentral, 2, cortical area

aparc-a2009s_rh_area_G+S-transv-frontopol, 2, cortical area

aparc-a2009s_rh_area_G+S-cingul-Ant, 2, cortical area

aparc-a2009s_rh_area_G+S-cingul-Mid-Ant, 2, cortical area

aparc-a2009s_rh_area_G+S-cingul-Mid-Post, 2, cortical area

aparc-a2009s_rh_area_G-cingul-Post-dorsal, 2, cortical area

aparc-a2009s_rh_area_G-cingul-Post-ventral, 2, cortical area

aparc-a2009s_rh_area_G-cuneus, 2, cortical area

aparc-a2009s_rh_area_G-front-inf-Opercular, 2, cortical area

aparc-a2009s_rh_area_G-front-inf-Orbital, 2, cortical area

aparc-a2009s_rh_area_G-front-inf-Triangul, 2, cortical area

aparc-a2009s_rh_area_G-front-middle, 2, cortical area

aparc-a2009s_rh_area_G-front-sup, 2, cortical area

aparc-a2009s_rh_area_G-Ins-lg+S-cent-ins, 2, cortical area

aparc-a2009s_rh_area_G-insular-short, 2, cortical area

aparc-a2009s_rh_area_G-occipital-middle, 2, cortical area

aparc-a2009s_rh_area_G-occipital-sup, 2, cortical area

aparc-a2009s_rh_area_G-oc-temp-lat-fusifor, 2, cortical area

aparc-a2009s_rh_area_G-oc-temp-med-Lingual, 2, cortical area

aparc-a2009s_rh_area_G-oc-temp-med-Parahip, 2, cortical area

aparc-a2009s_rh_area_G-orbital, 2, cortical area

aparc-a2009s_rh_area_G-pariet-inf-Angular, 2, cortical area

aparc-a2009s_rh_area_G-pariet-inf-Supramar, 2, cortical area

aparc-a2009s_rh_area_G-parietal-sup, 2, cortical area

aparc-a2009s_rh_area_G-postcentral, 2, cortical area

aparc-a2009s_rh_area_G-precentral, 2, cortical area

aparc-a2009s_rh_area_G-precuneus, 2, cortical area

aparc-a2009s_rh_area_G-rectus, 2, cortical area

aparc-a2009s_rh_area_G-subcallosal, 2, cortical area

aparc-a2009s_rh_area_G-temp-sup-G-T-transv, 2, cortical area

aparc-a2009s_rh_area_G-temp-sup-Lateral, 2, cortical area

aparc-a2009s_rh_area_G-temp-sup-Plan-polar, 2, cortical area

aparc-a2009s_rh_area_G-temp-sup-Plan-tempo, 2, cortical area

aparc-a2009s_rh_area_G-temporal-inf, 2, cortical area

aparc-a2009s_rh_area_G-temporal-middle, 2, cortical area

aparc-a2009s_rh_area_Lat-Fis-ant-Horizont, 2, cortical area

aparc-a2009s_rh_area_Lat-Fis-ant-Vertical, 2, cortical area

aparc-a2009s_rh_area_Lat-Fis-post, 2, cortical area

aparc-a2009s_rh_area_Pole-occipital, 2, cortical area

aparc-a2009s_rh_area_Pole-temporal, 2, cortical area

aparc-a2009s_rh_area_S-calcarine, 2, cortical area

aparc-a2009s_rh_area_S-central, 2, cortical area

aparc-a2009s_rh_area_S-cingul-Marginalis, 2, cortical area

aparc-a2009s_rh_area_S-circular-insula-ant, 2, cortical area

aparc-a2009s_rh_area_S-circular-insula-inf, 2, cortical area

aparc-a2009s_rh_area_S-circular-insula-sup, 2, cortical area

aparc-a2009s_rh_area_S-collat-transv-ant, 2, cortical area

aparc-a2009s_rh_area_S-collat-transv-post, 2, cortical area

aparc-a2009s_rh_area_S-front-inf, 2, cortical area

aparc-a2009s_rh_area_S-front-middle, 2, cortical area

aparc-a2009s_rh_area_S-front-sup, 2, cortical area

aparc-a2009s_rh_area_S-interm-prim-Jensen, 2, cortical area

aparc-a2009s_rh_area_S-intrapariet+P-trans, 2, cortical area

aparc-a2009s_rh_area_S-oc-middle+Lunatus, 2, cortical area

aparc-a2009s_rh_area_S-oc-sup+transversal, 2, cortical area

aparc-a2009s_rh_area_S-occipital-ant, 2, cortical area

aparc-a2009s_rh_area_S-oc-temp-lat, 2, cortical area

aparc-a2009s_rh_area_S-oc-temp-med+Lingual, 2, cortical area

aparc-a2009s_rh_area_S-orbital-lateral, 2, cortical area

aparc-a2009s_rh_area_S-orbital-med-olfact, 2, cortical area

aparc-a2009s_rh_area_S-orbital-H-Shaped, 2, cortical area

aparc-a2009s_rh_area_S-parieto-occipital, 2, cortical area

aparc-a2009s_rh_area_S-pericallosal, 2, cortical area

aparc-a2009s_rh_area_S-postcentral, 2, cortical area

aparc-a2009s_rh_area_S-precentral-inf-part, 2, cortical area

aparc-a2009s_rh_area_S-precentral-sup-part, 2, cortical area

aparc-a2009s_rh_area_S-suborbital, 2, cortical area

aparc-a2009s_rh_area_S-subparietal, 2, cortical area

aparc-a2009s_rh_area_S-temporal-inf, 2, cortical area

aparc-a2009s_rh_area_S-temporal-sup, 2, cortical area

aparc-a2009s_rh_area_S-temporal-transverse, 2, cortical area

aparc-Desikan_lh_thickness_GlobalMeanThickness, 3, cortical thickness

aparc-Desikan_lh_thickness_bankssts, 3, cortical thickness

aparc-Desikan_lh_thickness_caudalanteriorcingulate, 3, cortical thickness

aparc-Desikan_lh_thickness_caudalmiddlefrontal, 3, cortical thickness

aparc-Desikan_lh_thickness_cuneus, 3, cortical thickness

aparc-Desikan_lh_thickness_entorhinal, 3, cortical thickness

aparc-Desikan_lh_thickness_fusiform, 3, cortical thickness

aparc-Desikan_lh_thickness_inferiorparietal, 3, cortical thickness

aparc-Desikan_lh_thickness_inferiortemporal, 3, cortical thickness

aparc-Desikan_lh_thickness_isthmuscingulate, 3, cortical thickness

aparc-Desikan_lh_thickness_lateraloccipital, 3, cortical thickness

aparc-Desikan_lh_thickness_lateralorbitofrontal, 3, cortical thickness

aparc-Desikan_lh_thickness_lingual, 3, cortical thickness

aparc-Desikan_lh_thickness_medialorbitofrontal, 3, cortical thickness

aparc-Desikan_lh_thickness_middletemporal, 3, cortical thickness

aparc-Desikan_lh_thickness_parahippocampal, 3, cortical thickness

aparc-Desikan_lh_thickness_paracentral, 3, cortical thickness

aparc-Desikan_lh_thickness_parsopercularis, 3, cortical thickness

aparc-Desikan_lh_thickness_parsorbitalis, 3, cortical thickness

aparc-Desikan_lh_thickness_parstriangularis, 3, cortical thickness

aparc-Desikan_lh_thickness_pericalcarine, 3, cortical thickness

aparc-Desikan_lh_thickness_postcentral, 3, cortical thickness

aparc-Desikan_lh_thickness_posteriorcingulate, 3, cortical thickness

aparc-Desikan_lh_thickness_precentral, 3, cortical thickness

aparc-Desikan_lh_thickness_precuneus, 3, cortical thickness

aparc-Desikan_lh_thickness_rostralanteriorcingulate, 3, cortical thickness

aparc-Desikan_lh_thickness_rostralmiddlefrontal, 3, cortical thickness

aparc-Desikan_lh_thickness_superiorfrontal, 3, cortical thickness

aparc-Desikan_lh_thickness_superiorparietal, 3, cortical thickness

aparc-Desikan_lh_thickness_superiortemporal, 3, cortical thickness

aparc-Desikan_lh_thickness_supramarginal, 3, cortical thickness

aparc-Desikan_lh_thickness_frontalpole, 3, cortical thickness

aparc-Desikan_lh_thickness_transversetemporal, 3, cortical thickness

aparc-Desikan_lh_thickness_insula, 3, cortical thickness

aparc-Desikan_rh_thickness_GlobalMeanThickness, 3, cortical thickness

aparc-Desikan_rh_thickness_bankssts, 3, cortical thickness

aparc-Desikan_rh_thickness_caudalanteriorcingulate, 3, cortical thickness

aparc-Desikan_rh_thickness_caudalmiddlefrontal, 3, cortical thickness

aparc-Desikan_rh_thickness_cuneus, 3, cortical thickness

aparc-Desikan_rh_thickness_entorhinal, 3, cortical thickness

aparc-Desikan_rh_thickness_fusiform, 3, cortical thickness

aparc-Desikan_rh_thickness_inferiorparietal, 3, cortical thickness

aparc-Desikan_rh_thickness_inferiortemporal, 3, cortical thickness

aparc-Desikan_rh_thickness_isthmuscingulate, 3, cortical thickness

aparc-Desikan_rh_thickness_lateraloccipital, 3, cortical thickness

aparc-Desikan_rh_thickness_lateralorbitofrontal, 3, cortical thickness

aparc-Desikan_rh_thickness_lingual, 3, cortical thickness

aparc-Desikan_rh_thickness_medialorbitofrontal, 3, cortical thickness

aparc-Desikan_rh_thickness_middletemporal, 3, cortical thickness

aparc-Desikan_rh_thickness_parahippocampal, 3, cortical thickness

aparc-Desikan_rh_thickness_paracentral, 3, cortical thickness

aparc-Desikan_rh_thickness_parsopercularis, 3, cortical thickness

aparc-Desikan_rh_thickness_parsorbitalis, 3, cortical thickness

aparc-Desikan_rh_thickness_parstriangularis, 3, cortical thickness

aparc-Desikan_rh_thickness_pericalcarine, 3, cortical thickness

aparc-Desikan_rh_thickness_postcentral, 3, cortical thickness

aparc-Desikan_rh_thickness_posteriorcingulate, 3, cortical thickness

aparc-Desikan_rh_thickness_precentral, 3, cortical thickness

aparc-Desikan_rh_thickness_precuneus, 3, cortical thickness

aparc-Desikan_rh_thickness_rostralanteriorcingulate, 3, cortical thickness

aparc-Desikan_rh_thickness_rostralmiddlefrontal, 3, cortical thickness

aparc-Desikan_rh_thickness_superiorfrontal, 3, cortical thickness

aparc-Desikan_rh_thickness_superiorparietal, 3, cortical thickness

aparc-Desikan_rh_thickness_superiortemporal, 3, cortical thickness

aparc-Desikan_rh_thickness_supramarginal, 3, cortical thickness

aparc-Desikan_rh_thickness_frontalpole, 3, cortical thickness

aparc-Desikan_rh_thickness_transversetemporal, 3, cortical thickness

aparc-Desikan_rh_thickness_insula, 3, cortical thickness

BA-exvivo_lh_thickness_BA1, 3, cortical thickness

BA-exvivo_lh_thickness_BA2, 3, cortical thickness

BA-exvivo_lh_thickness_BA3a, 3, cortical thickness

BA-exvivo_lh_thickness_BA3b, 3, cortical thickness

BA-exvivo_lh_thickness_BA4a, 3, cortical thickness

BA-exvivo_lh_thickness_BA4p, 3, cortical thickness

BA-exvivo_lh_thickness_BA6, 3, cortical thickness

BA-exvivo_lh_thickness_BA44, 3, cortical thickness

BA-exvivo_lh_thickness_BA45, 3, cortical thickness

BA-exvivo_lh_thickness_V1, 3, cortical thickness

BA-exvivo_lh_thickness_V2, 3, cortical thickness

BA-exvivo_lh_thickness_MT, 3, cortical thickness

BA-exvivo_lh_thickness_perirhinal, 3, cortical thickness

BA-exvivo_lh_thickness_entorhinal, 3, cortical thickness

BA-exvivo_rh_thickness_BA1, 3, cortical thickness

BA-exvivo_rh_thickness_BA2, 3, cortical thickness

BA-exvivo_rh_thickness_BA3a, 3, cortical thickness

BA-exvivo_rh_thickness_BA3b, 3, cortical thickness

BA-exvivo_rh_thickness_BA4a, 3, cortical thickness

BA-exvivo_rh_thickness_BA4p, 3, cortical thickness

BA-exvivo_rh_thickness_BA6, 3, cortical thickness

BA-exvivo_rh_thickness_BA44, 3, cortical thickness

BA-exvivo_rh_thickness_BA45, 3, cortical thickness

BA-exvivo_rh_thickness_V1, 3, cortical thickness

BA-exvivo_rh_thickness_V2, 3, cortical thickness

BA-exvivo_rh_thickness_MT, 3, cortical thickness

BA-exvivo_rh_thickness_perirhinal, 3, cortical thickness

BA-exvivo_rh_thickness_entorhinal, 3, cortical thickness

aparc-DKTatlas_lh_thickness_caudalanteriorcingulate, 3, cortical thickness

aparc-DKTatlas_lh_thickness_caudalmiddlefrontal, 3, cortical thickness

aparc-DKTatlas_lh_thickness_cuneus, 3, cortical thickness

aparc-DKTatlas_lh_thickness_entorhinal, 3, cortical thickness

aparc-DKTatlas_lh_thickness_fusiform, 3, cortical thickness

aparc-DKTatlas_lh_thickness_inferiorparietal, 3, cortical thickness

aparc-DKTatlas_lh_thickness_inferiortemporal, 3, cortical thickness

aparc-DKTatlas_lh_thickness_isthmuscingulate, 3, cortical thickness

aparc-DKTatlas_lh_thickness_lateraloccipital, 3, cortical thickness

aparc-DKTatlas_lh_thickness_lateralorbitofrontal, 3, cortical thickness

aparc-DKTatlas_lh_thickness_lingual, 3, cortical thickness

aparc-DKTatlas_lh_thickness_medialorbitofrontal, 3, cortical thickness

aparc-DKTatlas_lh_thickness_middletemporal, 3, cortical thickness

aparc-DKTatlas_lh_thickness_parahippocampal, 3, cortical thickness

aparc-DKTatlas_lh_thickness_paracentral, 3, cortical thickness

aparc-DKTatlas_lh_thickness_parsopercularis, 3, cortical thickness

aparc-DKTatlas_lh_thickness_parsorbitalis, 3, cortical thickness

aparc-DKTatlas_lh_thickness_parstriangularis, 3, cortical thickness

aparc-DKTatlas_lh_thickness_pericalcarine, 3, cortical thickness

aparc-DKTatlas_lh_thickness_postcentral, 3, cortical thickness

aparc-DKTatlas_lh_thickness_posteriorcingulate, 3, cortical thickness

aparc-DKTatlas_lh_thickness_precentral, 3, cortical thickness

aparc-DKTatlas_lh_thickness_precuneus, 3, cortical thickness

aparc-DKTatlas_lh_thickness_rostralanteriorcingulate, 3, cortical thickness

aparc-DKTatlas_lh_thickness_rostralmiddlefrontal, 3, cortical thickness

aparc-DKTatlas_lh_thickness_superiorfrontal, 3, cortical thickness

aparc-DKTatlas_lh_thickness_superiorparietal, 3, cortical thickness

aparc-DKTatlas_lh_thickness_superiortemporal, 3, cortical thickness

aparc-DKTatlas_lh_thickness_supramarginal, 3, cortical thickness

aparc-DKTatlas_lh_thickness_transversetemporal, 3, cortical thickness

aparc-DKTatlas_lh_thickness_insula, 3, cortical thickness

aparc-DKTatlas_rh_thickness_caudalanteriorcingulate, 3, cortical thickness

aparc-DKTatlas_rh_thickness_caudalmiddlefrontal, 3, cortical thickness

aparc-DKTatlas_rh_thickness_cuneus, 3, cortical thickness

aparc-DKTatlas_rh_thickness_entorhinal, 3, cortical thickness

aparc-DKTatlas_rh_thickness_fusiform, 3, cortical thickness

aparc-DKTatlas_rh_thickness_inferiorparietal, 3, cortical thickness

aparc-DKTatlas_rh_thickness_inferiortemporal, 3, cortical thickness

aparc-DKTatlas_rh_thickness_isthmuscingulate, 3, cortical thickness

aparc-DKTatlas_rh_thickness_lateraloccipital, 3, cortical thickness

aparc-DKTatlas_rh_thickness_lateralorbitofrontal, 3, cortical thickness

aparc-DKTatlas_rh_thickness_lingual, 3, cortical thickness

aparc-DKTatlas_rh_thickness_medialorbitofrontal, 3, cortical thickness

aparc-DKTatlas_rh_thickness_middletemporal, 3, cortical thickness

aparc-DKTatlas_rh_thickness_parahippocampal, 3, cortical thickness

aparc-DKTatlas_rh_thickness_paracentral, 3, cortical thickness

aparc-DKTatlas_rh_thickness_parsopercularis, 3, cortical thickness

aparc-DKTatlas_rh_thickness_parsorbitalis, 3, cortical thickness

aparc-DKTatlas_rh_thickness_parstriangularis, 3, cortical thickness

aparc-DKTatlas_rh_thickness_pericalcarine, 3, cortical thickness

aparc-DKTatlas_rh_thickness_postcentral, 3, cortical thickness

aparc-DKTatlas_rh_thickness_posteriorcingulate, 3, cortical thickness

aparc-DKTatlas_rh_thickness_precentral, 3, cortical thickness

aparc-DKTatlas_rh_thickness_precuneus, 3, cortical thickness

aparc-DKTatlas_rh_thickness_rostralanteriorcingulate, 3, cortical thickness

aparc-DKTatlas_rh_thickness_rostralmiddlefrontal, 3, cortical thickness

aparc-DKTatlas_rh_thickness_superiorfrontal, 3, cortical thickness

aparc-DKTatlas_rh_thickness_superiorparietal, 3, cortical thickness

aparc-DKTatlas_rh_thickness_superiortemporal, 3, cortical thickness

aparc-DKTatlas_rh_thickness_supramarginal, 3, cortical thickness

aparc-DKTatlas_rh_thickness_transversetemporal, 3, cortical thickness

aparc-DKTatlas_rh_thickness_insula, 3, cortical thickness

aparc-a2009s_lh_thickness_G+S-frontomargin, 3, cortical thickness

aparc-a2009s_lh_thickness_G+S-occipital-inf, 3, cortical thickness

aparc-a2009s_lh_thickness_G+S-paracentral, 3, cortical thickness

aparc-a2009s_lh_thickness_G+S-subcentral, 3, cortical thickness

aparc-a2009s_lh_thickness_G+S-transv-frontopol, 3, cortical thickness

aparc-a2009s_lh_thickness_G+S-cingul-Ant, 3, cortical thickness

aparc-a2009s_lh_thickness_G+S-cingul-Mid-Ant, 3, cortical thickness

aparc-a2009s_lh_thickness_G+S-cingul-Mid-Post, 3, cortical thickness

aparc-a2009s_lh_thickness_G-cingul-Post-dorsal, 3, cortical thickness

aparc-a2009s_lh_thickness_G-cingul-Post-ventral, 3, cortical thickness

aparc-a2009s_lh_thickness_G-cuneus, 3, cortical thickness

aparc-a2009s_lh_thickness_G-front-inf-Opercular, 3, cortical thickness

aparc-a2009s_lh_thickness_G-front-inf-Orbital, 3, cortical thickness

aparc-a2009s_lh_thickness_G-front-inf-Triangul, 3, cortical thickness

aparc-a2009s_lh_thickness_G-front-middle, 3, cortical thickness

aparc-a2009s_lh_thickness_G-front-sup, 3, cortical thickness

aparc-a2009s_lh_thickness_G-Ins-lg+S-cent-ins, 3, cortical thickness

aparc-a2009s_lh_thickness_G-insular-short, 3, cortical thickness

aparc-a2009s_lh_thickness_G-occipital-middle, 3, cortical thickness

aparc-a2009s_lh_thickness_G-occipital-sup, 3, cortical thickness

aparc-a2009s_lh_thickness_G-oc-temp-lat-fusifor, 3, cortical thickness

aparc-a2009s_lh_thickness_G-oc-temp-med-Lingual, 3, cortical thickness

aparc-a2009s_lh_thickness_G-oc-temp-med-Parahip, 3, cortical thickness

aparc-a2009s_lh_thickness_G-orbital, 3, cortical thickness

aparc-a2009s_lh_thickness_G-pariet-inf-Angular, 3, cortical thickness

aparc-a2009s_lh_thickness_G-pariet-inf-Supramar, 3, cortical thickness

aparc-a2009s_lh_thickness_G-parietal-sup, 3, cortical thickness

aparc-a2009s_lh_thickness_G-postcentral, 3, cortical thickness

aparc-a2009s_lh_thickness_G-precentral, 3, cortical thickness

aparc-a2009s_lh_thickness_G-precuneus, 3, cortical thickness

aparc-a2009s_lh_thickness_G-rectus, 3, cortical thickness

aparc-a2009s_lh_thickness_G-subcallosal, 3, cortical thickness

aparc-a2009s_lh_thickness_G-temp-sup-G-T-transv, 3, cortical thickness

aparc-a2009s_lh_thickness_G-temp-sup-Lateral, 3, cortical thickness

aparc-a2009s_lh_thickness_G-temp-sup-Plan-polar, 3, cortical thickness

aparc-a2009s_lh_thickness_G-temp-sup-Plan-tempo, 3, cortical thickness

aparc-a2009s_lh_thickness_G-temporal-inf, 3, cortical thickness

aparc-a2009s_lh_thickness_G-temporal-middle, 3, cortical thickness

aparc-a2009s_lh_thickness_Lat-Fis-ant-Horizont, 3, cortical thickness

aparc-a2009s_lh_thickness_Lat-Fis-ant-Vertical, 3, cortical thickness

aparc-a2009s_lh_thickness_Lat-Fis-post, 3, cortical thickness

aparc-a2009s_lh_thickness_Pole-occipital, 3, cortical thickness

aparc-a2009s_lh_thickness_Pole-temporal, 3, cortical thickness

aparc-a2009s_lh_thickness_S-calcarine, 3, cortical thickness

aparc-a2009s_lh_thickness_S-central, 3, cortical thickness

aparc-a2009s_lh_thickness_S-cingul-Marginalis, 3, cortical thickness

aparc-a2009s_lh_thickness_S-circular-insula-ant, 3, cortical thickness

aparc-a2009s_lh_thickness_S-circular-insula-inf, 3, cortical thickness

aparc-a2009s_lh_thickness_S-circular-insula-sup, 3, cortical thickness

aparc-a2009s_lh_thickness_S-collat-transv-ant, 3, cortical thickness

aparc-a2009s_lh_thickness_S-collat-transv-post, 3, cortical thickness

aparc-a2009s_lh_thickness_S-front-inf, 3, cortical thickness

aparc-a2009s_lh_thickness_S-front-middle, 3, cortical thickness

aparc-a2009s_lh_thickness_S-front-sup, 3, cortical thickness

aparc-a2009s_lh_thickness_S-interm-prim-Jensen, 3, cortical thickness

aparc-a2009s_lh_thickness_S-intrapariet+P-trans, 3, cortical thickness

aparc-a2009s_lh_thickness_S-oc-middle+Lunatus, 3, cortical thickness

aparc-a2009s_lh_thickness_S-oc-sup+transversal, 3, cortical thickness

aparc-a2009s_lh_thickness_S-occipital-ant, 3, cortical thickness

aparc-a2009s_lh_thickness_S-oc-temp-lat, 3, cortical thickness

aparc-a2009s_lh_thickness_S-oc-temp-med+Lingual, 3, cortical thickness

aparc-a2009s_lh_thickness_S-orbital-lateral, 3, cortical thickness

aparc-a2009s_lh_thickness_S-orbital-med-olfact, 3, cortical thickness

aparc-a2009s_lh_thickness_S-orbital-H-Shaped, 3, cortical thickness

aparc-a2009s_lh_thickness_S-parieto-occipital, 3, cortical thickness

aparc-a2009s_lh_thickness_S-pericallosal, 3, cortical thickness

aparc-a2009s_lh_thickness_S-postcentral, 3, cortical thickness

aparc-a2009s_lh_thickness_S-precentral-inf-part, 3, cortical thickness

aparc-a2009s_lh_thickness_S-precentral-sup-part, 3, cortical thickness

aparc-a2009s_lh_thickness_S-suborbital, 3, cortical thickness

aparc-a2009s_lh_thickness_S-subparietal, 3, cortical thickness

aparc-a2009s_lh_thickness_S-temporal-inf, 3, cortical thickness

aparc-a2009s_lh_thickness_S-temporal-sup, 3, cortical thickness

aparc-a2009s_lh_thickness_S-temporal-transverse, 3, cortical thickness

aparc-a2009s_rh_thickness_G+S-frontomargin, 3, cortical thickness

aparc-a2009s_rh_thickness_G+S-occipital-inf, 3, cortical thickness

aparc-a2009s_rh_thickness_G+S-paracentral, 3, cortical thickness

aparc-a2009s_rh_thickness_G+S-subcentral, 3, cortical thickness

aparc-a2009s_rh_thickness_G+S-transv-frontopol, 3, cortical thickness

aparc-a2009s_rh_thickness_G+S-cingul-Ant, 3, cortical thickness

aparc-a2009s_rh_thickness_G+S-cingul-Mid-Ant, 3, cortical thickness

aparc-a2009s_rh_thickness_G+S-cingul-Mid-Post, 3, cortical thickness

aparc-a2009s_rh_thickness_G-cingul-Post-dorsal, 3, cortical thickness

aparc-a2009s_rh_thickness_G-cingul-Post-ventral, 3, cortical thickness

aparc-a2009s_rh_thickness_G-cuneus, 3, cortical thickness

aparc-a2009s_rh_thickness_G-front-inf-Opercular, 3, cortical thickness

aparc-a2009s_rh_thickness_G-front-inf-Orbital, 3, cortical thickness

aparc-a2009s_rh_thickness_G-front-inf-Triangul, 3, cortical thickness

aparc-a2009s_rh_thickness_G-front-middle, 3, cortical thickness

aparc-a2009s_rh_thickness_G-front-sup, 3, cortical thickness

aparc-a2009s_rh_thickness_G-Ins-lg+S-cent-ins, 3, cortical thickness

aparc-a2009s_rh_thickness_G-insular-short, 3, cortical thickness

aparc-a2009s_rh_thickness_G-occipital-middle, 3, cortical thickness

aparc-a2009s_rh_thickness_G-occipital-sup, 3, cortical thickness

aparc-a2009s_rh_thickness_G-oc-temp-lat-fusifor, 3, cortical thickness

aparc-a2009s_rh_thickness_G-oc-temp-med-Lingual, 3, cortical thickness

aparc-a2009s_rh_thickness_G-oc-temp-med-Parahip, 3, cortical thickness

aparc-a2009s_rh_thickness_G-orbital, 3, cortical thickness

aparc-a2009s_rh_thickness_G-pariet-inf-Angular, 3, cortical thickness

aparc-a2009s_rh_thickness_G-pariet-inf-Supramar, 3, cortical thickness

aparc-a2009s_rh_thickness_G-parietal-sup, 3, cortical thickness

aparc-a2009s_rh_thickness_G-postcentral, 3, cortical thickness

aparc-a2009s_rh_thickness_G-precentral, 3, cortical thickness

aparc-a2009s_rh_thickness_G-precuneus, 3, cortical thickness

aparc-a2009s_rh_thickness_G-rectus, 3, cortical thickness

aparc-a2009s_rh_thickness_G-subcallosal, 3, cortical thickness

aparc-a2009s_rh_thickness_G-temp-sup-G-T-transv, 3, cortical thickness

aparc-a2009s_rh_thickness_G-temp-sup-Lateral, 3, cortical thickness

aparc-a2009s_rh_thickness_G-temp-sup-Plan-polar, 3, cortical thickness

aparc-a2009s_rh_thickness_G-temp-sup-Plan-tempo, 3, cortical thickness

aparc-a2009s_rh_thickness_G-temporal-inf, 3, cortical thickness

aparc-a2009s_rh_thickness_G-temporal-middle, 3, cortical thickness

aparc-a2009s_rh_thickness_Lat-Fis-ant-Horizont, 3, cortical thickness

aparc-a2009s_rh_thickness_Lat-Fis-ant-Vertical, 3, cortical thickness

aparc-a2009s_rh_thickness_Lat-Fis-post, 3, cortical thickness

aparc-a2009s_rh_thickness_Pole-occipital, 3, cortical thickness

aparc-a2009s_rh_thickness_Pole-temporal, 3, cortical thickness

aparc-a2009s_rh_thickness_S-calcarine, 3, cortical thickness

aparc-a2009s_rh_thickness_S-central, 3, cortical thickness

aparc-a2009s_rh_thickness_S-cingul-Marginalis, 3, cortical thickness

aparc-a2009s_rh_thickness_S-circular-insula-ant, 3, cortical thickness

aparc-a2009s_rh_thickness_S-circular-insula-inf, 3, cortical thickness

aparc-a2009s_rh_thickness_S-circular-insula-sup, 3, cortical thickness

aparc-a2009s_rh_thickness_S-collat-transv-ant, 3, cortical thickness

aparc-a2009s_rh_thickness_S-collat-transv-post, 3, cortical thickness

aparc-a2009s_rh_thickness_S-front-inf, 3, cortical thickness

aparc-a2009s_rh_thickness_S-front-middle, 3, cortical thickness

aparc-a2009s_rh_thickness_S-front-sup, 3, cortical thickness

aparc-a2009s_rh_thickness_S-interm-prim-Jensen, 3, cortical thickness

aparc-a2009s_rh_thickness_S-intrapariet+P-trans, 3, cortical thickness

aparc-a2009s_rh_thickness_S-oc-middle+Lunatus, 3, cortical thickness

aparc-a2009s_rh_thickness_S-oc-sup+transversal, 3, cortical thickness

aparc-a2009s_rh_thickness_S-occipital-ant, 3, cortical thickness

aparc-a2009s_rh_thickness_S-oc-temp-lat, 3, cortical thickness

aparc-a2009s_rh_thickness_S-oc-temp-med+Lingual, 3, cortical thickness

aparc-a2009s_rh_thickness_S-orbital-lateral, 3, cortical thickness

aparc-a2009s_rh_thickness_S-orbital-med-olfact, 3, cortical thickness

aparc-a2009s_rh_thickness_S-orbital-H-Shaped, 3, cortical thickness

aparc-a2009s_rh_thickness_S-parieto-occipital, 3, cortical thickness

aparc-a2009s_rh_thickness_S-pericallosal, 3, cortical thickness

aparc-a2009s_rh_thickness_S-postcentral, 3, cortical thickness

aparc-a2009s_rh_thickness_S-precentral-inf-part, 3, cortical thickness

aparc-a2009s_rh_thickness_S-precentral-sup-part, 3, cortical thickness

aparc-a2009s_rh_thickness_S-suborbital, 3, cortical thickness

aparc-a2009s_rh_thickness_S-subparietal, 3, cortical thickness

aparc-a2009s_rh_thickness_S-temporal-inf, 3, cortical thickness

aparc-a2009s_rh_thickness_S-temporal-sup, 3, cortical thickness

aparc-a2009s_rh_thickness_S-temporal-transverse, 3, cortical thickness

aseg_global_intensity_3rd-Ventricle, 4, regional and tissue intensity

aseg_global_intensity_4th-Ventricle, 4, regional and tissue intensity

aseg_global_intensity_5th-Ventricle, 4, regional and tissue intensity

aseg_global_intensity_Brain-Stem, 4, regional and tissue intensity

aseg_global_intensity_CSF, 4, regional and tissue intensity

aseg_global_intensity_WM-hypointensities, 4, regional and tissue intensity

aseg_global_intensity_non-WM-hypointensities, 4, regional and tissue intensity

aseg_global_intensity_Optic-Chiasm, 4, regional and tissue intensity

aseg_global_intensity_CC-Posterior, 4, regional and tissue intensity

aseg_global_intensity_CC-Mid-Posterior, 4, regional and tissue intensity

aseg_global_intensity_CC-Central, 4, regional and tissue intensity

aseg_global_intensity_CC-Mid-Anterior, 4, regional and tissue intensity

aseg_global_intensity_CC-Anterior, 4, regional and tissue intensity

aseg_lh_intensity_Lateral-Ventricle, 4, regional and tissue intensity

aseg_lh_intensity_Inf-Lat-Vent, 4, regional and tissue intensity

aseg_lh_intensity_Cerebellum-White-Matter, 4, regional and tissue intensity

aseg_lh_intensity_Cerebellum-Cortex, 4, regional and tissue intensity

aseg_lh_intensity_Thalamus-Proper, 4, regional and tissue intensity

aseg_lh_intensity_Caudate, 4, regional and tissue intensity

aseg_lh_intensity_Putamen, 4, regional and tissue intensity

aseg_lh_intensity_Pallidum, 4, regional and tissue intensity

aseg_lh_intensity_Hippocampus, 4, regional and tissue intensity

aseg_lh_intensity_Amygdala, 4, regional and tissue intensity

aseg_lh_intensity_Accumbens-area, 4, regional and tissue intensity

aseg_lh_intensity_VentralDC, 4, regional and tissue intensity

aseg_lh_intensity_vessel, 4, regional and tissue intensity

aseg_lh_intensity_choroid-plexus, 4, regional and tissue intensity

aseg_rh_intensity_Lateral-Ventricle, 4, regional and tissue intensity

aseg_rh_intensity_Inf-Lat-Vent, 4, regional and tissue intensity

aseg_rh_intensity_Cerebellum-White-Matter, 4, regional and tissue intensity

aseg_rh_intensity_Cerebellum-Cortex, 4, regional and tissue intensity

aseg_rh_intensity_Thalamus-Proper, 4, regional and tissue intensity

aseg_rh_intensity_Caudate, 4, regional and tissue intensity

aseg_rh_intensity_Putamen, 4, regional and tissue intensity

aseg_rh_intensity_Pallidum, 4, regional and tissue intensity

aseg_rh_intensity_Hippocampus, 4, regional and tissue intensity

aseg_rh_intensity_Amygdala, 4, regional and tissue intensity

aseg_rh_intensity_Accumbens-area, 4, regional and tissue intensity

aseg_rh_intensity_VentralDC, 4, regional and tissue intensity

aseg_rh_intensity_vessel, 4, regional and tissue intensity

aseg_rh_intensity_choroid-plexus, 4, regional and tissue intensity

wg_lh_intensity-contrast_unknown, 5, cortical grey-white contrast

wg_lh_intensity-contrast_bankssts, 5, cortical grey-white contrast

wg_lh_intensity-contrast_caudalanteriorcingulate, 5, cortical grey-white contrast

wg_lh_intensity-contrast_caudalmiddlefrontal, 5, cortical grey-white contrast

wg_lh_intensity-contrast_cuneus, 5, cortical grey-white contrast

wg_lh_intensity-contrast_entorhinal, 5, cortical grey-white contrast

wg_lh_intensity-contrast_fusiform, 5, cortical grey-white contrast

wg_lh_intensity-contrast_inferiorparietal, 5, cortical grey-white contrast

wg_lh_intensity-contrast_inferiortemporal, 5, cortical grey-white contrast

wg_lh_intensity-contrast_isthmuscingulate, 5, cortical grey-white contrast

wg_lh_intensity-contrast_lateraloccipital, 5, cortical grey-white contrast

wg_lh_intensity-contrast_lateralorbitofrontal, 5, cortical grey-white contrast

wg_lh_intensity-contrast_lingual, 5, cortical grey-white contrast

wg_lh_intensity-contrast_medialorbitofrontal, 5, cortical grey-white contrast

wg_lh_intensity-contrast_middletemporal, 5, cortical grey-white contrast

wg_lh_intensity-contrast_parahippocampal, 5, cortical grey-white contrast

wg_lh_intensity-contrast_paracentral, 5, cortical grey-white contrast

wg_lh_intensity-contrast_parsopercularis, 5, cortical grey-white contrast

wg_lh_intensity-contrast_parsorbitalis, 5, cortical grey-white contrast

wg_lh_intensity-contrast_parstriangularis, 5, cortical grey-white contrast

wg_lh_intensity-contrast_pericalcarine, 5, cortical grey-white contrast

wg_lh_intensity-contrast_postcentral, 5, cortical grey-white contrast

wg_lh_intensity-contrast_posteriorcingulate, 5, cortical grey-white contrast

wg_lh_intensity-contrast_precentral, 5, cortical grey-white contrast

wg_lh_intensity-contrast_precuneus, 5, cortical grey-white contrast

wg_lh_intensity-contrast_rostralanteriorcingulate, 5, cortical grey-white contrast

wg_lh_intensity-contrast_rostralmiddlefrontal, 5, cortical grey-white contrast

wg_lh_intensity-contrast_superiorfrontal, 5, cortical grey-white contrast

wg_lh_intensity-contrast_superiorparietal, 5, cortical grey-white contrast

wg_lh_intensity-contrast_superiortemporal, 5, cortical grey-white contrast

wg_lh_intensity-contrast_supramarginal, 5, cortical grey-white contrast

wg_lh_intensity-contrast_frontalpole, 5, cortical grey-white contrast

wg_lh_intensity-contrast_temporalpole, 5, cortical grey-white contrast

wg_lh_intensity-contrast_transversetemporal, 5, cortical grey-white contrast

wg_lh_intensity-contrast_insula, 5, cortical grey-white contrast

wg_rh_intensity-contrast_unknown, 5, cortical grey-white contrast

wg_rh_intensity-contrast_bankssts, 5, cortical grey-white contrast

wg_rh_intensity-contrast_caudalanteriorcingulate, 5, cortical grey-white contrast

wg_rh_intensity-contrast_caudalmiddlefrontal, 5, cortical grey-white contrast

wg_rh_intensity-contrast_cuneus, 5, cortical grey-white contrast

wg_rh_intensity-contrast_entorhinal, 5, cortical grey-white contrast

wg_rh_intensity-contrast_fusiform, 5, cortical grey-white contrast

wg_rh_intensity-contrast_inferiorparietal, 5, cortical grey-white contrast

wg_rh_intensity-contrast_inferiortemporal, 5, cortical grey-white contrast

wg_rh_intensity-contrast_isthmuscingulate, 5, cortical grey-white contrast

wg_rh_intensity-contrast_lateraloccipital, 5, cortical grey-white contrast

wg_rh_intensity-contrast_lateralorbitofrontal, 5, cortical grey-white contrast

wg_rh_intensity-contrast_lingual, 5, cortical grey-white contrast

wg_rh_intensity-contrast_medialorbitofrontal, 5, cortical grey-white contrast

wg_rh_intensity-contrast_middletemporal, 5, cortical grey-white contrast

wg_rh_intensity-contrast_parahippocampal, 5, cortical grey-white contrast

wg_rh_intensity-contrast_paracentral, 5, cortical grey-white contrast

wg_rh_intensity-contrast_parsopercularis, 5, cortical grey-white contrast

wg_rh_intensity-contrast_parsorbitalis, 5, cortical grey-white contrast

wg_rh_intensity-contrast_parstriangularis, 5, cortical grey-white contrast

wg_rh_intensity-contrast_pericalcarine, 5, cortical grey-white contrast

wg_rh_intensity-contrast_postcentral, 5, cortical grey-white contrast

wg_rh_intensity-contrast_posteriorcingulate, 5, cortical grey-white contrast

wg_rh_intensity-contrast_precentral, 5, cortical grey-white contrast

wg_rh_intensity-contrast_precuneus, 5, cortical grey-white contrast

wg_rh_intensity-contrast_rostralanteriorcingulate, 5, cortical grey-white contrast

wg_rh_intensity-contrast_rostralmiddlefrontal, 5, cortical grey-white contrast

wg_rh_intensity-contrast_superiorfrontal, 5, cortical grey-white contrast

wg_rh_intensity-contrast_superiorparietal, 5, cortical grey-white contrast

wg_rh_intensity-contrast_superiortemporal, 5, cortical grey-white contrast

wg_rh_intensity-contrast_supramarginal, 5, cortical grey-white contrast

wg_rh_intensity-contrast_frontalpole, 5, cortical grey-white contrast

wg_rh_intensity-contrast_temporalpole, 5, cortical grey-white contrast

wg_rh_intensity-contrast_transversetemporal, 5, cortical grey-white contrast

wg_rh_intensity-contrast_insula, 5, cortical grey-white contrast

IDP_T2_FLAIR_BIANCA_WMH_volume, 6, white matter hyperintensity volume

IDP_SWI_T2star_left_thalamus, 7, regional T2*

IDP_SWI_T2star_right_thalamus, 7, regional T2*

IDP_SWI_T2star_left_caudate, 7, regional T2*

IDP_SWI_T2star_right_caudate, 7, regional T2*

IDP_SWI_T2star_left_putamen, 7, regional T2*

IDP_SWI_T2star_right_putamen, 7, regional T2*

IDP_SWI_T2star_left_pallidum, 7, regional T2*

IDP_SWI_T2star_right_pallidum, 7, regional T2*

IDP_SWI_T2star_left_hippocampus, 7, regional T2*

IDP_SWI_T2star_right_hippocampus, 7, regional T2*

IDP_SWI_T2star_left_amygdala, 7, regional T2*

IDP_SWI_T2star_right_amygdala, 7, regional T2*

IDP_SWI_T2star_left_accumbens, 7, regional T2*

IDP_SWI_T2star_right_accumbens, 7, regional T2*

IDP_QSM_T2star_left_thalamus, 7, regional T2*

IDP_QSM_T2star_right_thalamus, 7, regional T2*

IDP_QSM_T2star_left_caudate, 7, regional T2*

IDP_QSM_T2star_right_caudate, 7, regional T2*

IDP_QSM_T2star_left_putamen, 7, regional T2*

IDP_QSM_T2star_right_putamen, 7, regional T2*

IDP_QSM_T2star_left_pallidum, 7, regional T2*

IDP_QSM_T2star_right_pallidum, 7, regional T2*

IDP_QSM_T2star_left_hippocampus, 7, regional T2*

IDP_QSM_T2star_right_hippocampus, 7, regional T2*

IDP_QSM_T2star_left_amygdala, 7, regional T2*

IDP_QSM_T2star_right_amygdala, 7, regional T2*

IDP_QSM_T2star_left_accumbens, 7, regional T2*

IDP_QSM_T2star_right_accumbens, 7, regional T2*

IDP_dMRI_TBSS_FA_Middle_cerebellar_peduncle, 8, WM tract FA

IDP_dMRI_TBSS_FA_Pontine_crossing_tract, 8, WM tract FA

IDP_dMRI_TBSS_FA_Genu_of_corpus_callosum, 8, WM tract FA

IDP_dMRI_TBSS_FA_Body_of_corpus_callosum, 8, WM tract FA

IDP_dMRI_TBSS_FA_Splenium_of_corpus_callosum, 8, WM tract FA

IDP_dMRI_TBSS_FA_Fornix, 8, WM tract FA

IDP_dMRI_TBSS_FA_Corticospinal_tract_R, 8, WM tract FA

IDP_dMRI_TBSS_FA_Corticospinal_tract_L, 8, WM tract FA

IDP_dMRI_TBSS_FA_Medial_lemniscus_R, 8, WM tract FA

IDP_dMRI_TBSS_FA_Medial_lemniscus_L, 8, WM tract FA

IDP_dMRI_TBSS_FA_Inferior_cerebellar_peduncle_R, 8, WM tract FA

IDP_dMRI_TBSS_FA_Inferior_cerebellar_peduncle_L, 8, WM tract FA

IDP_dMRI_TBSS_FA_Superior_cerebellar_peduncle_R, 8, WM tract FA

IDP_dMRI_TBSS_FA_Superior_cerebellar_peduncle_L, 8, WM tract FA

IDP_dMRI_TBSS_FA_Cerebral_peduncle_R, 8, WM tract FA

IDP_dMRI_TBSS_FA_Cerebral_peduncle_L, 8, WM tract FA

IDP_dMRI_TBSS_FA_Anterior_limb_of_internal_capsule_R, 8, WM tract FA

IDP_dMRI_TBSS_FA_Anterior_limb_of_internal_capsule_L, 8, WM tract FA

IDP_dMRI_TBSS_FA_Posterior_limb_of_internal_capsule_R, 8, WM tract FA

IDP_dMRI_TBSS_FA_Posterior_limb_of_internal_capsule_L, 8, WM tract FA

IDP_dMRI_TBSS_FA_Retrolenticular_part_of_internal_capsule_R, 8, WM tract FA

IDP_dMRI_TBSS_FA_Retrolenticular_part_of_internal_capsule_L, 8, WM tract FA

IDP_dMRI_TBSS_FA_Anterior_corona_radiata_R, 8, WM tract FA

IDP_dMRI_TBSS_FA_Anterior_corona_radiata_L, 8, WM tract FA

IDP_dMRI_TBSS_FA_Superior_corona_radiata_R, 8, WM tract FA

IDP_dMRI_TBSS_FA_Superior_corona_radiata_L, 8, WM tract FA

IDP_dMRI_TBSS_FA_Posterior_corona_radiata_R, 8, WM tract FA

IDP_dMRI_TBSS_FA_Posterior_corona_radiata_L, 8, WM tract FA

IDP_dMRI_TBSS_FA_Posterior_thalamic_radiation_R, 8, WM tract FA

IDP_dMRI_TBSS_FA_Posterior_thalamic_radiation_L, 8, WM tract FA

IDP_dMRI_TBSS_FA_Sagittal_stratum_R, 8, WM tract FA

IDP_dMRI_TBSS_FA_Sagittal_stratum_L, 8, WM tract FA

IDP_dMRI_TBSS_FA_External_capsule_R, 8, WM tract FA

IDP_dMRI_TBSS_FA_External_capsule_L, 8, WM tract FA

IDP_dMRI_TBSS_FA_Cingulum_cingulate_gyrus_R, 8, WM tract FA

IDP_dMRI_TBSS_FA_Cingulum_cingulate_gyrus_L, 8, WM tract FA

IDP_dMRI_TBSS_FA_Cingulum_hippocampus_R, 8, WM tract FA

IDP_dMRI_TBSS_FA_Cingulum_hippocampus_L, 8, WM tract FA

IDP_dMRI_TBSS_FA_Fornix_cres+Stria_terminalis_R, 8, WM tract FA

IDP_dMRI_TBSS_FA_Fornix_cres+Stria_terminalis_L, 8, WM tract FA

IDP_dMRI_TBSS_FA_Superior_longitudinal_fasciculus_R, 8, WM tract FA

IDP_dMRI_TBSS_FA_Superior_longitudinal_fasciculus_L, 8, WM tract FA

IDP_dMRI_TBSS_FA_Superior_fronto-occipital_fasciculus_R, 8, WM tract FA

IDP_dMRI_TBSS_FA_Superior_fronto-occipital_fasciculus_L, 8, WM tract FA

IDP_dMRI_TBSS_FA_Uncinate_fasciculus_R, 8, WM tract FA

IDP_dMRI_TBSS_FA_Uncinate_fasciculus_L, 8, WM tract FA

IDP_dMRI_TBSS_FA_Tapetum_R, 8, WM tract FA

IDP_dMRI_TBSS_FA_Tapetum_L, 8, WM tract FA

IDP_dMRI_ProbtrackX_FA_ar_l, 8, WM tract FA

IDP_dMRI_ProbtrackX_FA_ar_r, 8, WM tract FA

IDP_dMRI_ProbtrackX_FA_atr_l, 8, WM tract FA

IDP_dMRI_ProbtrackX_FA_atr_r, 8, WM tract FA

IDP_dMRI_ProbtrackX_FA_cgc_l, 8, WM tract FA

IDP_dMRI_ProbtrackX_FA_cgc_r, 8, WM tract FA

IDP_dMRI_ProbtrackX_FA_cgh_l, 8, WM tract FA

IDP_dMRI_ProbtrackX_FA_cgh_r, 8, WM tract FA

IDP_dMRI_ProbtrackX_FA_cst_l, 8, WM tract FA

IDP_dMRI_ProbtrackX_FA_cst_r, 8, WM tract FA

IDP_dMRI_ProbtrackX_FA_fma, 8, WM tract FA

IDP_dMRI_ProbtrackX_FA_fmi, 8, WM tract FA

IDP_dMRI_ProbtrackX_FA_ifo_l, 8, WM tract FA

IDP_dMRI_ProbtrackX_FA_ifo_r, 8, WM tract FA

IDP_dMRI_ProbtrackX_FA_ilf_l, 8, WM tract FA

IDP_dMRI_ProbtrackX_FA_ilf_r, 8, WM tract FA

IDP_dMRI_ProbtrackX_FA_mcp, 8, WM tract FA

IDP_dMRI_ProbtrackX_FA_ml_l, 8, WM tract FA

IDP_dMRI_ProbtrackX_FA_ml_r, 8, WM tract FA

IDP_dMRI_ProbtrackX_FA_ptr_l, 8, WM tract FA

IDP_dMRI_ProbtrackX_FA_ptr_r, 8, WM tract FA

IDP_dMRI_ProbtrackX_FA_slf_l, 8, WM tract FA

IDP_dMRI_ProbtrackX_FA_slf_r, 8, WM tract FA

IDP_dMRI_ProbtrackX_FA_str_l, 8, WM tract FA

IDP_dMRI_ProbtrackX_FA_str_r, 8, WM tract FA

IDP_dMRI_ProbtrackX_FA_unc_l, 8, WM tract FA

IDP_dMRI_ProbtrackX_FA_unc_r, 8, WM tract FA

IDP_dMRI_TBSS_MO_Middle_cerebellar_peduncle, 9, WM tract MO

IDP_dMRI_TBSS_MO_Pontine_crossing_tract, 9, WM tract MO

IDP_dMRI_TBSS_MO_Genu_of_corpus_callosum, 9, WM tract MO

IDP_dMRI_TBSS_MO_Body_of_corpus_callosum, 9, WM tract MO

IDP_dMRI_TBSS_MO_Splenium_of_corpus_callosum, 9, WM tract MO

IDP_dMRI_TBSS_MO_Fornix, 9, WM tract MO

IDP_dMRI_TBSS_MO_Corticospinal_tract_R, 9, WM tract MO

IDP_dMRI_TBSS_MO_Corticospinal_tract_L, 9, WM tract MO

IDP_dMRI_TBSS_MO_Medial_lemniscus_R, 9, WM tract MO

IDP_dMRI_TBSS_MO_Medial_lemniscus_L, 9, WM tract MO

IDP_dMRI_TBSS_MO_Inferior_cerebellar_peduncle_R, 9, WM tract MO

IDP_dMRI_TBSS_MO_Inferior_cerebellar_peduncle_L, 9, WM tract MO

IDP_dMRI_TBSS_MO_Superior_cerebellar_peduncle_R, 9, WM tract MO

IDP_dMRI_TBSS_MO_Superior_cerebellar_peduncle_L, 9, WM tract MO

IDP_dMRI_TBSS_MO_Cerebral_peduncle_R, 9, WM tract MO

IDP_dMRI_TBSS_MO_Cerebral_peduncle_L, 9, WM tract MO

IDP_dMRI_TBSS_MO_Anterior_limb_of_internal_capsule_R, 9, WM tract MO

IDP_dMRI_TBSS_MO_Anterior_limb_of_internal_capsule_L, 9, WM tract MO

IDP_dMRI_TBSS_MO_Posterior_limb_of_internal_capsule_R, 9, WM tract MO

IDP_dMRI_TBSS_MO_Posterior_limb_of_internal_capsule_L, 9, WM tract MO

IDP_dMRI_TBSS_MO_Retrolenticular_part_of_internal_capsule_R, 9, WM tract MO

IDP_dMRI_TBSS_MO_Retrolenticular_part_of_internal_capsule_L, 9, WM tract MO

IDP_dMRI_TBSS_MO_Anterior_corona_radiata_R, 9, WM tract MO

IDP_dMRI_TBSS_MO_Anterior_corona_radiata_L, 9, WM tract MO

IDP_dMRI_TBSS_MO_Superior_corona_radiata_R, 9, WM tract MO

IDP_dMRI_TBSS_MO_Superior_corona_radiata_L, 9, WM tract MO

IDP_dMRI_TBSS_MO_Posterior_corona_radiata_R, 9, WM tract MO

IDP_dMRI_TBSS_MO_Posterior_corona_radiata_L, 9, WM tract MO

IDP_dMRI_TBSS_MO_Posterior_thalamic_radiation_R, 9, WM tract MO

IDP_dMRI_TBSS_MO_Posterior_thalamic_radiation_L, 9, WM tract MO

IDP_dMRI_TBSS_MO_Sagittal_stratum_R, 9, WM tract MO

IDP_dMRI_TBSS_MO_Sagittal_stratum_L, 9, WM tract MO

IDP_dMRI_TBSS_MO_External_capsule_R, 9, WM tract MO

IDP_dMRI_TBSS_MO_External_capsule_L, 9, WM tract MO

IDP_dMRI_TBSS_MO_Cingulum_cingulate_gyrus_R, 9, WM tract MO

IDP_dMRI_TBSS_MO_Cingulum_cingulate_gyrus_L, 9, WM tract MO

IDP_dMRI_TBSS_MO_Cingulum_hippocampus_R, 9, WM tract MO

IDP_dMRI_TBSS_MO_Cingulum_hippocampus_L, 9, WM tract MO

IDP_dMRI_TBSS_MO_Fornix_cres+Stria_terminalis_R, 9, WM tract MO

IDP_dMRI_TBSS_MO_Fornix_cres+Stria_terminalis_L, 9, WM tract MO

IDP_dMRI_TBSS_MO_Superior_longitudinal_fasciculus_R, 9, WM tract MO

IDP_dMRI_TBSS_MO_Superior_longitudinal_fasciculus_L, 9, WM tract MO

IDP_dMRI_TBSS_MO_Superior_fronto-occipital_fasciculus_R, 9, WM tract MO

IDP_dMRI_TBSS_MO_Superior_fronto-occipital_fasciculus_L, 9, WM tract MO

IDP_dMRI_TBSS_MO_Uncinate_fasciculus_R, 9, WM tract MO

IDP_dMRI_TBSS_MO_Uncinate_fasciculus_L, 9, WM tract MO

IDP_dMRI_TBSS_MO_Tapetum_R, 9, WM tract MO

IDP_dMRI_TBSS_MO_Tapetum_L, 9, WM tract MO

IDP_dMRI_ProbtrackX_MO_ar_l, 9, WM tract MO

IDP_dMRI_ProbtrackX_MO_ar_r, 9, WM tract MO

IDP_dMRI_ProbtrackX_MO_atr_l, 9, WM tract MO

IDP_dMRI_ProbtrackX_MO_atr_r, 9, WM tract MO

IDP_dMRI_ProbtrackX_MO_cgc_l, 9, WM tract MO

IDP_dMRI_ProbtrackX_MO_cgc_r, 9, WM tract MO

IDP_dMRI_ProbtrackX_MO_cgh_l, 9, WM tract MO

IDP_dMRI_ProbtrackX_MO_cgh_r, 9, WM tract MO

IDP_dMRI_ProbtrackX_MO_cst_l, 9, WM tract MO

IDP_dMRI_ProbtrackX_MO_cst_r, 9, WM tract MO

IDP_dMRI_ProbtrackX_MO_fma, 9, WM tract MO

IDP_dMRI_ProbtrackX_MO_fmi, 9, WM tract MO

IDP_dMRI_ProbtrackX_MO_ifo_l, 9, WM tract MO

IDP_dMRI_ProbtrackX_MO_ifo_r, 9, WM tract MO

IDP_dMRI_ProbtrackX_MO_ilf_l, 9, WM tract MO

IDP_dMRI_ProbtrackX_MO_ilf_r, 9, WM tract MO

IDP_dMRI_ProbtrackX_MO_mcp, 9, WM tract MO

IDP_dMRI_ProbtrackX_MO_ml_l, 9, WM tract MO

IDP_dMRI_ProbtrackX_MO_ml_r, 9, WM tract MO

IDP_dMRI_ProbtrackX_MO_ptr_l, 9, WM tract MO

IDP_dMRI_ProbtrackX_MO_ptr_r, 9, WM tract MO

IDP_dMRI_ProbtrackX_MO_slf_l, 9, WM tract MO

IDP_dMRI_ProbtrackX_MO_slf_r, 9, WM tract MO

IDP_dMRI_ProbtrackX_MO_str_l, 9, WM tract MO

IDP_dMRI_ProbtrackX_MO_str_r, 9, WM tract MO

IDP_dMRI_ProbtrackX_MO_unc_l, 9, WM tract MO

IDP_dMRI_ProbtrackX_MO_unc_r, 9, WM tract MO

IDP_dMRI_TBSS_MD_Middle_cerebellar_peduncle, 10, WM tract diffusivity

IDP_dMRI_TBSS_MD_Pontine_crossing_tract, 10, WM tract diffusivity

IDP_dMRI_TBSS_MD_Genu_of_corpus_callosum, 10, WM tract diffusivity

IDP_dMRI_TBSS_MD_Body_of_corpus_callosum, 10, WM tract diffusivity

IDP_dMRI_TBSS_MD_Splenium_of_corpus_callosum, 10, WM tract diffusivity

IDP_dMRI_TBSS_MD_Fornix, 10, WM tract diffusivity

IDP_dMRI_TBSS_MD_Corticospinal_tract_R, 10, WM tract diffusivity

IDP_dMRI_TBSS_MD_Corticospinal_tract_L, 10, WM tract diffusivity

IDP_dMRI_TBSS_MD_Medial_lemniscus_R, 10, WM tract diffusivity

IDP_dMRI_TBSS_MD_Medial_lemniscus_L, 10, WM tract diffusivity

IDP_dMRI_TBSS_MD_Inferior_cerebellar_peduncle_R, 10, WM tract diffusivity

IDP_dMRI_TBSS_MD_Inferior_cerebellar_peduncle_L, 10, WM tract diffusivity

IDP_dMRI_TBSS_MD_Superior_cerebellar_peduncle_R, 10, WM tract diffusivity

IDP_dMRI_TBSS_MD_Superior_cerebellar_peduncle_L, 10, WM tract diffusivity

IDP_dMRI_TBSS_MD_Cerebral_peduncle_R, 10, WM tract diffusivity

IDP_dMRI_TBSS_MD_Cerebral_peduncle_L, 10, WM tract diffusivity

IDP_dMRI_TBSS_MD_Anterior_limb_of_internal_capsule_R, 10, WM tract diffusivity

IDP_dMRI_TBSS_MD_Anterior_limb_of_internal_capsule_L, 10, WM tract diffusivity

IDP_dMRI_TBSS_MD_Posterior_limb_of_internal_capsule_R, 10, WM tract diffusivity

IDP_dMRI_TBSS_MD_Posterior_limb_of_internal_capsule_L, 10, WM tract diffusivity

IDP_dMRI_TBSS_MD_Retrolenticular_part_of_internal_capsule_R, 10, WM tract diffusivity

IDP_dMRI_TBSS_MD_Retrolenticular_part_of_internal_capsule_L, 10, WM tract diffusivity

IDP_dMRI_TBSS_MD_Anterior_corona_radiata_R, 10, WM tract diffusivity

IDP_dMRI_TBSS_MD_Anterior_corona_radiata_L, 10, WM tract diffusivity

IDP_dMRI_TBSS_MD_Superior_corona_radiata_R, 10, WM tract diffusivity

IDP_dMRI_TBSS_MD_Superior_corona_radiata_L, 10, WM tract diffusivity

IDP_dMRI_TBSS_MD_Posterior_corona_radiata_R, 10, WM tract diffusivity

IDP_dMRI_TBSS_MD_Posterior_corona_radiata_L, 10, WM tract diffusivity

IDP_dMRI_TBSS_MD_Posterior_thalamic_radiation_R, 10, WM tract diffusivity

IDP_dMRI_TBSS_MD_Posterior_thalamic_radiation_L, 10, WM tract diffusivity

IDP_dMRI_TBSS_MD_Sagittal_stratum_R, 10, WM tract diffusivity

IDP_dMRI_TBSS_MD_Sagittal_stratum_L, 10, WM tract diffusivity

IDP_dMRI_TBSS_MD_External_capsule_R, 10, WM tract diffusivity

IDP_dMRI_TBSS_MD_External_capsule_L, 10, WM tract diffusivity

IDP_dMRI_TBSS_MD_Cingulum_cingulate_gyrus_R, 10, WM tract diffusivity

IDP_dMRI_TBSS_MD_Cingulum_cingulate_gyrus_L, 10, WM tract diffusivity

IDP_dMRI_TBSS_MD_Cingulum_hippocampus_R, 10, WM tract diffusivity

IDP_dMRI_TBSS_MD_Cingulum_hippocampus_L, 10, WM tract diffusivity

IDP_dMRI_TBSS_MD_Fornix_cres+Stria_terminalis_R, 10, WM tract diffusivity

IDP_dMRI_TBSS_MD_Fornix_cres+Stria_terminalis_L, 10, WM tract diffusivity

IDP_dMRI_TBSS_MD_Superior_longitudinal_fasciculus_R, 10, WM tract diffusivity

IDP_dMRI_TBSS_MD_Superior_longitudinal_fasciculus_L, 10, WM tract diffusivity

IDP_dMRI_TBSS_MD_Superior_fronto-occipital_fasciculus_R, 10, WM tract diffusivity

IDP_dMRI_TBSS_MD_Superior_fronto-occipital_fasciculus_L, 10, WM tract diffusivity

IDP_dMRI_TBSS_MD_Uncinate_fasciculus_R, 10, WM tract diffusivity

IDP_dMRI_TBSS_MD_Uncinate_fasciculus_L, 10, WM tract diffusivity

IDP_dMRI_TBSS_MD_Tapetum_R, 10, WM tract diffusivity

IDP_dMRI_TBSS_MD_Tapetum_L, 10, WM tract diffusivity

IDP_dMRI_TBSS_L1_Middle_cerebellar_peduncle, 10, WM tract diffusivity

IDP_dMRI_TBSS_L1_Pontine_crossing_tract, 10, WM tract diffusivity

IDP_dMRI_TBSS_L1_Genu_of_corpus_callosum, 10, WM tract diffusivity

IDP_dMRI_TBSS_L1_Body_of_corpus_callosum, 10, WM tract diffusivity

IDP_dMRI_TBSS_L1_Splenium_of_corpus_callosum, 10, WM tract diffusivity

IDP_dMRI_TBSS_L1_Fornix, 10, WM tract diffusivity

IDP_dMRI_TBSS_L1_Corticospinal_tract_R, 10, WM tract diffusivity

IDP_dMRI_TBSS_L1_Corticospinal_tract_L, 10, WM tract diffusivity

IDP_dMRI_TBSS_L1_Medial_lemniscus_R, 10, WM tract diffusivity

IDP_dMRI_TBSS_L1_Medial_lemniscus_L, 10, WM tract diffusivity

IDP_dMRI_TBSS_L1_Inferior_cerebellar_peduncle_R, 10, WM tract diffusivity

IDP_dMRI_TBSS_L1_Inferior_cerebellar_peduncle_L, 10, WM tract diffusivity

IDP_dMRI_TBSS_L1_Superior_cerebellar_peduncle_R, 10, WM tract diffusivity

IDP_dMRI_TBSS_L1_Superior_cerebellar_peduncle_L, 10, WM tract diffusivity

IDP_dMRI_TBSS_L1_Cerebral_peduncle_R, 10, WM tract diffusivity

IDP_dMRI_TBSS_L1_Cerebral_peduncle_L, 10, WM tract diffusivity

IDP_dMRI_TBSS_L1_Anterior_limb_of_internal_capsule_R, 10, WM tract diffusivity

IDP_dMRI_TBSS_L1_Anterior_limb_of_internal_capsule_L, 10, WM tract diffusivity

IDP_dMRI_TBSS_L1_Posterior_limb_of_internal_capsule_R, 10, WM tract diffusivity

IDP_dMRI_TBSS_L1_Posterior_limb_of_internal_capsule_L, 10, WM tract diffusivity

IDP_dMRI_TBSS_L1_Retrolenticular_part_of_internal_capsule_R, 10, WM tract diffusivity

IDP_dMRI_TBSS_L1_Retrolenticular_part_of_internal_capsule_L, 10, WM tract diffusivity

IDP_dMRI_TBSS_L1_Anterior_corona_radiata_R, 10, WM tract diffusivity

IDP_dMRI_TBSS_L1_Anterior_corona_radiata_L, 10, WM tract diffusivity

IDP_dMRI_TBSS_L1_Superior_corona_radiata_R, 10, WM tract diffusivity

IDP_dMRI_TBSS_L1_Superior_corona_radiata_L, 10, WM tract diffusivity

IDP_dMRI_TBSS_L1_Posterior_corona_radiata_R, 10, WM tract diffusivity

IDP_dMRI_TBSS_L1_Posterior_corona_radiata_L, 10, WM tract diffusivity

IDP_dMRI_TBSS_L1_Posterior_thalamic_radiation_R, 10, WM tract diffusivity

IDP_dMRI_TBSS_L1_Posterior_thalamic_radiation_L, 10, WM tract diffusivity

IDP_dMRI_TBSS_L1_Sagittal_stratum_R, 10, WM tract diffusivity

IDP_dMRI_TBSS_L1_Sagittal_stratum_L, 10, WM tract diffusivity

IDP_dMRI_TBSS_L1_External_capsule_R, 10, WM tract diffusivity

IDP_dMRI_TBSS_L1_External_capsule_L, 10, WM tract diffusivity

IDP_dMRI_TBSS_L1_Cingulum_cingulate_gyrus_R, 10, WM tract diffusivity

IDP_dMRI_TBSS_L1_Cingulum_cingulate_gyrus_L, 10, WM tract diffusivity

IDP_dMRI_TBSS_L1_Cingulum_hippocampus_R, 10, WM tract diffusivity

IDP_dMRI_TBSS_L1_Cingulum_hippocampus_L, 10, WM tract diffusivity

IDP_dMRI_TBSS_L1_Fornix_cres+Stria_terminalis_R, 10, WM tract diffusivity

IDP_dMRI_TBSS_L1_Fornix_cres+Stria_terminalis_L, 10, WM tract diffusivity

IDP_dMRI_TBSS_L1_Superior_longitudinal_fasciculus_R, 10, WM tract diffusivity

IDP_dMRI_TBSS_L1_Superior_longitudinal_fasciculus_L, 10, WM tract diffusivity

IDP_dMRI_TBSS_L1_Superior_fronto-occipital_fasciculus_R, 10, WM tract diffusivity

IDP_dMRI_TBSS_L1_Superior_fronto-occipital_fasciculus_L, 10, WM tract diffusivity

IDP_dMRI_TBSS_L1_Uncinate_fasciculus_R, 10, WM tract diffusivity

IDP_dMRI_TBSS_L1_Uncinate_fasciculus_L, 10, WM tract diffusivity

IDP_dMRI_TBSS_L1_Tapetum_R, 10, WM tract diffusivity

IDP_dMRI_TBSS_L1_Tapetum_L, 10, WM tract diffusivity

IDP_dMRI_TBSS_L2_Middle_cerebellar_peduncle, 10, WM tract diffusivity

IDP_dMRI_TBSS_L2_Pontine_crossing_tract, 10, WM tract diffusivity

IDP_dMRI_TBSS_L2_Genu_of_corpus_callosum, 10, WM tract diffusivity

IDP_dMRI_TBSS_L2_Body_of_corpus_callosum, 10, WM tract diffusivity

IDP_dMRI_TBSS_L2_Splenium_of_corpus_callosum, 10, WM tract diffusivity

IDP_dMRI_TBSS_L2_Fornix, 10, WM tract diffusivity

IDP_dMRI_TBSS_L2_Corticospinal_tract_R, 10, WM tract diffusivity

IDP_dMRI_TBSS_L2_Corticospinal_tract_L, 10, WM tract diffusivity

IDP_dMRI_TBSS_L2_Medial_lemniscus_R, 10, WM tract diffusivity

IDP_dMRI_TBSS_L2_Medial_lemniscus_L, 10, WM tract diffusivity

IDP_dMRI_TBSS_L2_Inferior_cerebellar_peduncle_R, 10, WM tract diffusivity

IDP_dMRI_TBSS_L2_Inferior_cerebellar_peduncle_L, 10, WM tract diffusivity

IDP_dMRI_TBSS_L2_Superior_cerebellar_peduncle_R, 10, WM tract diffusivity

IDP_dMRI_TBSS_L2_Superior_cerebellar_peduncle_L, 10, WM tract diffusivity

IDP_dMRI_TBSS_L2_Cerebral_peduncle_R, 10, WM tract diffusivity

IDP_dMRI_TBSS_L2_Cerebral_peduncle_L, 10, WM tract diffusivity

IDP_dMRI_TBSS_L2_Anterior_limb_of_internal_capsule_R, 10, WM tract diffusivity

IDP_dMRI_TBSS_L2_Anterior_limb_of_internal_capsule_L, 10, WM tract diffusivity

IDP_dMRI_TBSS_L2_Posterior_limb_of_internal_capsule_R, 10, WM tract diffusivity

IDP_dMRI_TBSS_L2_Posterior_limb_of_internal_capsule_L, 10, WM tract diffusivity

IDP_dMRI_TBSS_L2_Retrolenticular_part_of_internal_capsule_R, 10, WM tract diffusivity

IDP_dMRI_TBSS_L2_Retrolenticular_part_of_internal_capsule_L, 10, WM tract diffusivity

IDP_dMRI_TBSS_L2_Anterior_corona_radiata_R, 10, WM tract diffusivity

IDP_dMRI_TBSS_L2_Anterior_corona_radiata_L, 10, WM tract diffusivity

IDP_dMRI_TBSS_L2_Superior_corona_radiata_R, 10, WM tract diffusivity

IDP_dMRI_TBSS_L2_Superior_corona_radiata_L, 10, WM tract diffusivity

IDP_dMRI_TBSS_L2_Posterior_corona_radiata_R, 10, WM tract diffusivity

IDP_dMRI_TBSS_L2_Posterior_corona_radiata_L, 10, WM tract diffusivity

IDP_dMRI_TBSS_L2_Posterior_thalamic_radiation_R, 10, WM tract diffusivity

IDP_dMRI_TBSS_L2_Posterior_thalamic_radiation_L, 10, WM tract diffusivity

IDP_dMRI_TBSS_L2_Sagittal_stratum_R, 10, WM tract diffusivity

IDP_dMRI_TBSS_L2_Sagittal_stratum_L, 10, WM tract diffusivity

IDP_dMRI_TBSS_L2_External_capsule_R, 10, WM tract diffusivity

IDP_dMRI_TBSS_L2_External_capsule_L, 10, WM tract diffusivity

IDP_dMRI_TBSS_L2_Cingulum_cingulate_gyrus_R, 10, WM tract diffusivity

IDP_dMRI_TBSS_L2_Cingulum_cingulate_gyrus_L, 10, WM tract diffusivity

IDP_dMRI_TBSS_L2_Cingulum_hippocampus_R, 10, WM tract diffusivity

IDP_dMRI_TBSS_L2_Cingulum_hippocampus_L, 10, WM tract diffusivity

IDP_dMRI_TBSS_L2_Fornix_cres+Stria_terminalis_R, 10, WM tract diffusivity

IDP_dMRI_TBSS_L2_Fornix_cres+Stria_terminalis_L, 10, WM tract diffusivity

IDP_dMRI_TBSS_L2_Superior_longitudinal_fasciculus_R, 10, WM tract diffusivity

IDP_dMRI_TBSS_L2_Superior_longitudinal_fasciculus_L, 10, WM tract diffusivity

IDP_dMRI_TBSS_L2_Superior_fronto-occipital_fasciculus_R, 10, WM tract diffusivity

IDP_dMRI_TBSS_L2_Superior_fronto-occipital_fasciculus_L, 10, WM tract diffusivity

IDP_dMRI_TBSS_L2_Uncinate_fasciculus_R, 10, WM tract diffusivity

IDP_dMRI_TBSS_L2_Uncinate_fasciculus_L, 10, WM tract diffusivity

IDP_dMRI_TBSS_L2_Tapetum_R, 10, WM tract diffusivity

IDP_dMRI_TBSS_L2_Tapetum_L, 10, WM tract diffusivity

IDP_dMRI_TBSS_L3_Middle_cerebellar_peduncle, 10, WM tract diffusivity

IDP_dMRI_TBSS_L3_Pontine_crossing_tract, 10, WM tract diffusivity

IDP_dMRI_TBSS_L3_Genu_of_corpus_callosum, 10, WM tract diffusivity

IDP_dMRI_TBSS_L3_Body_of_corpus_callosum, 10, WM tract diffusivity

IDP_dMRI_TBSS_L3_Splenium_of_corpus_callosum, 10, WM tract diffusivity

IDP_dMRI_TBSS_L3_Fornix, 10, WM tract diffusivity

IDP_dMRI_TBSS_L3_Corticospinal_tract_R, 10, WM tract diffusivity

IDP_dMRI_TBSS_L3_Corticospinal_tract_L, 10, WM tract diffusivity

IDP_dMRI_TBSS_L3_Medial_lemniscus_R, 10, WM tract diffusivity

IDP_dMRI_TBSS_L3_Medial_lemniscus_L, 10, WM tract diffusivity

IDP_dMRI_TBSS_L3_Inferior_cerebellar_peduncle_R, 10, WM tract diffusivity

IDP_dMRI_TBSS_L3_Inferior_cerebellar_peduncle_L, 10, WM tract diffusivity

IDP_dMRI_TBSS_L3_Superior_cerebellar_peduncle_R, 10, WM tract diffusivity

IDP_dMRI_TBSS_L3_Superior_cerebellar_peduncle_L, 10, WM tract diffusivity

IDP_dMRI_TBSS_L3_Cerebral_peduncle_R, 10, WM tract diffusivity

IDP_dMRI_TBSS_L3_Cerebral_peduncle_L, 10, WM tract diffusivity

IDP_dMRI_TBSS_L3_Anterior_limb_of_internal_capsule_R, 10, WM tract diffusivity

IDP_dMRI_TBSS_L3_Anterior_limb_of_internal_capsule_L, 10, WM tract diffusivity

IDP_dMRI_TBSS_L3_Posterior_limb_of_internal_capsule_R, 10, WM tract diffusivity

IDP_dMRI_TBSS_L3_Posterior_limb_of_internal_capsule_L, 10, WM tract diffusivity

IDP_dMRI_TBSS_L3_Retrolenticular_part_of_internal_capsule_R, 10, WM tract diffusivity

IDP_dMRI_TBSS_L3_Retrolenticular_part_of_internal_capsule_L, 10, WM tract diffusivity

IDP_dMRI_TBSS_L3_Anterior_corona_radiata_R, 10, WM tract diffusivity

IDP_dMRI_TBSS_L3_Anterior_corona_radiata_L, 10, WM tract diffusivity

IDP_dMRI_TBSS_L3_Superior_corona_radiata_R, 10, WM tract diffusivity

IDP_dMRI_TBSS_L3_Superior_corona_radiata_L, 10, WM tract diffusivity

IDP_dMRI_TBSS_L3_Posterior_corona_radiata_R, 10, WM tract diffusivity

IDP_dMRI_TBSS_L3_Posterior_corona_radiata_L, 10, WM tract diffusivity

IDP_dMRI_TBSS_L3_Posterior_thalamic_radiation_R, 10, WM tract diffusivity

IDP_dMRI_TBSS_L3_Posterior_thalamic_radiation_L, 10, WM tract diffusivity

IDP_dMRI_TBSS_L3_Sagittal_stratum_R, 10, WM tract diffusivity

IDP_dMRI_TBSS_L3_Sagittal_stratum_L, 10, WM tract diffusivity

IDP_dMRI_TBSS_L3_External_capsule_R, 10, WM tract diffusivity

IDP_dMRI_TBSS_L3_External_capsule_L, 10, WM tract diffusivity

IDP_dMRI_TBSS_L3_Cingulum_cingulate_gyrus_R, 10, WM tract diffusivity

IDP_dMRI_TBSS_L3_Cingulum_cingulate_gyrus_L, 10, WM tract diffusivity

IDP_dMRI_TBSS_L3_Cingulum_hippocampus_R, 10, WM tract diffusivity

IDP_dMRI_TBSS_L3_Cingulum_hippocampus_L, 10, WM tract diffusivity

IDP_dMRI_TBSS_L3_Fornix_cres+Stria_terminalis_R, 10, WM tract diffusivity

IDP_dMRI_TBSS_L3_Fornix_cres+Stria_terminalis_L, 10, WM tract diffusivity

IDP_dMRI_TBSS_L3_Superior_longitudinal_fasciculus_R, 10, WM tract diffusivity

IDP_dMRI_TBSS_L3_Superior_longitudinal_fasciculus_L, 10, WM tract diffusivity

IDP_dMRI_TBSS_L3_Superior_fronto-occipital_fasciculus_R, 10, WM tract diffusivity

IDP_dMRI_TBSS_L3_Superior_fronto-occipital_fasciculus_L, 10, WM tract diffusivity

IDP_dMRI_TBSS_L3_Uncinate_fasciculus_R, 10, WM tract diffusivity

IDP_dMRI_TBSS_L3_Uncinate_fasciculus_L, 10, WM tract diffusivity

IDP_dMRI_TBSS_L3_Tapetum_R, 10, WM tract diffusivity

IDP_dMRI_TBSS_L3_Tapetum_L, 10, WM tract diffusivity

IDP_dMRI_ProbtrackX_MD_ar_l, 10, WM tract diffusivity

IDP_dMRI_ProbtrackX_MD_ar_r, 10, WM tract diffusivity

IDP_dMRI_ProbtrackX_MD_atr_l, 10, WM tract diffusivity

IDP_dMRI_ProbtrackX_MD_atr_r, 10, WM tract diffusivity

IDP_dMRI_ProbtrackX_MD_cgc_l, 10, WM tract diffusivity

IDP_dMRI_ProbtrackX_MD_cgc_r, 10, WM tract diffusivity

IDP_dMRI_ProbtrackX_MD_cgh_l, 10, WM tract diffusivity

IDP_dMRI_ProbtrackX_MD_cgh_r, 10, WM tract diffusivity

IDP_dMRI_ProbtrackX_MD_cst_l, 10, WM tract diffusivity

IDP_dMRI_ProbtrackX_MD_cst_r, 10, WM tract diffusivity

IDP_dMRI_ProbtrackX_MD_fma, 10, WM tract diffusivity

IDP_dMRI_ProbtrackX_MD_fmi, 10, WM tract diffusivity

IDP_dMRI_ProbtrackX_MD_ifo_l, 10, WM tract diffusivity

IDP_dMRI_ProbtrackX_MD_ifo_r, 10, WM tract diffusivity

IDP_dMRI_ProbtrackX_MD_ilf_l, 10, WM tract diffusivity

IDP_dMRI_ProbtrackX_MD_ilf_r, 10, WM tract diffusivity

IDP_dMRI_ProbtrackX_MD_mcp, 10, WM tract diffusivity

IDP_dMRI_ProbtrackX_MD_ml_l, 10, WM tract diffusivity

IDP_dMRI_ProbtrackX_MD_ml_r, 10, WM tract diffusivity

IDP_dMRI_ProbtrackX_MD_ptr_l, 10, WM tract diffusivity

IDP_dMRI_ProbtrackX_MD_ptr_r, 10, WM tract diffusivity

IDP_dMRI_ProbtrackX_MD_slf_l, 10, WM tract diffusivity

IDP_dMRI_ProbtrackX_MD_slf_r, 10, WM tract diffusivity

IDP_dMRI_ProbtrackX_MD_str_l, 10, WM tract diffusivity

IDP_dMRI_ProbtrackX_MD_str_r, 10, WM tract diffusivity

IDP_dMRI_ProbtrackX_MD_unc_l, 10, WM tract diffusivity

IDP_dMRI_ProbtrackX_MD_unc_r, 10, WM tract diffusivity

IDP_dMRI_ProbtrackX_L1_ar_l, 10, WM tract diffusivity

IDP_dMRI_ProbtrackX_L1_ar_r, 10, WM tract diffusivity

IDP_dMRI_ProbtrackX_L1_atr_l, 10, WM tract diffusivity

IDP_dMRI_ProbtrackX_L1_atr_r, 10, WM tract diffusivity

IDP_dMRI_ProbtrackX_L1_cgc_l, 10, WM tract diffusivity

IDP_dMRI_ProbtrackX_L1_cgc_r, 10, WM tract diffusivity

IDP_dMRI_ProbtrackX_L1_cgh_l, 10, WM tract diffusivity

IDP_dMRI_ProbtrackX_L1_cgh_r, 10, WM tract diffusivity

IDP_dMRI_ProbtrackX_L1_cst_l, 10, WM tract diffusivity

IDP_dMRI_ProbtrackX_L1_cst_r, 10, WM tract diffusivity

IDP_dMRI_ProbtrackX_L1_fma, 10, WM tract diffusivity

IDP_dMRI_ProbtrackX_L1_fmi, 10, WM tract diffusivity

IDP_dMRI_ProbtrackX_L1_ifo_l, 10, WM tract diffusivity

IDP_dMRI_ProbtrackX_L1_ifo_r, 10, WM tract diffusivity

IDP_dMRI_ProbtrackX_L1_ilf_l, 10, WM tract diffusivity

IDP_dMRI_ProbtrackX_L1_ilf_r, 10, WM tract diffusivity

IDP_dMRI_ProbtrackX_L1_mcp, 10, WM tract diffusivity

IDP_dMRI_ProbtrackX_L1_ml_l, 10, WM tract diffusivity

IDP_dMRI_ProbtrackX_L1_ml_r, 10, WM tract diffusivity

IDP_dMRI_ProbtrackX_L1_ptr_l, 10, WM tract diffusivity

IDP_dMRI_ProbtrackX_L1_ptr_r, 10, WM tract diffusivity

IDP_dMRI_ProbtrackX_L1_slf_l, 10, WM tract diffusivity

IDP_dMRI_ProbtrackX_L1_slf_r, 10, WM tract diffusivity

IDP_dMRI_ProbtrackX_L1_str_l, 10, WM tract diffusivity

IDP_dMRI_ProbtrackX_L1_str_r, 10, WM tract diffusivity

IDP_dMRI_ProbtrackX_L1_unc_l, 10, WM tract diffusivity

IDP_dMRI_ProbtrackX_L1_unc_r, 10, WM tract diffusivity

IDP_dMRI_ProbtrackX_L2_ar_l, 10, WM tract diffusivity

IDP_dMRI_ProbtrackX_L2_ar_r, 10, WM tract diffusivity

IDP_dMRI_ProbtrackX_L2_atr_l, 10, WM tract diffusivity

IDP_dMRI_ProbtrackX_L2_atr_r, 10, WM tract diffusivity

IDP_dMRI_ProbtrackX_L2_cgc_l, 10, WM tract diffusivity

IDP_dMRI_ProbtrackX_L2_cgc_r, 10, WM tract diffusivity

IDP_dMRI_ProbtrackX_L2_cgh_l, 10, WM tract diffusivity

IDP_dMRI_ProbtrackX_L2_cgh_r, 10, WM tract diffusivity

IDP_dMRI_ProbtrackX_L2_cst_l, 10, WM tract diffusivity

IDP_dMRI_ProbtrackX_L2_cst_r, 10, WM tract diffusivity

IDP_dMRI_ProbtrackX_L2_fma, 10, WM tract diffusivity

IDP_dMRI_ProbtrackX_L2_fmi, 10, WM tract diffusivity

IDP_dMRI_ProbtrackX_L2_ifo_l, 10, WM tract diffusivity

IDP_dMRI_ProbtrackX_L2_ifo_r, 10, WM tract diffusivity

IDP_dMRI_ProbtrackX_L2_ilf_l, 10, WM tract diffusivity

IDP_dMRI_ProbtrackX_L2_ilf_r, 10, WM tract diffusivity

IDP_dMRI_ProbtrackX_L2_mcp, 10, WM tract diffusivity

IDP_dMRI_ProbtrackX_L2_ml_l, 10, WM tract diffusivity

IDP_dMRI_ProbtrackX_L2_ml_r, 10, WM tract diffusivity

IDP_dMRI_ProbtrackX_L2_ptr_l, 10, WM tract diffusivity

IDP_dMRI_ProbtrackX_L2_ptr_r, 10, WM tract diffusivity

IDP_dMRI_ProbtrackX_L2_slf_l, 10, WM tract diffusivity

IDP_dMRI_ProbtrackX_L2_slf_r, 10, WM tract diffusivity

IDP_dMRI_ProbtrackX_L2_str_l, 10, WM tract diffusivity

IDP_dMRI_ProbtrackX_L2_str_r, 10, WM tract diffusivity

IDP_dMRI_ProbtrackX_L2_unc_l, 10, WM tract diffusivity

IDP_dMRI_ProbtrackX_L2_unc_r, 10, WM tract diffusivity

IDP_dMRI_ProbtrackX_L3_ar_l, 10, WM tract diffusivity

IDP_dMRI_ProbtrackX_L3_ar_r, 10, WM tract diffusivity

IDP_dMRI_ProbtrackX_L3_atr_l, 10, WM tract diffusivity

IDP_dMRI_ProbtrackX_L3_atr_r, 10, WM tract diffusivity

IDP_dMRI_ProbtrackX_L3_cgc_l, 10, WM tract diffusivity

IDP_dMRI_ProbtrackX_L3_cgc_r, 10, WM tract diffusivity

IDP_dMRI_ProbtrackX_L3_cgh_l, 10, WM tract diffusivity

IDP_dMRI_ProbtrackX_L3_cgh_r, 10, WM tract diffusivity

IDP_dMRI_ProbtrackX_L3_cst_l, 10, WM tract diffusivity

IDP_dMRI_ProbtrackX_L3_cst_r, 10, WM tract diffusivity

IDP_dMRI_ProbtrackX_L3_fma, 10, WM tract diffusivity

IDP_dMRI_ProbtrackX_L3_fmi, 10, WM tract diffusivity

IDP_dMRI_ProbtrackX_L3_ifo_l, 10, WM tract diffusivity

IDP_dMRI_ProbtrackX_L3_ifo_r, 10, WM tract diffusivity

IDP_dMRI_ProbtrackX_L3_ilf_l, 10, WM tract diffusivity

IDP_dMRI_ProbtrackX_L3_ilf_r, 10, WM tract diffusivity

IDP_dMRI_ProbtrackX_L3_mcp, 10, WM tract diffusivity

IDP_dMRI_ProbtrackX_L3_ml_l, 10, WM tract diffusivity

IDP_dMRI_ProbtrackX_L3_ml_r, 10, WM tract diffusivity

IDP_dMRI_ProbtrackX_L3_ptr_l, 10, WM tract diffusivity

IDP_dMRI_ProbtrackX_L3_ptr_r, 10, WM tract diffusivity

IDP_dMRI_ProbtrackX_L3_slf_l, 10, WM tract diffusivity

IDP_dMRI_ProbtrackX_L3_slf_r, 10, WM tract diffusivity

IDP_dMRI_ProbtrackX_L3_str_l, 10, WM tract diffusivity

IDP_dMRI_ProbtrackX_L3_str_r, 10, WM tract diffusivity

IDP_dMRI_ProbtrackX_L3_unc_l, 10, WM tract diffusivity

IDP_dMRI_ProbtrackX_L3_unc_r, 10, WM tract diffusivity

IDP_dMRI_TBSS_ICVF_Middle_cerebellar_peduncle, 11, WM tract ICVF

IDP_dMRI_TBSS_ICVF_Pontine_crossing_tract, 11, WM tract ICVF

IDP_dMRI_TBSS_ICVF_Genu_of_corpus_callosum, 11, WM tract ICVF

IDP_dMRI_TBSS_ICVF_Body_of_corpus_callosum, 11, WM tract ICVF

IDP_dMRI_TBSS_ICVF_Splenium_of_corpus_callosum, 11, WM tract ICVF

IDP_dMRI_TBSS_ICVF_Fornix, 11, WM tract ICVF

IDP_dMRI_TBSS_ICVF_Corticospinal_tract_R, 11, WM tract ICVF

IDP_dMRI_TBSS_ICVF_Corticospinal_tract_L, 11, WM tract ICVF

IDP_dMRI_TBSS_ICVF_Medial_lemniscus_R, 11, WM tract ICVF

IDP_dMRI_TBSS_ICVF_Medial_lemniscus_L, 11, WM tract ICVF

IDP_dMRI_TBSS_ICVF_Inferior_cerebellar_peduncle_R, 11, WM tract ICVF

IDP_dMRI_TBSS_ICVF_Inferior_cerebellar_peduncle_L, 11, WM tract ICVF

IDP_dMRI_TBSS_ICVF_Superior_cerebellar_peduncle_R, 11, WM tract ICVF

IDP_dMRI_TBSS_ICVF_Superior_cerebellar_peduncle_L, 11, WM tract ICVF

IDP_dMRI_TBSS_ICVF_Cerebral_peduncle_R, 11, WM tract ICVF

IDP_dMRI_TBSS_ICVF_Cerebral_peduncle_L, 11, WM tract ICVF

IDP_dMRI_TBSS_ICVF_Anterior_limb_of_internal_capsule_R, 11, WM tract ICVF

IDP_dMRI_TBSS_ICVF_Anterior_limb_of_internal_capsule_L, 11, WM tract ICVF

IDP_dMRI_TBSS_ICVF_Posterior_limb_of_internal_capsule_R, 11, WM tract ICVF

IDP_dMRI_TBSS_ICVF_Posterior_limb_of_internal_capsule_L, 11, WM tract ICVF

IDP_dMRI_TBSS_ICVF_Retrolenticular_part_of_internal_capsule_R, 11, WM tract ICVF

IDP_dMRI_TBSS_ICVF_Retrolenticular_part_of_internal_capsule_L, 11, WM tract ICVF

IDP_dMRI_TBSS_ICVF_Anterior_corona_radiata_R, 11, WM tract ICVF

IDP_dMRI_TBSS_ICVF_Anterior_corona_radiata_L, 11, WM tract ICVF

IDP_dMRI_TBSS_ICVF_Superior_corona_radiata_R, 11, WM tract ICVF

IDP_dMRI_TBSS_ICVF_Superior_corona_radiata_L, 11, WM tract ICVF

IDP_dMRI_TBSS_ICVF_Posterior_corona_radiata_R, 11, WM tract ICVF

IDP_dMRI_TBSS_ICVF_Posterior_corona_radiata_L, 11, WM tract ICVF

IDP_dMRI_TBSS_ICVF_Posterior_thalamic_radiation_R, 11, WM tract ICVF

IDP_dMRI_TBSS_ICVF_Posterior_thalamic_radiation_L, 11, WM tract ICVF

IDP_dMRI_TBSS_ICVF_Sagittal_stratum_R, 11, WM tract ICVF

IDP_dMRI_TBSS_ICVF_Sagittal_stratum_L, 11, WM tract ICVF

IDP_dMRI_TBSS_ICVF_External_capsule_R, 11, WM tract ICVF

IDP_dMRI_TBSS_ICVF_External_capsule_L, 11, WM tract ICVF

IDP_dMRI_TBSS_ICVF_Cingulum_cingulate_gyrus_R, 11, WM tract ICVF

IDP_dMRI_TBSS_ICVF_Cingulum_cingulate_gyrus_L, 11, WM tract ICVF

IDP_dMRI_TBSS_ICVF_Cingulum_hippocampus_R, 11, WM tract ICVF

IDP_dMRI_TBSS_ICVF_Cingulum_hippocampus_L, 11, WM tract ICVF

IDP_dMRI_TBSS_ICVF_Fornix_cres+Stria_terminalis_R, 11, WM tract ICVF

IDP_dMRI_TBSS_ICVF_Fornix_cres+Stria_terminalis_L, 11, WM tract ICVF

IDP_dMRI_TBSS_ICVF_Superior_longitudinal_fasciculus_R, 11, WM tract ICVF

IDP_dMRI_TBSS_ICVF_Superior_longitudinal_fasciculus_L, 11, WM tract ICVF

IDP_dMRI_TBSS_ICVF_Superior_fronto-occipital_fasciculus_R, 11, WM tract ICVF

IDP_dMRI_TBSS_ICVF_Superior_fronto-occipital_fasciculus_L, 11, WM tract ICVF

IDP_dMRI_TBSS_ICVF_Uncinate_fasciculus_R, 11, WM tract ICVF

IDP_dMRI_TBSS_ICVF_Uncinate_fasciculus_L, 11, WM tract ICVF

IDP_dMRI_TBSS_ICVF_Tapetum_R, 11, WM tract ICVF

IDP_dMRI_TBSS_ICVF_Tapetum_L, 11, WM tract ICVF

IDP_dMRI_ProbtrackX_ICVF_ar_l, 11, WM tract ICVF

IDP_dMRI_ProbtrackX_ICVF_ar_r, 11, WM tract ICVF

IDP_dMRI_ProbtrackX_ICVF_atr_l, 11, WM tract ICVF

IDP_dMRI_ProbtrackX_ICVF_atr_r, 11, WM tract ICVF

IDP_dMRI_ProbtrackX_ICVF_cgc_l, 11, WM tract ICVF

IDP_dMRI_ProbtrackX_ICVF_cgc_r, 11, WM tract ICVF

IDP_dMRI_ProbtrackX_ICVF_cgh_l, 11, WM tract ICVF

IDP_dMRI_ProbtrackX_ICVF_cgh_r, 11, WM tract ICVF

IDP_dMRI_ProbtrackX_ICVF_cst_l, 11, WM tract ICVF

IDP_dMRI_ProbtrackX_ICVF_cst_r, 11, WM tract ICVF

IDP_dMRI_ProbtrackX_ICVF_fma, 11, WM tract ICVF

IDP_dMRI_ProbtrackX_ICVF_fmi, 11, WM tract ICVF

IDP_dMRI_ProbtrackX_ICVF_ifo_l, 11, WM tract ICVF

IDP_dMRI_ProbtrackX_ICVF_ifo_r, 11, WM tract ICVF

IDP_dMRI_ProbtrackX_ICVF_ilf_l, 11, WM tract ICVF

IDP_dMRI_ProbtrackX_ICVF_ilf_r, 11, WM tract ICVF

IDP_dMRI_ProbtrackX_ICVF_mcp, 11, WM tract ICVF

IDP_dMRI_ProbtrackX_ICVF_ml_l, 11, WM tract ICVF

IDP_dMRI_ProbtrackX_ICVF_ml_r, 11, WM tract ICVF

IDP_dMRI_ProbtrackX_ICVF_ptr_l, 11, WM tract ICVF

IDP_dMRI_ProbtrackX_ICVF_ptr_r, 11, WM tract ICVF

IDP_dMRI_ProbtrackX_ICVF_slf_l, 11, WM tract ICVF

IDP_dMRI_ProbtrackX_ICVF_slf_r, 11, WM tract ICVF

IDP_dMRI_ProbtrackX_ICVF_str_l, 11, WM tract ICVF

IDP_dMRI_ProbtrackX_ICVF_str_r, 11, WM tract ICVF

IDP_dMRI_ProbtrackX_ICVF_unc_l, 11, WM tract ICVF

IDP_dMRI_ProbtrackX_ICVF_unc_r, 11, WM tract ICVF

IDP_dMRI_TBSS_OD_Middle_cerebellar_peduncle, 12, WM tract OD

IDP_dMRI_TBSS_OD_Pontine_crossing_tract, 12, WM tract OD

IDP_dMRI_TBSS_OD_Genu_of_corpus_callosum, 12, WM tract OD

IDP_dMRI_TBSS_OD_Body_of_corpus_callosum, 12, WM tract OD

IDP_dMRI_TBSS_OD_Splenium_of_corpus_callosum, 12, WM tract OD

IDP_dMRI_TBSS_OD_Fornix, 12, WM tract OD

IDP_dMRI_TBSS_OD_Corticospinal_tract_R, 12, WM tract OD

IDP_dMRI_TBSS_OD_Corticospinal_tract_L, 12, WM tract OD

IDP_dMRI_TBSS_OD_Medial_lemniscus_R, 12, WM tract OD

IDP_dMRI_TBSS_OD_Medial_lemniscus_L, 12, WM tract OD

IDP_dMRI_TBSS_OD_Inferior_cerebellar_peduncle_R, 12, WM tract OD

IDP_dMRI_TBSS_OD_Inferior_cerebellar_peduncle_L, 12, WM tract OD

IDP_dMRI_TBSS_OD_Superior_cerebellar_peduncle_R, 12, WM tract OD

IDP_dMRI_TBSS_OD_Superior_cerebellar_peduncle_L, 12, WM tract OD

IDP_dMRI_TBSS_OD_Cerebral_peduncle_R, 12, WM tract OD

IDP_dMRI_TBSS_OD_Cerebral_peduncle_L, 12, WM tract OD

IDP_dMRI_TBSS_OD_Anterior_limb_of_internal_capsule_R, 12, WM tract OD

IDP_dMRI_TBSS_OD_Anterior_limb_of_internal_capsule_L, 12, WM tract OD

IDP_dMRI_TBSS_OD_Posterior_limb_of_internal_capsule_R, 12, WM tract OD

IDP_dMRI_TBSS_OD_Posterior_limb_of_internal_capsule_L, 12, WM tract OD

IDP_dMRI_TBSS_OD_Retrolenticular_part_of_internal_capsule_R, 12, WM tract OD

IDP_dMRI_TBSS_OD_Retrolenticular_part_of_internal_capsule_L, 12, WM tract OD

IDP_dMRI_TBSS_OD_Anterior_corona_radiata_R, 12, WM tract OD

IDP_dMRI_TBSS_OD_Anterior_corona_radiata_L, 12, WM tract OD

IDP_dMRI_TBSS_OD_Superior_corona_radiata_R, 12, WM tract OD

IDP_dMRI_TBSS_OD_Superior_corona_radiata_L, 12, WM tract OD

IDP_dMRI_TBSS_OD_Posterior_corona_radiata_R, 12, WM tract OD

IDP_dMRI_TBSS_OD_Posterior_corona_radiata_L, 12, WM tract OD

IDP_dMRI_TBSS_OD_Posterior_thalamic_radiation_R, 12, WM tract OD

IDP_dMRI_TBSS_OD_Posterior_thalamic_radiation_L, 12, WM tract OD

IDP_dMRI_TBSS_OD_Sagittal_stratum_R, 12, WM tract OD

IDP_dMRI_TBSS_OD_Sagittal_stratum_L, 12, WM tract OD

IDP_dMRI_TBSS_OD_External_capsule_R, 12, WM tract OD

IDP_dMRI_TBSS_OD_External_capsule_L, 12, WM tract OD

IDP_dMRI_TBSS_OD_Cingulum_cingulate_gyrus_R, 12, WM tract OD

IDP_dMRI_TBSS_OD_Cingulum_cingulate_gyrus_L, 12, WM tract OD

IDP_dMRI_TBSS_OD_Cingulum_hippocampus_R, 12, WM tract OD

IDP_dMRI_TBSS_OD_Cingulum_hippocampus_L, 12, WM tract OD

IDP_dMRI_TBSS_OD_Fornix_cres+Stria_terminalis_R, 12, WM tract OD

IDP_dMRI_TBSS_OD_Fornix_cres+Stria_terminalis_L, 12, WM tract OD

IDP_dMRI_TBSS_OD_Superior_longitudinal_fasciculus_R, 12, WM tract OD

IDP_dMRI_TBSS_OD_Superior_longitudinal_fasciculus_L, 12, WM tract OD

IDP_dMRI_TBSS_OD_Superior_fronto-occipital_fasciculus_R, 12, WM tract OD

IDP_dMRI_TBSS_OD_Superior_fronto-occipital_fasciculus_L, 12, WM tract OD

IDP_dMRI_TBSS_OD_Uncinate_fasciculus_R, 12, WM tract OD

IDP_dMRI_TBSS_OD_Uncinate_fasciculus_L, 12, WM tract OD

IDP_dMRI_TBSS_OD_Tapetum_R, 12, WM tract OD

IDP_dMRI_TBSS_OD_Tapetum_L, 12, WM tract OD

IDP_dMRI_ProbtrackX_OD_ar_l, 12, WM tract OD

IDP_dMRI_ProbtrackX_OD_ar_r, 12, WM tract OD

IDP_dMRI_ProbtrackX_OD_atr_l, 12, WM tract OD

IDP_dMRI_ProbtrackX_OD_atr_r, 12, WM tract OD

IDP_dMRI_ProbtrackX_OD_cgc_l, 12, WM tract OD

IDP_dMRI_ProbtrackX_OD_cgc_r, 12, WM tract OD

IDP_dMRI_ProbtrackX_OD_cgh_l, 12, WM tract OD

IDP_dMRI_ProbtrackX_OD_cgh_r, 12, WM tract OD

IDP_dMRI_ProbtrackX_OD_cst_l, 12, WM tract OD

IDP_dMRI_ProbtrackX_OD_cst_r, 12, WM tract OD

IDP_dMRI_ProbtrackX_OD_fma, 12, WM tract OD

IDP_dMRI_ProbtrackX_OD_fmi, 12, WM tract OD

IDP_dMRI_ProbtrackX_OD_ifo_l, 12, WM tract OD

IDP_dMRI_ProbtrackX_OD_ifo_r, 12, WM tract OD

IDP_dMRI_ProbtrackX_OD_ilf_l, 12, WM tract OD

IDP_dMRI_ProbtrackX_OD_ilf_r, 12, WM tract OD

IDP_dMRI_ProbtrackX_OD_mcp, 12, WM tract OD

IDP_dMRI_ProbtrackX_OD_ml_l, 12, WM tract OD

IDP_dMRI_ProbtrackX_OD_ml_r, 12, WM tract OD

IDP_dMRI_ProbtrackX_OD_ptr_l, 12, WM tract OD

IDP_dMRI_ProbtrackX_OD_ptr_r, 12, WM tract OD

IDP_dMRI_ProbtrackX_OD_slf_l, 12, WM tract OD

IDP_dMRI_ProbtrackX_OD_slf_r, 12, WM tract OD

IDP_dMRI_ProbtrackX_OD_str_l, 12, WM tract OD

IDP_dMRI_ProbtrackX_OD_str_r, 12, WM tract OD

IDP_dMRI_ProbtrackX_OD_unc_l, 12, WM tract OD

IDP_dMRI_ProbtrackX_OD_unc_r, 12, WM tract OD

IDP_dMRI_TBSS_ISOVF_Middle_cerebellar_peduncle, 13, WM tract ISOVF

IDP_dMRI_TBSS_ISOVF_Pontine_crossing_tract, 13, WM tract ISOVF

IDP_dMRI_TBSS_ISOVF_Genu_of_corpus_callosum, 13, WM tract ISOVF

IDP_dMRI_TBSS_ISOVF_Body_of_corpus_callosum, 13, WM tract ISOVF

IDP_dMRI_TBSS_ISOVF_Splenium_of_corpus_callosum, 13, WM tract ISOVF

IDP_dMRI_TBSS_ISOVF_Fornix, 13, WM tract ISOVF

IDP_dMRI_TBSS_ISOVF_Corticospinal_tract_R, 13, WM tract ISOVF

IDP_dMRI_TBSS_ISOVF_Corticospinal_tract_L, 13, WM tract ISOVF

IDP_dMRI_TBSS_ISOVF_Medial_lemniscus_R, 13, WM tract ISOVF

IDP_dMRI_TBSS_ISOVF_Medial_lemniscus_L, 13, WM tract ISOVF

IDP_dMRI_TBSS_ISOVF_Inferior_cerebellar_peduncle_R, 13, WM tract ISOVF

IDP_dMRI_TBSS_ISOVF_Inferior_cerebellar_peduncle_L, 13, WM tract ISOVF

IDP_dMRI_TBSS_ISOVF_Superior_cerebellar_peduncle_R, 13, WM tract ISOVF

IDP_dMRI_TBSS_ISOVF_Superior_cerebellar_peduncle_L, 13, WM tract ISOVF

IDP_dMRI_TBSS_ISOVF_Cerebral_peduncle_R, 13, WM tract ISOVF

IDP_dMRI_TBSS_ISOVF_Cerebral_peduncle_L, 13, WM tract ISOVF

IDP_dMRI_TBSS_ISOVF_Anterior_limb_of_internal_capsule_R, 13, WM tract ISOVF

IDP_dMRI_TBSS_ISOVF_Anterior_limb_of_internal_capsule_L, 13, WM tract ISOVF

IDP_dMRI_TBSS_ISOVF_Posterior_limb_of_internal_capsule_R, 13, WM tract ISOVF

IDP_dMRI_TBSS_ISOVF_Posterior_limb_of_internal_capsule_L, 13, WM tract ISOVF

IDP_dMRI_TBSS_ISOVF_Retrolenticular_part_of_internal_capsule_R, 13, WM tract ISOVF

IDP_dMRI_TBSS_ISOVF_Retrolenticular_part_of_internal_capsule_L, 13, WM tract ISOVF

IDP_dMRI_TBSS_ISOVF_Anterior_corona_radiata_R, 13, WM tract ISOVF

IDP_dMRI_TBSS_ISOVF_Anterior_corona_radiata_L, 13, WM tract ISOVF

IDP_dMRI_TBSS_ISOVF_Superior_corona_radiata_R, 13, WM tract ISOVF

IDP_dMRI_TBSS_ISOVF_Superior_corona_radiata_L, 13, WM tract ISOVF

IDP_dMRI_TBSS_ISOVF_Posterior_corona_radiata_R, 13, WM tract ISOVF

IDP_dMRI_TBSS_ISOVF_Posterior_corona_radiata_L, 13, WM tract ISOVF

IDP_dMRI_TBSS_ISOVF_Posterior_thalamic_radiation_R, 13, WM tract ISOVF

IDP_dMRI_TBSS_ISOVF_Posterior_thalamic_radiation_L, 13, WM tract ISOVF

IDP_dMRI_TBSS_ISOVF_Sagittal_stratum_R, 13, WM tract ISOVF

IDP_dMRI_TBSS_ISOVF_Sagittal_stratum_L, 13, WM tract ISOVF

IDP_dMRI_TBSS_ISOVF_External_capsule_R, 13, WM tract ISOVF

IDP_dMRI_TBSS_ISOVF_External_capsule_L, 13, WM tract ISOVF

IDP_dMRI_TBSS_ISOVF_Cingulum_cingulate_gyrus_R, 13, WM tract ISOVF

IDP_dMRI_TBSS_ISOVF_Cingulum_cingulate_gyrus_L, 13, WM tract ISOVF

IDP_dMRI_TBSS_ISOVF_Cingulum_hippocampus_R, 13, WM tract ISOVF

IDP_dMRI_TBSS_ISOVF_Cingulum_hippocampus_L, 13, WM tract ISOVF

IDP_dMRI_TBSS_ISOVF_Fornix_cres+Stria_terminalis_R, 13, WM tract ISOVF

IDP_dMRI_TBSS_ISOVF_Fornix_cres+Stria_terminalis_L, 13, WM tract ISOVF

IDP_dMRI_TBSS_ISOVF_Superior_longitudinal_fasciculus_R, 13, WM tract ISOVF

IDP_dMRI_TBSS_ISOVF_Superior_longitudinal_fasciculus_L, 13, WM tract ISOVF

IDP_dMRI_TBSS_ISOVF_Superior_fronto-occipital_fasciculus_R, 13, WM tract ISOVF

IDP_dMRI_TBSS_ISOVF_Superior_fronto-occipital_fasciculus_L, 13, WM tract ISOVF

IDP_dMRI_TBSS_ISOVF_Uncinate_fasciculus_R, 13, WM tract ISOVF

IDP_dMRI_TBSS_ISOVF_Uncinate_fasciculus_L, 13, WM tract ISOVF

IDP_dMRI_TBSS_ISOVF_Tapetum_R, 13, WM tract ISOVF

IDP_dMRI_TBSS_ISOVF_Tapetum_L, 13, WM tract ISOVF

IDP_dMRI_ProbtrackX_ISOVF_ar_l, 13, WM tract ISOVF

IDP_dMRI_ProbtrackX_ISOVF_ar_r, 13, WM tract ISOVF

IDP_dMRI_ProbtrackX_ISOVF_atr_l, 13, WM tract ISOVF

IDP_dMRI_ProbtrackX_ISOVF_atr_r, 13, WM tract ISOVF

IDP_dMRI_ProbtrackX_ISOVF_cgc_l, 13, WM tract ISOVF

IDP_dMRI_ProbtrackX_ISOVF_cgc_r, 13, WM tract ISOVF

IDP_dMRI_ProbtrackX_ISOVF_cgh_l, 13, WM tract ISOVF

IDP_dMRI_ProbtrackX_ISOVF_cgh_r, 13, WM tract ISOVF

IDP_dMRI_ProbtrackX_ISOVF_cst_l, 13, WM tract ISOVF

IDP_dMRI_ProbtrackX_ISOVF_cst_r, 13, WM tract ISOVF

IDP_dMRI_ProbtrackX_ISOVF_fma, 13, WM tract ISOVF

IDP_dMRI_ProbtrackX_ISOVF_fmi, 13, WM tract ISOVF

IDP_dMRI_ProbtrackX_ISOVF_ifo_l, 13, WM tract ISOVF

IDP_dMRI_ProbtrackX_ISOVF_ifo_r, 13, WM tract ISOVF

IDP_dMRI_ProbtrackX_ISOVF_ilf_l, 13, WM tract ISOVF

IDP_dMRI_ProbtrackX_ISOVF_ilf_r, 13, WM tract ISOVF

IDP_dMRI_ProbtrackX_ISOVF_mcp, 13, WM tract ISOVF

IDP_dMRI_ProbtrackX_ISOVF_ml_l, 13, WM tract ISOVF

IDP_dMRI_ProbtrackX_ISOVF_ml_r, 13, WM tract ISOVF

IDP_dMRI_ProbtrackX_ISOVF_ptr_l, 13, WM tract ISOVF

IDP_dMRI_ProbtrackX_ISOVF_ptr_r, 13, WM tract ISOVF

IDP_dMRI_ProbtrackX_ISOVF_slf_l, 13, WM tract ISOVF

IDP_dMRI_ProbtrackX_ISOVF_slf_r, 13, WM tract ISOVF

IDP_dMRI_ProbtrackX_ISOVF_str_l, 13, WM tract ISOVF

IDP_dMRI_ProbtrackX_ISOVF_str_r, 13, WM tract ISOVF

IDP_dMRI_ProbtrackX_ISOVF_unc_l, 13, WM tract ISOVF

IDP_dMRI_ProbtrackX_ISOVF_unc_r, 13, WM tract ISOVF

rfMRI amplitudes (ICA25 node 1), 15, rfMRI node amplitude

rfMRI amplitudes (ICA25 node 2), 15, rfMRI node amplitude

rfMRI amplitudes (ICA25 node 3), 15, rfMRI node amplitude

rfMRI amplitudes (ICA25 node 4), 15, rfMRI node amplitude

rfMRI amplitudes (ICA25 node 5), 15, rfMRI node amplitude

rfMRI amplitudes (ICA25 node 6), 15, rfMRI node amplitude

rfMRI amplitudes (ICA25 node 7), 15, rfMRI node amplitude

rfMRI amplitudes (ICA25 node 8), 15, rfMRI node amplitude

rfMRI amplitudes (ICA25 node 9), 15, rfMRI node amplitude

rfMRI amplitudes (ICA25 node 10), 15, rfMRI node amplitude

rfMRI amplitudes (ICA25 node 11), 15, rfMRI node amplitude

rfMRI amplitudes (ICA25 node 12), 15, rfMRI node amplitude

rfMRI amplitudes (ICA25 node 13), 15, rfMRI node amplitude

rfMRI amplitudes (ICA25 node 14), 15, rfMRI node amplitude

rfMRI amplitudes (ICA25 node 15), 15, rfMRI node amplitude

rfMRI amplitudes (ICA25 node 16), 15, rfMRI node amplitude

rfMRI amplitudes (ICA25 node 17), 15, rfMRI node amplitude

rfMRI amplitudes (ICA25 node 18), 15, rfMRI node amplitude

rfMRI amplitudes (ICA25 node 19), 15, rfMRI node amplitude

rfMRI amplitudes (ICA25 node 20), 15, rfMRI node amplitude

rfMRI amplitudes (ICA25 node 21), 15, rfMRI node amplitude

rfMRI amplitudes (ICA100 node 1), 15, rfMRI node amplitude

rfMRI amplitudes (ICA100 node 2), 15, rfMRI node amplitude

rfMRI amplitudes (ICA100 node 3), 15, rfMRI node amplitude

rfMRI amplitudes (ICA100 node 4), 15, rfMRI node amplitude

rfMRI amplitudes (ICA100 node 5), 15, rfMRI node amplitude

rfMRI amplitudes (ICA100 node 6), 15, rfMRI node amplitude

rfMRI amplitudes (ICA100 node 7), 15, rfMRI node amplitude

rfMRI amplitudes (ICA100 node 8), 15, rfMRI node amplitude

rfMRI amplitudes (ICA100 node 9), 15, rfMRI node amplitude

rfMRI amplitudes (ICA100 node 10), 15, rfMRI node amplitude

rfMRI amplitudes (ICA100 node 11), 15, rfMRI node amplitude

rfMRI amplitudes (ICA100 node 12), 15, rfMRI node amplitude

rfMRI amplitudes (ICA100 node 13), 15, rfMRI node amplitude

rfMRI amplitudes (ICA100 node 14), 15, rfMRI node amplitude

rfMRI amplitudes (ICA100 node 15), 15, rfMRI node amplitude

rfMRI amplitudes (ICA100 node 16), 15, rfMRI node amplitude

rfMRI amplitudes (ICA100 node 17), 15, rfMRI node amplitude

rfMRI amplitudes (ICA100 node 18), 15, rfMRI node amplitude

rfMRI amplitudes (ICA100 node 19), 15, rfMRI node amplitude

rfMRI amplitudes (ICA100 node 20), 15, rfMRI node amplitude

rfMRI amplitudes (ICA100 node 21), 15, rfMRI node amplitude

rfMRI amplitudes (ICA100 node 22), 15, rfMRI node amplitude

rfMRI amplitudes (ICA100 node 23), 15, rfMRI node amplitude

rfMRI amplitudes (ICA100 node 24), 15, rfMRI node amplitude

rfMRI amplitudes (ICA100 node 25), 15, rfMRI node amplitude

rfMRI amplitudes (ICA100 node 26), 15, rfMRI node amplitude

rfMRI amplitudes (ICA100 node 27), 15, rfMRI node amplitude

rfMRI amplitudes (ICA100 node 28), 15, rfMRI node amplitude

rfMRI amplitudes (ICA100 node 29), 15, rfMRI node amplitude

rfMRI amplitudes (ICA100 node 30), 15, rfMRI node amplitude

rfMRI amplitudes (ICA100 node 31), 15, rfMRI node amplitude

rfMRI amplitudes (ICA100 node 32), 15, rfMRI node amplitude

rfMRI amplitudes (ICA100 node 33), 15, rfMRI node amplitude

rfMRI amplitudes (ICA100 node 34), 15, rfMRI node amplitude

rfMRI amplitudes (ICA100 node 35), 15, rfMRI node amplitude

rfMRI amplitudes (ICA100 node 36), 15, rfMRI node amplitude

rfMRI amplitudes (ICA100 node 37), 15, rfMRI node amplitude

rfMRI amplitudes (ICA100 node 38), 15, rfMRI node amplitude

rfMRI amplitudes (ICA100 node 39), 15, rfMRI node amplitude

rfMRI amplitudes (ICA100 node 40), 15, rfMRI node amplitude

rfMRI amplitudes (ICA100 node 41), 15, rfMRI node amplitude

rfMRI amplitudes (ICA100 node 42), 15, rfMRI node amplitude

rfMRI amplitudes (ICA100 node 43), 15, rfMRI node amplitude

rfMRI amplitudes (ICA100 node 44), 15, rfMRI node amplitude

rfMRI amplitudes (ICA100 node 45), 15, rfMRI node amplitude

rfMRI amplitudes (ICA100 node 46), 15, rfMRI node amplitude

rfMRI amplitudes (ICA100 node 47), 15, rfMRI node amplitude

rfMRI amplitudes (ICA100 node 48), 15, rfMRI node amplitude

rfMRI amplitudes (ICA100 node 49), 15, rfMRI node amplitude

rfMRI amplitudes (ICA100 node 50), 15, rfMRI node amplitude

rfMRI amplitudes (ICA100 node 51), 15, rfMRI node amplitude

rfMRI amplitudes (ICA100 node 52), 15, rfMRI node amplitude

rfMRI amplitudes (ICA100 node 53), 15, rfMRI node amplitude

rfMRI amplitudes (ICA100 node 54), 15, rfMRI node amplitude

rfMRI amplitudes (ICA100 node 55), 15, rfMRI node amplitude

rfMRI connectivity ICA-features 1, 16, rfMRI connectivity

rfMRI connectivity ICA-features 2, 16, rfMRI connectivity

rfMRI connectivity ICA-features 3, 16, rfMRI connectivity

rfMRI connectivity ICA-features 4, 16, rfMRI connectivity

rfMRI connectivity ICA-features 5, 16, rfMRI connectivity

rfMRI connectivity ICA-features 6, 16, rfMRI connectivity

QC_T1-to-standard_linear_alignment_discrepancy, 0, QC

QC_T1-to-standard_nonlinear_alignment_discrepancy, 0, QC

QC_T1-to-standard_nonlinear_alignment_warping, 0, QC

QC_T1_inverse_SNR, 0, QC

QC_T1_inverse_CNR, 0, QC

QC_T2_FLAIR-to-T1_linear_alignment_discrepancy, 0, QC

QC_dMRI-to-T1_linear_alignment_discrepancy, 0, QC

QC_SWI-to-T1_linear_alignment_discrepancy, 0, QC

QC_rfMRI-to-T1_linear_alignment_discrepancy, 0, QC

QC_rfMRI_head_motion, 0, QC

QC_rfMRI_inverse_tSNR, 0, QC

QC_rfMRI_cleaned_inverse_tSNR, 0, QC

QC_dMRI_eddy_outlier_slices, 0, QC

IDP_T2_FLAIR_BIANCA_periventWMH_volume, 6, white matter hyperintensity volume

IDP_T2_FLAIR_BIANCA_deepWMH_volume, 6, white matter hyperintensity volume

ASL_mPLD_perfusion_calib_gm_mean, 21, ASL perfusion

ASL_GE_perfusion_calib_gm_mean, 21, ASL perfusion
