# Supplementary material for: Reliability of multi-site UK Biobank MRI brain phenotypes for the assessment of neuropsychiatric complications of SARS-CoV-2 infection: The COVID-CNS travelling heads study
Source: PLoS One. 2022 Sep 29;17(9):e0273704. doi: 10.1371/journal.pone.0273704 (PMC9522299; doi:10.1371/journal.pone.0273704)
Supplement: Attachment — Submitted filename: Response_Reviewers_1.docx [file pone.0273704.s006.docx]

6 June 2022

We thank the reviewers for their careful assessment of our manuscript. Below we describe our corrections and responses to the Reviewer comments.

**Reviewer #1**

In this manuscript the reliability of features extracted from multi-modal MRIs were evaluated across different sites and scanners, by scanning 8 participants at 4 UK sites: 3 using Siemens PRISMA scanners (Cambridge, Liverpool, Oxford) and 1 using a GE scanner (King’sCollege London). It gave confidence that large, multi-site MRI datasets can be
collected reliably at different sites across the diverse range of MRI modalities and IDPs
that could be mechanistically informative in COVID brain research. This work is important to give guidelines on how to handle the cross-site/cross-scanner data heterogeneity of various MRI phenotypes.

Ref 1/1: The major limitation of the paper is the small sample size---only 8 participants were considered. With such a small sample size, the statistical analysis should be more rigid.

*The repeated measures “travelling heads” design provided 32 scans from 8 participants across 4 sites. This number of scans provided sufficient sample size for the assessment of large-scale variations in IDP ICCs (with statistics derived from resampling 2,817 UK Biobank repeat-scan subjects) and identification of any substantial site-effects affecting IDPs using repeated measures ANOVAs. We have used rigorous ANOVA-based statistical methods to analyse these data. We refer now to power analysis results that identify the scale of effects we can expect to identify, and confirm that similar results are obtained with non-parametric techniques (see response to Ref 1/6). We have clarified the descriptions of our statistical analyses in the Methods section:*

Site effects were assessed using repeated-measures ANOVAs, testing the null hypotheses of zero between-site difference in mean IDPs. Normality was assessed with Shapiro-Wilk, and sphericity was assessed using Mauchly’s test. A lack of sphericity indicates a violation of ANOVA assumptions, an independent effect of site on IDP measures, i.e., between-site. differences in variation of an IDP. A Greenhouse-Geisser correction for sphericity was applied if sphericity was detected. These tests were made for each of 2243 (total) IDPs (excluding IDPs representing individual functional network connections). A power analysis indicated that the available subject numbers would permit the reliable identification of “large” site effects (eta-squared = 0.2) with power = 0.87, while smaller site effects (eta-squared = 0.1) would be less reliably detected (power = 0.51). When assessing different patterns of effects across different classes of IDPs, we used the false discovery rate (FDR=5%, within each class of IDP), to control type 1 errors. Site-specific effects on each IDP were estimated twice: once using all the analysable data (from 4 sites, including 1 GE site), and once using only Siemens data (from 3 sites). This allowed us to investigate site-differences in IDP location or sphericity that were likely related to between-manufacturer differences in MRI scanners.

*We have also clarified our random-sampling based methods for statistical testing using the UK Biobank data:*

To benchmark the between-subject and between-site reliability of each IDP measured using the COVID-CNS protocol, we compared these ICCs from the travelling heads study to comparable ICCs estimated in the UKB cohort. In this design, N=2,817 largely healthy middle-aged participants were each scanned twice (with mean between-scan interval = 2.25 y; SD = 0.12) at the same one of 4 possible sites, all using the same scanner for multi-modal MRI (Siemens Skyra 3T). As noted, the MRI sequences for COVID-CNS were based on similar or identical sequences for T1, T2 FLAIR, dMRI, swMRI and fMRI used in the UKB enhancement cohort (**Table 1**). Hence, we could directly compare test-retest and between-site consistency of IDPs measured in the UKB and COVID-CNS cohorts. We estimated ICCs between the test and retest IDP measurement vectors for N=8 participants, repeatedly, randomly sampled from the total UKB dataset (N = 2,817; 1000 random samples). This allowed us to estimate the distribution of ICCs for repeated MRI measurements on N=8 participants using data with no site or scanner contributions to variance. From these resampled distributions, we computed *P*-values for a two-sided test of the null hypothesis that the ICCs estimated from the travelling heads experimental data were sampled from the same distribution as the ICCs estimated from repeated measurements on comparable sub-samples of the UKB cohort.

Ref 1/2: It seems like I cannot find the confidence intervals in tables or Figures of the ICCs.

*We acknowledge that our description of confidence intervals in the text was unclear. It was relating to the generation of P-values from random re-sampling of the UK Biobank data, as described above. We have clarified this text (see response to Ref 1/1). Confidence intervals are shown in the violin plots representing the UK Biobank N=8 ICC distributions in our Figures. We now explicitly identify these confidence intervals in Figure captions, e.g.:*

The grey violin plots in both panels indicate the equivalent distributions of T1 inverse SNR and CNR, respectively, in the UK Biobank reference dataset, using matched random sampling of N=8 participants. Box and whiskers represent inter-quartile range and 95% confidence intervals, respectively.

Ref 1/3: Have some confounding factors removed from the IDP similar to what UK Biobank did, such as motion parameters from rest fMRI, ICV from brain regional volume etc.? The authors needed to describe in details.

*We have now clarified this in the text:*

[Methods:fMRI]

FIX identifies and removes artefacts related to measured head-motion, cardio-vascular cycles, acquisition variability and other phenomena.

[Methods: Statistical analysis and UK Biobank benchmarking]

Biobank-style deconfounding was not possible here due to the low subject numbers repeated-measures design. Additionally, translation of Biobank deconfounding estimates was not possible as our pilot cohort differs considerably from the demographic profile of the UKB cohort. However, these deconfounding procedures will be implemented in the analyses of the principal study data from the COVID-CNS consortium.

Ref 1/4: The author should better provide more demographic information about the 8 participants, for example, are they British, percentages of males/females?

*We have provided basic information regarding sex, age, height and weight of our travelling heads participants:*

[Study design and sample]

Participant age, sex, height and weight statistics are presented in Supplementary Table 1.

*Unfortunately, we do not have more detailed demographic data available on the travelling heads study participants We comment on the relevance of demographics in our Discussion:*

The sample for the travelling heads experimental study was considerably younger than the UK Biobank population, and the expected COVID-CNS consortium cohort of post-hospitalisation patients. It comprised 7 females and 1 male, and additional demographic data were not obtained. While we expect the relative youth of the travelling heads sample to have negligible impact on our assessment of between-site reliability of multimodal MRI, this demographic disparity precludes more detailed comparisons between these data and technically comparable data on the UK Biobank and COVID-CNS cohorts.

Ref 1/5: Could the authors show the scatterplots of UKBiobank reliabilities versus your IDP reliabilities for both the cases of all scanners and Siemens only? They should be highly correlated if they are correct.

*We thank the reviewer for this suggestion. We have added the following material to the main text and supplementary information.*

[Results: T1w and T2 Flair] Overall, across all modalities, ICC values were correlated across UK Biobank and travelling heads samples. Across all IDPs, r=0.43 for Siemens scanners only and r=0.39 when including the GE site. With a large proportion of IDPs having consistently high ICCs (e.g. >0.7), these correlations are estimated from a relatively low level of variation in ICCs across IDPs (see Supplementary Figure S1).

[Caption] S1. Scatterplots of ICCs for IDPs derived from UK Biobank repeat-scan data and Travelling Heads dataset (a). Siemens sites only (r=0.43). (b). All sites (r=0.39). Each point corresponds to an individual IDP.

Ref 1/6: As the sample size is too small, the author may use more non-parametric statistical test instead of those test with Gaussian assumptions.

Evidence of violations of ANOVA assumptions was generally limited. However, we have now added more description of where normality and sphericity tests were violated and we have added new analysis to corroborate our results from normal parametric testing with a non-parametric alternative to the repeated measures ANOVA, namely the rank-based Friedman test. We have preferred to retain standard ANOVA as the principal method for analysis of between-site differences in the main text, but we have added a new figure to the supplementary information which allows a direct comparison of the similar scale and distribution of *P*-values for between-site differences estimated by parametric (ANOVA) and non-parametric (Friedman) tests.

[Results: T1w and T2-FLAIR images ] To confirm our statistical results given the limited sample size, we repeated our analysis using the parametric Friedman’s test. This produced very similar distributions of *P*-values across IDPs (Supplementary Figure S2).

[Caption] S2. Comparison of FDR-corrected *P*-values derived using repeated measures ANOVA (orange) and non-parametric Friedman (green) tests.

[Results: T1w and T2-FLAIR images ] Sphericity tests also pointed to altered variance at the GE site for a proportion of tissue intensity (23%) and grey-white contrast (34%) IDPs.

[Results: dMRI] Some WM tract ICVF (23%) and ISOVF (20%) diffusion parameter IDPs showed evidence of non-normality.

[ Results: fMRI ] These IDPs in general did not show site-specific variations in mean (Fig 6A), although two of the six (2,6) showed evidence of sphericity violations.

Minor issues are listed in the following.

Ref 1/7: In page 19, for fMRI traits, why there are 3464 IDPs? could the author explain. Similarly for dMRI, the author considered FA, MO, MD, ICVF, ISOVF, ODI, and there may probably be (48+27)*6 but why there are 675 IDPs?

*We have expanded the text describing the IDPs assessed. The complete list of IDPs is now provided in supplementary information.We have now added more detail to our description of the fMRI IDPs. Our calculation included the individual fMRI nodes. However, as these were summarised in the results within the 6 connectivity modes, we have adjusted our text:*

[Methods fMRI] Two sets of resting-state networks derived from group ICA decompositions of UKB reference data (25 and 100 component decompositions with 21 and 55 neural components respectively) were projected onto the pre-processed resting state fMRI data in a dual-regression analysis (29). Whole brain functional connectivity matrices were compiled from full and partial correlations (210 and 1485 elements for 25 and 100 component decompositions respectively). The amplitudes (standard deviations) of spontaneous activity at each regional node were also estimated (21 and 55 elements) (10). As individual connections showed low test-retest reliability in the UKB dataset, we used a dimension-reduction approach which applied ICA to all functional connectivity IDPs to produce 6 primary modes of variation (15). These six modes were projected onto the individual’s functional connectivity matrix and used as additional IDPs. Finally four fMRI QC IDPs were defined (alignment discrepancy, head motion, initial and FIX cleaned tSNR). Excluding the individual connectivity nodes, 86 IDPs were assessed from the fMRI data.

*For the diffusion data, we also included L1, L2 and L3 eigenvalues of the diffusion tensor, which we have added to the text.*

[Methods dMRI] The multi-shell data were processed with NODDI (Neurite Orientation Dispersion and Density Imaging) (31), to produce microstructural parameters including ICVF (intra-cellular volume fraction**,** an index of white matter neurite density), ISOVF (isotropic or free water volume fraction), L1, L2 and L3 eigenvalues of the diffusion tensor**,** and ODI (orientation dispersion index; a measure of within-voxel tract disorganisation).

Ref 1/8: to correct some typos. For example, for standard deviation, the author uses "sd" somewhere while uses "SD" in other places.

*Thank you for picking this up. We have corrected the text for notational consistency.*

Ref 1/9: There are four rows in Figure 6. Does the subfigures in row 3 belong to Figure 6 C) or Figure 6 B)? Please make it clear.

*Thank you for picking this up. The “C.” marker was incorrectly positioned, and this has now been corrected.*

**Reviewer #2:**

This paper presents a multi-modal MRI protocol to reliably scan post-COVID patients across different sites. More than 2,000 IDPs were obtained by processing the acquired T1w, T2w, dMRI, rs-fMRI, sw-MRI, and ASL images. Between-site reliability of these IDPs were tested using intra-class correlations (ICC) and compared against the test-retest reliability of the same IDPs in scans from UK Biobank. The results for sites with same scanner manufacturer are more reliable compared to the one with different scanner manufacturer. Thus, reliability of the proposed multi-modal MRI protocol is limited to same type of scanner, which may not guarantee comparable data acquired using different scanner types.
I have following questions:

Ref 2/1. The authors mentioned that 1,073 IDPs were obtained from T1w and T2-FLAIR scans. Can they list few IDPs that were obtained? And what are the metrics of subcortical segmentation?

*The full list of IDPs is now provided in the supplementary material. We have also expanded our description of these IDPs in the main text.*

Ref 2/2: For post-acquisition processing of structural MRIs, why a standardized distortion correction method not applied to scans from Siemens and GE scanners?

*We aimed to provide the best possible data processing that was consistent across the different scanners at each site. The Siemens distortion correction method required was not possible to implement consistently from the data provided by GE scanners. We clarify this now in the text:*

[Methods] Implementing the off-scanner 3D gradient distortion correction for Siemens data was not possible for data acquired on the GE scanner. For the sake of consistency of data pre-processing pipelines across scanner manufacturers, a standard GE gradient distortion correction method was implemented for all data.

Ref 2/3. “To avoid dependence on site-specific T1w data, we used T1w data from all sites to define generic masks for estimation of mean grey matter CBF and ATT.” How are these generic masks obtained? Are multi-site T1w data spatially normalized to a common space to get mask?

*Yes, the reviewer has correctly inferred how the generic masks were constructed. We now clarify this point in the text:*

[Methods ASL] To avoid dependence on site-specific T1w data, T1w grey matter masks derived from each scan site were normalised into MNI space to identify voxels present in all masks for the estimation of mean grey matter CBF and ATT.

Ref 2/4: Are dMRI data collected with single phase encoding?

*Yes, AP phase encoding was used as per the UK Biobank. A short acquisition of reversed PA encoded data is acquired to enable B0 distortion correction. We have added this to the acquisition description table:*

[Table 1] TR=3600 ms, 50 dirs/shell, b=0, 1000 2000 s/mm2, MB 3 blip-reversed b=0. AP phase encoding, 36s of PA encoding b=0 for EPI distortion correction.

Ref 2/5. How are the weighted-mean summaries of diffusion parameters for each tract computed?

These were calculated using PROBTRACKx tractography parameters. We have expanded on this description:

[Methods: dMRI] These parameters were summarised using two approaches: first, using a white-matter tract skeleton analysis producing average values for 48 standard-space tract masks (32); and second, using probabilistic tractography: BEDPOSTx was used to model multi-fibre tract orientation structure and PROBTRACKx for probabilistic tractography with crossing fibre modelling (10). For 27 major tracts defined in AutoPtx (http://fsl.fmrib.ox.ac.uk/fsl/fslwiki/AutoPtx), the posterior mean fractional voxel occupancy defined the weights for weighted-mean estimates of each DTI/NODDI parameter (10).

Ref 2/6. Can the reliability of IDPs be improved using post-acquisition harmonization?

*Yes, the reliability of IDPs can be further improved by post-acquisition processing. This is beyond the scope of the current paper however we have added additional commentary on this point to the discussion:*

[Discussion] Between-site offsets in the mean and scaling of IDP values could be corrected statistically post hoc by harmonisation or modelling methods such as COMBAT(38) or Generalised Additive Modelling (39). These approaches can require good numbers of patients and controls at each site, which may limit multi-site clinical studies to sites that can recruit good numbers of participants.
