# Supplementary material for: Reliability of multi-site UK Biobank MRI brain phenotypes for the assessment of neuropsychiatric complications of SARS-CoV-2 infection: The COVID-CNS travelling heads study
Source: PLoS One. 2022 Sep 29;17(9):e0273704. doi: 10.1371/journal.pone.0273704 (PMC9522299; doi:10.1371/journal.pone.0273704)
Supplement: Attachment — Submitted filename: Response_Reviews_rev2.docx [file pone.0273704.s007.docx]

**Reliability of multi-site UK Biobank MRI brain phenotypes for the assessment of neuropsychiatric complications of SARS-CoV-2 infection: the COVID-CNS travelling heads study**

Thank you for your careful assessment by of this submission. We have made the following changes requested to the manuscript:

Review comments to the author reviewer #1:

*One of my minor concern is the resolution/quality of many of your figures are low, for example, Figure 2~6. I cannot see clearly the axis labels/fonts/numbers in many places of the figures. Please improve the figure resolution and increase those font sizes.*

We have edited figures 2-7 to ensure the clarity of all text within.

We have also reviewed our references and have updated cited preprints that have now been accepted for publication.
